# Structure-Activity Investigation of a G Protein-Biased Agonist Reveals Molecular Determinants for Biased Signaling of the D_2_ Dopamine Receptor

**DOI:** 10.3389/fnsyn.2018.00002

**Published:** 2018-02-21

**Authors:** Lani S. Chun, Rakesh H. Vekariya, R. Benjamin Free, Yun Li, Da-Ting Lin, Ping Su, Fang Liu, Yoon Namkung, Stephane A. Laporte, Amy E. Moritz, Jeffrey Aubé, Kevin J. Frankowski, David R. Sibley

**Affiliations:** ^1^Molecular Neuropharmacology Section, National Institute of Neurological Disorders and Stroke, National Institutes of Health, Bethesda, MD, United States; ^2^Department of Medicinal Chemistry and Specialized Chemistry Center, University of Kansas, Lawrence, KS, United States; ^3^Neural Engineering Unit, Behavior Neuroscience Research Branch, National Institute on Drug Abuse, National Institutes of Health, Baltimore, MD, United States; ^4^Molecular Neuroscience, Campbell Family Mental Health Research Institute, Centre for Addiction and Mental Health, University of Toronto, Toronto, ON, Canada; ^5^Department of Medicine, McGill University Health Center Research Institute, McGill University, Montreal, QC, Canada

**Keywords:** D_2_ dopamine receptor, functional selectivity, biased signaling, structure-activity relationship, MLS1547, quinoline

## Abstract

The dopamine D2 receptor (D2R) is known to elicit effects through activating two major signaling pathways mediated by either G proteins (Gi/o) or β-arrestins. However, the specific role of each pathway in physiological or therapeutic activities is not known with certainty. One approach to the dissection of these pathways is through the use of drugs that can selectively modulate one pathway vs. the other through a mechanism known as functional selectivity or biased signaling. Our laboratory has previously described a G protein signaling-biased agonist, MLS1547, for the D2R using a variety of *in vitro* functional assays. To further evaluate the biased signaling activity of this compound, we investigated its ability to promote D2R internalization, a process known to be mediated by β-arrestin. Using multiple cellular systems and techniques, we found that MLS1547 promotes little D2R internalization, which is consistent with its inability to recruit β-arrestin. Importantly, we validated these results in primary striatal neurons where the D2R is most highly expressed suggesting that MLS1547 will exhibit biased signaling activity *in vivo*. In an effort to optimize and further explore structure-activity relationships (SAR) for this scaffold, we conducted an iterative chemistry campaign to synthesize and characterize novel analogs of MLS1547. The resulting analysis confirmed previously described SAR requirements for G protein-biased agonist activity and, importantly, elucidated new structural features that are critical for agonist efficacy and signaling bias of the MLS1547 scaffold. One of the most important determinants for G protein-biased signaling is the interaction of a hydrophobic moiety of the compound with a defined pocket formed by residues within transmembrane five and extracellular loop two of the D2R. These results shed new light on the mechanism of biased signaling of the D2R and may lead to improved functionally-selective molecules.

## Introduction

The D_2_ dopamine receptor (D2R) is one of the most validated drug targets in the treatment of psychiatric and neurological diseases. Unfortunately, many of the drugs that target the D2R exhibit adverse side effects, which can be both on and off target in nature. The D2R stimulates both G protein- and β-arrestin-mediated pathways to elicit a variety of downstream signaling effects (Beaulieu and Gainetdinov, 2011). Activation of Gi/o proteins inhibits adenylyl cyclase-mediated cAMP production (Sibley and Monsma, 1992; Missale et al., 1998) whereas activated Gβγ subunits inhibit L/N-type calcium channels (Yan et al., 1997; Hernandez-Lopez et al., 2000) and activate G protein-coupled inward rectifying potassium (GIRK) channels (Kuzhikandathil et al., 1998; Lavine et al., 2002; Luscher and Slesinger, 2010). D2R activation also triggers the recruitment of β-arrestin-2 (also referred to as arrestin-3) to the receptor resulting in D2R internalization (Skinbjerg et al., 2009) and activation of β-arrestin-mediated signaling cascades leading to glycogen synthase kinase-3 (GSK3) activation and downstream signaling (Beaulieu et al., 2007, 2015). This complicated milieu of dopamine-mediated signaling makes it difficult to discern which of these pathways are important for D2R-related pathology and/or treatment goals. Dissection of these pathways is thus a primary objective in understanding the physiological effects of D2R signaling while the selective targeting of specific pathways may enhance the therapeutic efficacy and side-effect profiles of D2R-related drugs.

Most agonists, in particular the endogenous transmitter, will activate all signaling pathways of a particular receptor with equal or similar efficacies. However, it is now recognized that some agonists may preferentially activate one signaling pathway vs. another or even activate one while inhibiting another. This phenomenon, referred to as functional selectivity or biased signaling has been observed for many different GPCRs and found to encompass not only different signaling pathways, but also other agonist-induced responses such as desensitization/down-regulation (Galandrin et al., 2007; Kenakin, 2007; Leach et al., 2007; Mailman, 2007; Urban et al., 2007; Violin and Lefkowitz, 2007; Evans et al., 2011). While the mechanisms underlying biased signaling are not known with certainty, a leading hypothesis is that GPCRs can adopt multiple functionally active conformational states that are stabilized or induced by selective ligands (Kobilka and Deupi, 2007; Wess et al., 2008; Bokoch et al., 2010). Structural data supporting hypotheses for biased activation of GPCRs have recently been described (Liu et al., 2012; Rahmeh et al., 2012; Wacker et al., 2013), suggesting that the rational design of functionally-selective or signaling-biased compounds may be achievable.

Like many GPCRs, the D2R can exhibit biased signaling in response to agonists that are functionally selective (Lawler et al., 1999; Mailman, 2007) including those that are biased for stimulating either G protein- (Free et al., 2014; Moller et al., 2014, 2017; Weichert et al., 2015; Chen et al., 2016; Bonifazi et al., 2017) or β-arrestin-mediated signaling (Allen et al., 2011; Chen et al., 2012; Hiller et al., 2013; Weichert et al., 2015; Mannel et al., 2017a,b). The growing availability of these pharmacological tools is expected to greatly assist in determining the physiological roles and therapeutic significance of D2R-stimulated G protein and β-arrestin-mediated signaling. Using a variety of *in vitro* functional assays, we previously described one such G protein-biased D2R agonist, MLS1547, which was found to be highly efficacious for G protein-mediated signaling (inhibition of cAMP production), but relatively ineffective for recruiting β-arrestin (Free et al., 2014). Over two dozen MLS1547 analogs with varying degrees of signaling bias were further characterized and used to computationally develop a pharmacophore model to explain the biased signaling properties of this chemical scaffold. In brief, it was postulated that the scaffold must contain a hydrophobic binding moiety, two aromatic groups, and a positively charged nitrogen all in the correct orientation (Free et al., 2014). Further, molecular dynamics simulations using an active state model of the D2R suggested a binding pose where biased, but not unbiased, ligands interact with a hydrophobic pocket formed by residues near the extracellular end of the 5th transmembrane domain (TM5) of the receptor (Free et al., 2014). These interactions have been hypothesized to affect the tilting of TM5, which might bias the D2R to preferentially activating G proteins vs. recruiting β-arrestin (Free et al., 2014).

In the current study, we sought to further characterize the biased signaling properties of the MLS1547 scaffold through examining its ability to modulate D2R internalization, which is mediated by β-arrestin-2 (Skinbjerg et al., 2009). Importantly, these assays were extended to primary neuronal cell model systems, which more likely replicate the cellular milieu in which the D2R normally propagates intracellular signals. Interestingly, the biased signaling properties of MLS1547 that were observed in heterologous expression systems were largely replicated in the neuronal model systems. We also sought to further elucidate the structure-activity relationships (SAR) for the biased signaling properties of MLS1547 through the synthesis and evaluation of 50 MLS1547 analogs. These compounds were examined in both G protein- and β-arrestin-mediated signaling assays and were found to exhibit a wide range of D2R agonist activities. These analyses confirmed previously described SAR requirements for G protein-biased agonist activity and, importantly, elucidated new structural features that are critical for agonist activity and G protein-mediated signaling bias of the MLS1547 scaffold.

## Materials and methods

### Materials

MLS1547 was originally obtained from the NIH Molecular Libraries Screening Center Network Library and subsequently purchased from MolPort (Riga, Latvija) for follow-up triage studies. Subsequent batches of the compound, along with all analogs, were synthesized at the University of Kansas Specialized Chemistry Center. All compounds were evaluated via NMR to confirm purity (>90%) and structural veracity. Identical results were obtained with all batches from all sources. All other chemicals were obtained from Sigma-Aldrich (St. Louis, MO) unless otherwise indicated. All tissue culture media and materials were obtained from Corning/Mediatech, Inc. (Manassas, VA).

### cAMP inhibition assay

Assays were performed as previously described (Free et al., 2014). Briefly, D2R-mediated inhibition of forskolin-stimulated cAMP production was assayed using the DiscoverX HitHunter assay kit (DiscoverX, Inc., Fremont, CA). CHO-K1 cells stably expressing the human D2R long isoform were purchased from DiscoverX, Inc. and maintained in Ham's F12 supplemented with 10% fetal bovine serum, 100 U/ml penicillin, 100 μg/ml streptomycin, 20 mM HEPES, and 800 μg/ml G418 at 37°C, 5% CO_2_, and 90% humidity. Cells were seeded in Cell Plating Media 2 (DiscoverX, Inc.) at a density of 5,000 cells/well in 384-well black, clear-bottom plates. After 16–24 h, medium was removed and replaced with 5 μl/well phosphate-buffered saline (PBS). Compounds were diluted in PBS in the presence of 100 μM forskolin and 0.2 μM sodium metabisulfite. Cells were treated with 2.5 μl of various concentrations of compound and incubated for 60 min at 37°C. ED, lysis buffer, cAMP antibody, and emerald II reagents were added as directed by the manufacturer and cells were incubated for 1 h at room temperature. EA reagent was added to the plates and luminescence (RLU) was measured using an FDSS μCell (Hamamatsu Photonics K.K., Bridgewater, NJ) following a 3 h incubation in the dark at room temperature. Data were collected as relative luminescence units (RLUs), and values were normalized to a percentage of the control luminescence seen with a maximum concentration of dopamine for agonist mode assays. The Hill coefficients of the concentration-response curves did not significantly differ from unity with the data fitting to a single site model.

### β-arrestin recruitment assay

Assays were conducted with minor modifications as previously published by our laboratory (Banala et al., 2011; Bergman et al., 2013; Free et al., 2014; Meade et al., 2015), using the DiscoverX PathHunter technology (DiscoverX, Inc., Fremont, CA). Briefly, CHO-K1 cells expressing the D2R long isoform (DiscoverX, Inc.) were maintained in Ham's F12 media supplemented with 10% fetal bovine serum, 100 U/ml penicillin, 100 μg/ml streptomycin, 20 mM HEPES, and 800 μg/ml G418 and 300 μg/ml hygromycin at 37°C, 5% CO_2_, and 90% humidity. For assay, the cells were seeded in Cell Plating Media 2 (DiscoverX, Inc.) at a density of 2,625 cells/well in 384-well black, clear-bottom plates. Compounds were diluted in PBS in the presence of 0.2 μM sodium metabisulfite. Following 24 h of incubation, the cells were treated with multiple concentrations of compound and incubated at 37°C for 90 min. DiscoverX reagent was then added to cells according to the manufacturer's recommendations followed by a 45 min incubation at room temperature. Luminescence was measured on a Hamamatsu FDSS μCell reader. Data were collected as RLUs and subsequently normalized to a percentage of the control luminescence seen with a maximum concentration of dopamine for agonist mode assays. The Hill coefficients of the concentration-response curves did not significantly differ from unity.

### Primary glia and striatal neuron culture

Isolation and culturing of cells was conducted as described previously (Li et al., 2012b; Daly et al., 2015) using C57BL6/J mice. All experiments using laboratory animals were conducted with approved protocols from the NIH/NIDA Animal Care and Use Committee.

#### Glia cultures

Dissection buffer consisting of ACSF: 119 mM NaCl, 5 mM KCl, 1 mM MgCl_2_, 30 mM dextrose, 25 mM HEPES, pH 7.4, without calcium) and glia media (DMEM supplemented with 10% FBS, 10 U/ml penicillin, 10 μg/ml streptomycin, and Glutamax) were prepared and stored at 4°C until ready for use. Embryonic or neonatal C57BL6/J mice were euthanized by rapid decapitation. Cerebral cortices were dissected and placed into 15 ml concial tubes with 5–10 ml of ice-cold dissection buffer. Tissue was then digested in 2 ml of papain solution [100 μl papain, 20 mg/ml slurry (Worthington Biochemical Corp.), 20 μl of 1% DNase I (Sigma, cat. no. DN25-10MG), and 2 ml dissection buffer] for 20 min at 37°C. Following digestion, tissue was rinsed with 10 ml pre-warmed glia media, triturated with a fire-polished glass Pasteur pipette, diluted in glia media and filtered through a 70 μm cell-strainer. Cells were then seeded into collagen coated T75 flasks (2–3 embryos/flask) and placed into a 37°C cell culture incubator overnight with media replacement the next day. Cells were dissociated with 0.05% trypsin when nearing confluence in 10–12 days. Glia were cultured directly onto collagen coated inserts (Millipore PICM03050) placed in six-well plates (one confluent T75 flask per 6-well plate/2 ml/insert) and used for conditioning neuronal cultures.

#### Neuronal cultures

Pregnant C57BL6/J mice (E15-18) were euthanized with CO_2_, and embryonic mice were euthanized by decapitation. Striata were dissected from the brain and placed into a 15 ml conical tube with ice-cold dissection buffer. Tissues were dissociated with papain as described above and the resulting cells quantified. Neurons were then seeded onto poly-L-lysine coated glass coverslips in plating media: Neurobasal media (ThermoFisher, Inc.) supplemented with 5% heat-inactivated horse serum, 10 units/ml penicillin, 10 μg/ml streptomycin, Glutamax; 2% B27 supplement at a density of 0.4 × 10^6^ cells/well of a six-well plate, and cultured in a 37°C incubator overnight (neurons are DIV 0). The next day (DIV 1), coverslips containing neurons were placed underneath the glia culture inserts, one coverslip/well.

Primary striatal neurons were transfected 7–8 days after culturing with 1 μg of D2R-pHluorin DNA construct (Li et al., 2012a) using 2 μl of Lipofectamine 2000 (Invitrogen, Carlsbad, CA) according to the manufacturer's recommendations for each neuronal culture/coverslip. Briefly, Lipofectamine 2000 reagent and DNA construct were diluted in supplement-free Neurobasal medium and incubated for 20 min at room temperature. Glia culture inserts were removed momentarily from the six-well plates and 200 μl Lipofectamine/DNA mixture was added to each coverslip, followed by return of the glia culture inserts to each well. Neurons were incubated with the Lipofectamine/DNA mixture at 37°C for 2–4 h. After the 2–4 h incubation period, glia inserts were removed again and the media containing the Lipofectamine/DNA mixture was removed, Glia culture inserts were returned to each well, and the glia media from each insert was changed. Neurons were cultured for an additional 24–72 h prior to imaging.

### D2R immunocytochemistry

Striatal neurons were transfected with pH-DRD2 for 48 h as previously described (Li et al., 2012a), washed with ACSF, and incubated with rabbit polyclonal anti-GFP antibody (1:200 dilution; a gift from Dr. Richard L. Huganir, Department of Neuroscience, Johns Hopkins University School of Medicine/HHMI) for 1 h at 4°C to stain surface receptors. Neurons were subsequently fixed with Parafix (4% sucrose, 4% paraformaldehyde in PBS, pH 7.4), permeabilized with 0.25% Triton X-100 in PBS, blocked with 10% normal donkey serum in PBS at 37°C for 1 h, and subsequently stained with a mouse monoclonal GFP antibody at a dilution of 1:100 (a gift from Dr. Richard L. Huganir) to stain for total pH-DRD2 to amplify green fluorescent signal from superecliptic pHluorin at neutral pH. Neurons were then washed four times and subsequently stained with Alexa Fluor 647 Red-X donkey anti-Rabbit IgG (H+L; 1:100 dilution; catalog# 647 711-605-152, Jackson Immunoresearch Laboratories, Inc.) and Alexa Fluor 488 donkey anti-mouse IgG (H+L; 1:200 dilution; catalog# 705-545-147, Jackson Immunoresearch Laboratories, Inc.) secondary antibodies. Images were acquired on an inverted Zeiss fluorescent microscope using a 40X objective (NA, 1.30) and a 2.5X relay lens between the microscope and the camera. Fluorescent intensities were quantified using ImageJ (NIH, https://imagej.nih.gov/ij/). The ratio between surface/total of pH-DRD2 signal was normalized to the control group (pH-DRD2 without DA stimulation) as 100%. Compounds were diluted in PBS in the presence of 0.2 μM sodium metabisulfite.

### Total internal reflection fluorescence microscopy (TIRFM)

TIRF imaging was conducted on a custom made in-house system with a 100-mW Cyan laser for excitation, and an Electron Multiplying Charge Coupled Device (EMCCD) camera for a detector. Prior to imaging experiments, a coverslip containing transfected neurons as described above is assembled into a live-imaging chamber and incubated with ACSF. Transfected neurons are identified visually under epifluorescent imaging, and subject to a 1 min photo-bleach to eliminate pre-existing pHluorin fluorescence on the plasma membrane. Following photobleaching, recording is performed for 1 min at 10 Hz with each recording containing 600 images and the gain setting of the electron multiplier set to maximum. Individual insertion events are identified in the recording ImageJ, and analyzed manually.

### Biotinylated D2R internalization assay

Experiments were performed as previously described (Lee et al., 2007). Briefly, HEK293 cells transfected with HA-D2R were washed with cold PBS (137 mM NaCl, 2.7 mM KCl, 10 mM Na_2_HPO4, 2 mM KH_2_PO4) and incubated in 0.5 mg/ml Sulfo-NHS-SS-biotin (Pierce/Thermo Fisher, Inc.) in PBS at 4°C for 30 min. Cells were washed with PBS to remove and quench residual biotinylation reagent and then placed in culture medium for 30 min with either vehicle (control), quinpirole, or MLS1547 (dissolved in buffer plus 0.1% DMSO). Cells were then washed on ice with PBS, and the remaining cell surface biotinylated proteins were stripped twice for 15 min each using 100 mM MESNA (Sigma-Aldrich, Inc.) dissolved in solution containing 50 mM Tris pH 8.8, 100 mM NaCl, 1 mM EDTA, and 0.2% BSA. Stripped cells were then quenched with iodoacetamide buffer (22 mg/ml iodoacetamide in PBS) for 10 min at 4°C. Cells were extracted with lysis buffer [0.5% sodium deoxycholate (DOC), 1% NP40, 150 mM NaCl, 10 mM Tris pH 7.4, 1% Triton X-100, 0.1% SDS] supplemented with protease inhibitor cocktail (Sigma-Aldrich, Inc.). Cell debris was removed by centrifugation at 13,000 g for 15 min at 4°C. Extracts containing equal amounts of cellular protein (determined using BCA protein assay) were incubated with immobilized streptavidin beads on a rotator overnight at 4°C. Protein-bound streptavidin beads were centrifuged at 3,000 g for 1 min at 4°C and washed three times with lysis buffer. Proteins were eluted and denatured in SDS sample buffer and separated by SDS-PAGE. Proteins were transferred to nitrocellulose and biotinylated proteins were detected by immunoblotting with anti-HA (for D2R) (Roche, Inc.) and anti-TfR (for transferrin receptor) (Santa Cruz Biotechnology, Inc.).

### BRET biosensor-based D2R internalization assay

BRET assays for measuring receptor internalization were performed by tagging plasma membrane (PM) anchored Lyn-kinase's acylation moiety with GFP10 (lyn-GFP10) and were performed as described previously (Namkung et al., 2016). Briefly, HEK293SL cells were cultured in DMEM supplemented with 5% fetal bovine serum and 20 μg/ml gentamycin and maintained at 37°C in 5% CO_2_ and 90% humidity. Cells were seeded at ~7.5 × 10^5^ cells per 100 mm dishes and transfected with D2S-RLuc8 and lyn-GFP10 the following day using calcium phosphate (Simaan et al., 2005). After 18 h of transfection, the medium was replaced, and the cells were replated onto poly-ornithine coated white 96-well plates (~25,000 cells/well). The next day, cells were washed once with pre-warmed Tyrode's buffer (140 mM NaCl, 2.7 mM KCl, 1 mM CaCl_2_, 12 mM NaHCO_3_, 5.6 mM D-glucose, 0.5 mM MgCl_2_, 0.37 mM NaH_2_PO_4_, 25 mM HEPES, pH 7.4), and then stimulated with various concentrations of ligands in Tyrode's buffer in the presence of 0.2 mM sodium metabisulfite for 30 min at 37°C. The cell-permeable substrate, coelenterazine 400a was added at a final concentration of 5 μM in Tyrode's buffer 3–4 min before BRET measurements. Measurements were performed by using Synergy2 (BioTek®) microplate reader with a filter set of 410/80 and 515/30 nm for detecting the Rluc8 *renilla* luciferase (donor) and GFP10 (acceptor) light emissions, respectively. The BRET signal was determined by calculating the ratio of the light intensity emitted by the GFP10 over the light intensity emitted by the Rluc8. Experiments were performed in triplicate and the data expressed as mean ± SEM of the BRET ratio.

### Chemistry

The synthesis of new analogs is described in the Supplemental Information.

## Results

### Agonist-induced receptor internalization studies

MLS1547 has previously been characterized as a G protein-biased agonist at the D2R, displaying high efficacy in stimulating G protein-mediated signaling, but no observable efficacy in recruiting β-arrestin-2, as detected using several cell-based assays (Free et al., 2014). As agonist-induced D2R internalization has been shown to be mediated by β-arrestin-2 (also referred to as arrestin-3) (Skinbjerg et al., 2009), we sought to evaluate the effects of MLS1547 on D2R internalization using several different methods.

**Figure 1:**
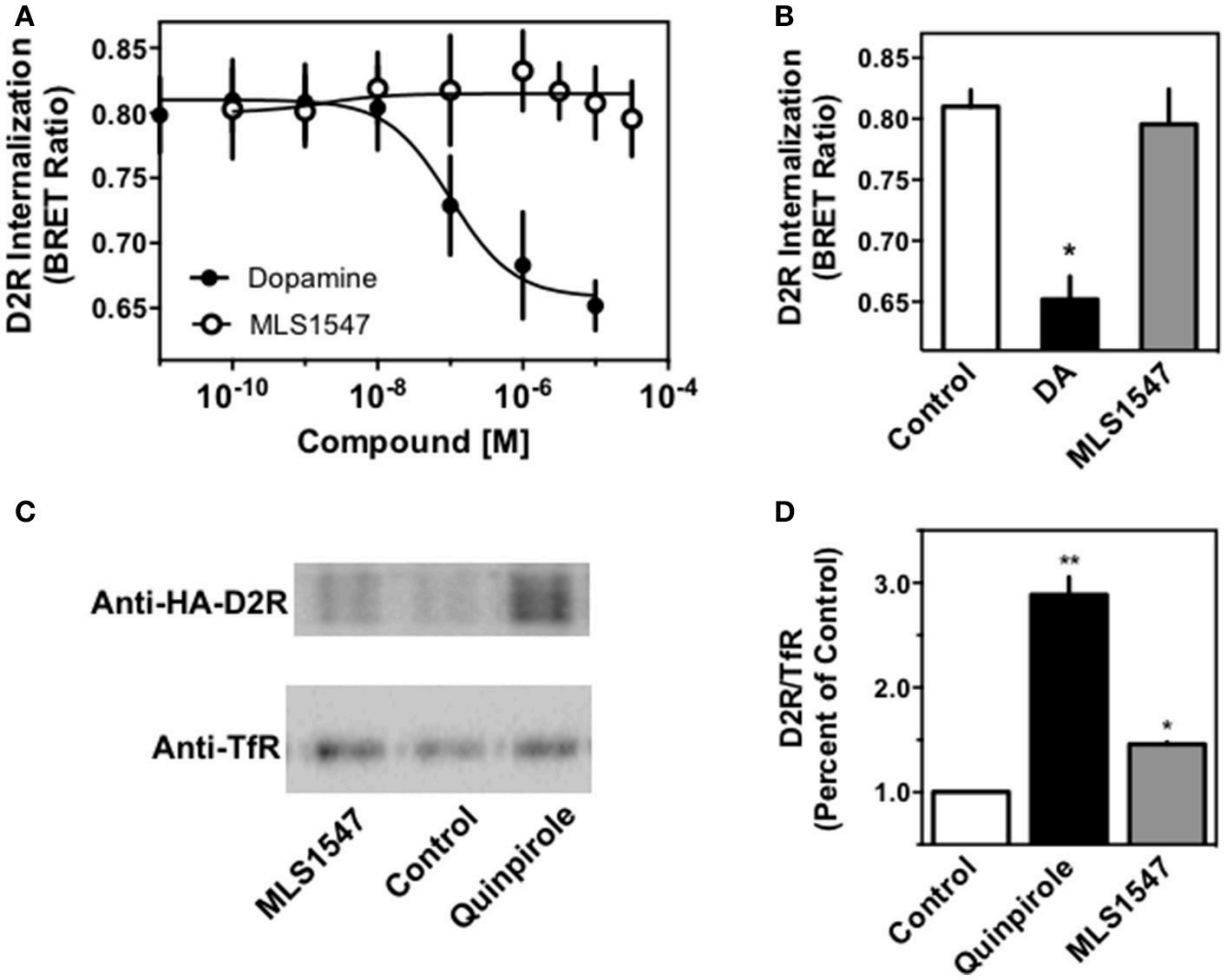
Investigation of D2R internalization in non-neuronal cell systems. **(A)** HEK293 cells were transiently transfected with D2R-Rluc8 and a modified GFP10, which together produce constitutive bystander BRET. On the day of assay, the cells were stimulated with either dopamine (DA) or MLS1547 for 30 min at 37°C before BRET measurement. The data are graphed as mean dose-response curves from three independent experiments. DA exhibited an logEC50 of −7.00 ± 0.36 (EC50, 0.1 μM) for BRET reduction/D2R internalization, while MLS1547 treatment failed to alter the BRET signal. **(B)** Maximum BRET signal obtained after treatment with vehicle (control, 0.81 ± 0.02), 10 μM DA (0.65 ± 0.02), or 30 μM MLS1547 (0.79 ± 0.03). All data are expressed as mean ± SEM of the BRET ratio from three independent experiments. ^*^P < 0.05, unpaired Student's *t*-test compared to untreated controls. **(C)** HA-tagged D2R was transiently transfected into HEK293 cells and surface D2R was biotinylated as described in Methods. After washing, cells were treated with either vehicle (control), 10 μM quinpirole, or 10 μM MLS1547 or for 30 min at 37°C. Biotin was cleaved from D2R that remained on the cell surface, followed by cell lysis and isolation of biotinylated (internalized) D2R using avidin beads. Western blotting was used to detect D2R and also transferrin receptor (TfR) (as a gel loading control). A single experiment, representative of three, is shown (the original full blot is displayed in the Supplemental Materials). **(D)** Densitometric analyses were performed and the data expressed as D2R/TfR ratios, relative to the untreated controls (1.0). Treatment values are provided as mean ± S.E.M: quinpirole = 2.8 ± 0.2 and MLS1547 = 1.5 ± 0.03. One-way ANOVA followed by Bonferroni's post-test was performed: ^*^p < 0.05, and ^**^p < 0.01.

In a first approach, we used a BRET-based biosensor that employs a modified GFP protein, which is cell surface-localized (Namkung et al., 2016) and see Methods. Co-transfection of D2R-Rluc8 and the cell surface-localized GFP-tagged protein in HEK293 cells results in constitutive BRET when they are both expressed at the cell surface and in close proximity. Upon agonist stimulation, the receptor is internalized but the GFP-tagged protein remains in the plasma membrane resulting in a decrease in the BRET ratio due to separation of the BRET donor (D2R-Rluc8) and acceptor (GFP) (Namkung et al., 2016). As shown in Figure 1A, stimulation with DA resulted in a dose-dependent decrease in the BRET ratio. When the cells were stimulated with MLS1547, they failed to elicit a significant change in BRET ratio at a concentration up to 30 μM, which results in full activation of G protein signaling (Free et al., 2014 and see below) and fully occupies the D2R as shown using radioligand binding assays (Free et al., 2014). Figure 1B shows single concentration data using 10 μM of DA or 30 μM of MLS1547. DA treatment promoted significant receptor internalization, whereas MLS1547 treatment did not significantly change the BRET signal. These initial findings suggest that MLS1547 is relatively ineffective in D2R internalization when compared to DA.

We next took a different approach to assess agonist-induced internalization using a cell surface biotinylation assay and transient expression of an HA-tagged D2R in HEK293 cells. In this method, the D2R is labeled with biotin at the cell surface followed by agonist treatment of the cells. Biotin is then cleaved from cell surface proteins (internalized receptors are protected from cleavage) followed by lysis and isolation of the biotinylated proteins via binding to avidin beads (Lee et al., 2007). Figure 1C shows a Western blot of internalized D2R protein after treatment with 10 μM of MLS1547 or 10 μM of the D2R agonist quinpirole. The D2R is visualized using an antibody directed to an HA tag on the receptor (top blot). Visualization of the transferrin receptor (TfR) was also performed as a gel loading control (bottom blot). The blots were quantified using densitometric analyses and graphed as a percentage of D2R protein divided by the loading control (TfR), with untreated control levels being set to 1.0 (Figure 1D). Notably, treatment with the D2R agonist quinpirole resulted in a robust loss of cell surface D2R protein (Figure 1D). MLS1547 treatment also resulted in statistically significant receptor internalization, however, this response was only a fraction of that produced by quinpirole (Figure 1D). Thus, these results differed somewhat from those obtained using the BRET assay (Figures 1A,B) in that MLS1547 promoted a small, but detectable amount of receptor internalization.

**Figure 2:**
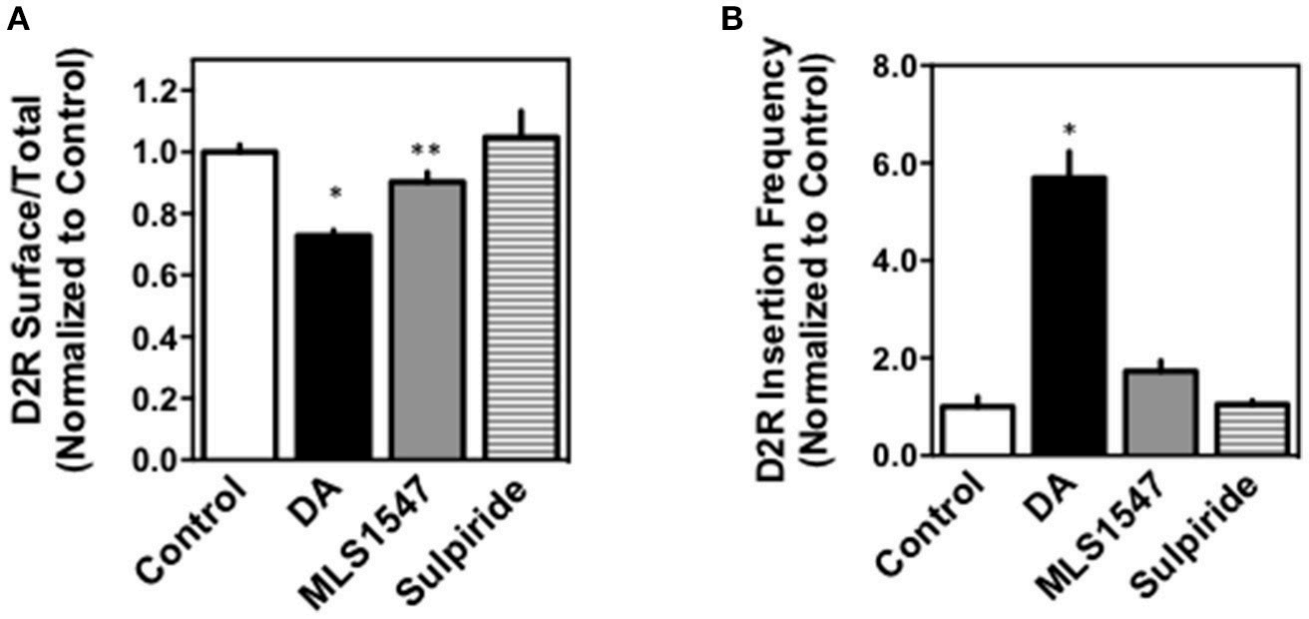
Investigation of D2R internalization in neuronal cell systems. **(A)** Striatal neurons were prepared and transfected with the D2R-pHluorin construct (pH-DRD2) as described in Methods. 48 h later, the neurons were treated with vehicle (control) or 10 μM dopamine (DA), 30 μM MLS1547, or 30 μM sulpiride for 45 min. Neurons were washed and then stained with a polyclonal GFP antibody to detect surface D2R followed by fixation, permeabilization and re-staining with a monoclonal GFP antibody to detect total cellular D2R. The ratios between surface to total pH-DRD2 signal were normalized to the control group in each experiment and are expressed as mean ± S.E.M of the indicated number of cells (*n*): control = 1.00 ± 0.02, *n* = 746; DA = 0.72 ± 0.02, *n* = 627; MLS1547 = 0.90 ± 0.03, *n* = 812, sulpiride = 1.05 ± 0.08, *n* = 171. One-way ANOVA followed by Bonferroni's post-test was performed: ^*^p < 0.0001 and ^**^p < 0.0262. **(B)** Striatal neurons expressing the D2R-pHluorin construct were exposed to vehicle (control) or 10 μM DA, 30 μM MLS1547, or 30 μM sulpiride for 20 min and the number of reinsertion events/(min × μm^2^) were visualized in real time as described in Methods. The average reinsertion frequencies for the drug treatments were normalized to the control group in each experiment and are expressed as mean ± S.E.M of the indicated number of cells (*n*): control = 1.00 ± 0.20, *n* = 51; DA = 5.70 ± 0.54, *n* = 49; MLS1547 = 1.73 ± 0.22, *n* = 50, sulpiride = 1.56 ± 0.34, *n* = 15. One-way ANOVA followed by Bonferroni's post-test was performed, ^*^p < 0.0001.

As the above experiments used non-neuronal cells (HEK293) to examine D2R internalization, we wished to examine this response within a neuronal context. Thus, we performed studies to examine D2R internalization and recycling in striatal neurons from E15-E18 mouse embryos using both immunocytochemistry (ICC) and total internal refection fluorescent (TIRF) microscopy (Li et al., 2012a,b; Daly et al., 2015). Figure 2A shows quantification of ICC assays following treatment of the transfected cells with either 10 μM DA, 30 μM MLS1547, 30 μM of the D2R antagonist sulpiride, or vehicle (control). Data are expressed as the ratio of surface to total receptors normalized to the control. Notably, DA treatment resulted in a significant decrease in D2R surface expression when compared to the control. Interestingly, MLS1547 promoted a low, but statistically significant, degree of receptor internalization when compared to the control, however, this response was much less than that observed with DA. The D2R antagonist sulpiride was used as a negative control and, as expected, no significant D2R internalization was detected after sulpiride treatment.

For TIRF microscopy studies, the neurons were transfected with a D2R construct tagged with a pH-dependent GFP (pHluorin) that loses fluorescence in the acidic environment of internalized vesicles (Li et al., 2012a,b; Daly et al., 2015). This enables the visualization of vesicle exocytosis or “reinsertion,” as pHluorin fluorescence is regained the moment the vesicle fuses with the cell membrane. It has been shown that increased receptor internalization correlates to an increase in reinsertion frequency, which is theorized to be due to recycling of D2Rs back to the cell membrane following agonist-stimulated endocytosis (Li et al., 2012a,b). The frequency of D2R-containing vesicle reinsertion was measured via real-time TIRF microscopy by photobleaching the neurons and then treating them for 20 min with either 10 μM DA, 30 μM MLS1547, 30 μM sulpiride, or vehicle (control) (Figure 2B). DA treatment caused a significant increase in reinsertion frequency when compared to the control correlating to an increase in receptor internalization. In contrast, MLS1547 treatment did not result in a significant increase in reinsertion frequency when compared to control. As observed with the ICC experiments, sulpiride had no effect on D2R internalization. Taken together, these data show that MLS1547 is relatively ineffective in promoting D2R internalization in striatal neurons.

### Structural analogs of MLS1547

**Figure 3:**
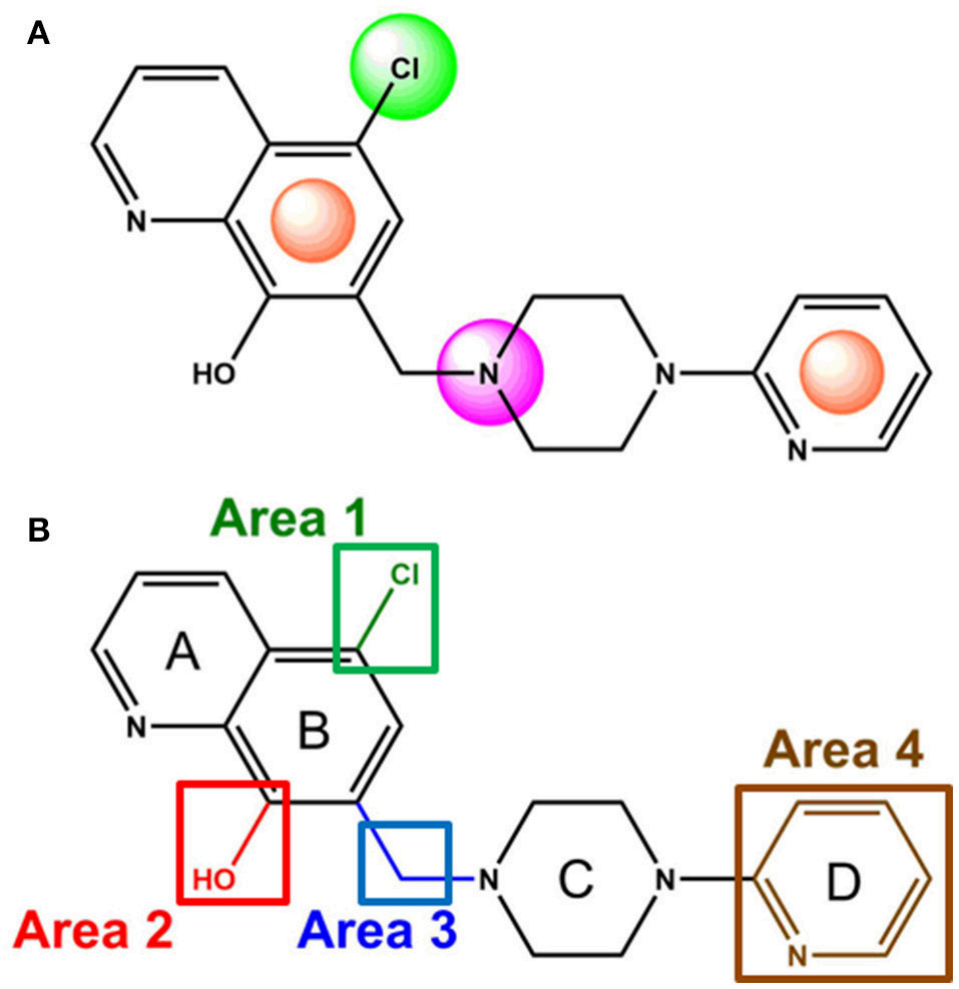
Structure of MLS1547. **(A)** Pharmacophore model depicting four required features for agonist activity and G protein bias. Green represents a hydrophobic component, orange represents two aromatic components, and purple represents a positively charged component. **(B)** The four main areas of the scaffold that were modified are indicated.

**Scheme 1:**
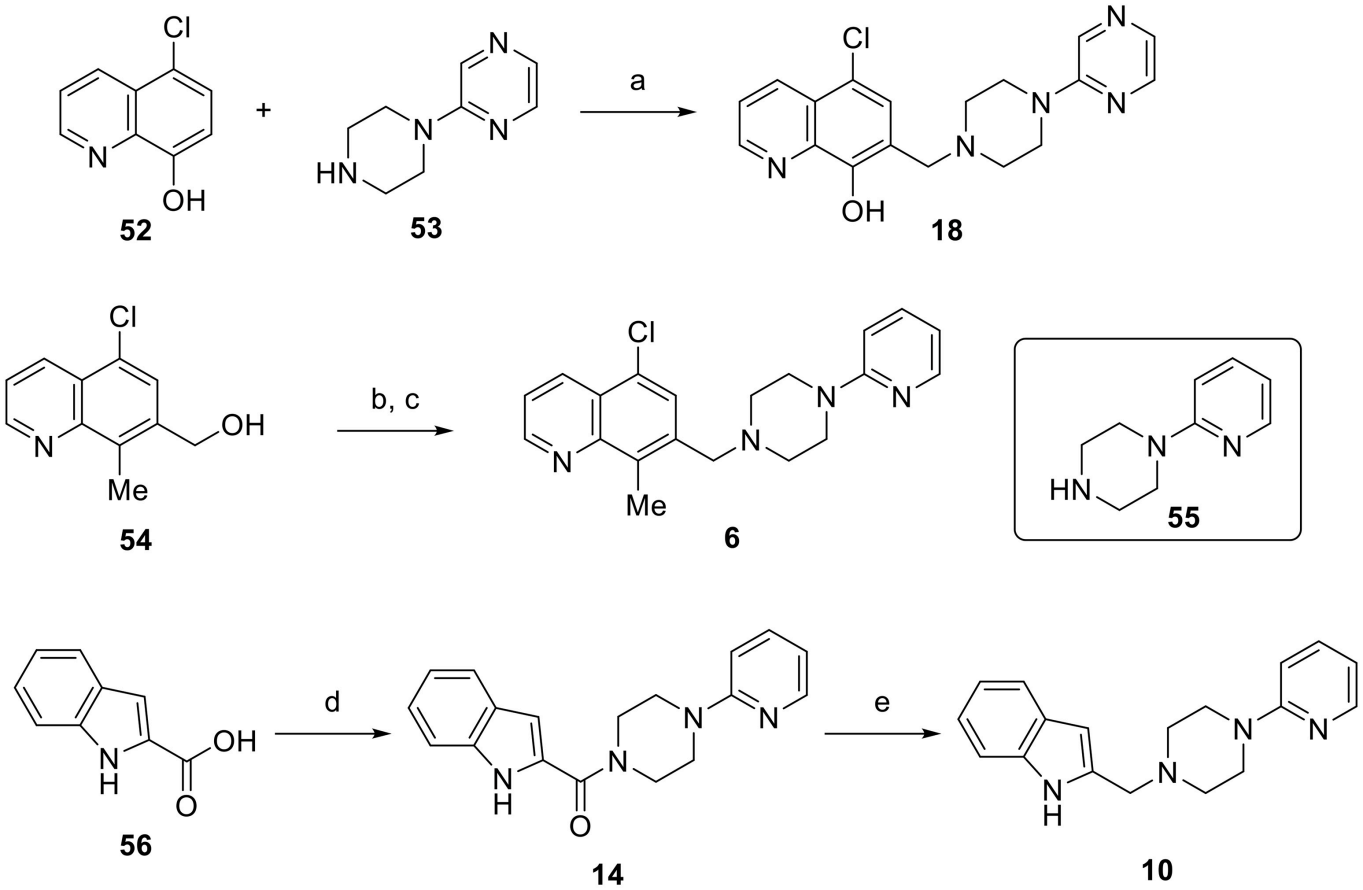
Representative synthetic sequences for SAR analogs **1-51**. Reagents and conditions: (a) pyridine, formaldehyde (37 wt% in water), 55% yield; (b) MsCl, Et_3_N, CH_2_Cl_2_; (c) **55**, Et_3_N, DMF, 56% yield (two steps); (d) **55**, HATU, DIEA, DMF, 34% yield; (e) LiAlH_4_, THF, 42% yield.

In order to better understand the SAR for the biased signaling of MLS1547, as well as to develop a more optimized compound, an iterative chemical synthesis of additional analogs was conducted. Figure 3A illustrates the parental compound **1** and the previously developed pharmacophore model highlighting four regions of the molecule that are believed to be important for G protein bias. These include a hydrophobic binding moiety, two aromatic groups, and a positively charged feature (Free et al., 2014). Keeping this model in mind, Figure 3B shows the four main areas of the molecule that we selected for derivatization to further investigate the SAR for this scaffold. The target analogs **2–51** were readily prepared via one of the three synthetic sequences illustrated in Scheme 1, each advantageous for a range of structural analogs.

For 8-hydroxyquinoline substrates, a Mannich-type reaction (Blicke, 1942) was employed to directly combine quinoline fragment such as **52**, formaldehyde and a piperazine fragment such as **53** with simultaneous incorporation of the methylene linker. Nonphenolic quinolines required a two-step sequence to utilize benzylic alcohols such as **54** as coupling partners with piperazine fragments (e.g., **55**) to prepare the final analogs. Replacement of the quinoline with alternative heterocycles was achieved via coupling of the piperazine fragment (e.g., **55**) with carboxylic acids such as **56** and subsequent amide reduction. This third sequence also provided the intermediate carboxamide compounds (e.g., **14**) for evaluation as D2R agonists. The agonist activities for all of the analogs using both G protein activation (cAMP inhibition) and β-arrestin recruitment assays are summarized in Tables 1–**3** below.

**Table 1:** Modification and replacement of the hydroxyquinoline group (R^1^).

**Entry/cmpd**	** 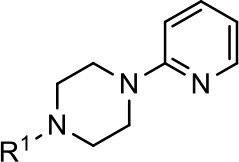 ** **Structure (R^1^ =)**	**cAMP inhibition assay**		**β-arrestin recruitment assay**	
		**EC_50_ (μM ± SEM)**	**E_max_ (% DA control ± SEM)**	**EC_50_ (μM ± SEM)**	**E_max_ (% DA control ± SEM)**
1 (MLS1547)	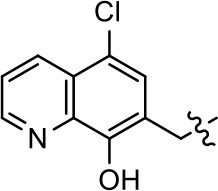	2.2 ± 0.6	95.4 ± 8.9	NA	NA
2	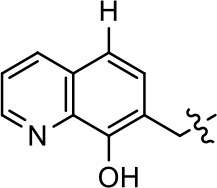	0.3 ± 0.09	97.1 ± 0.9	0.9 ± 0.3	58 ± 8.4
3	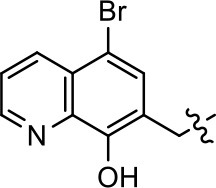	7.9 ± 2.1	75.0 ± 3.9	NA	NA
4	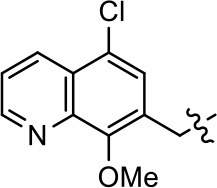	13.4 ± 11.7	37.9 ± 8.1	NA	NA
5	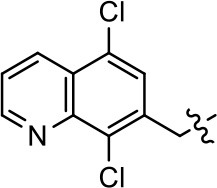	NA	NA	NA	NA
6	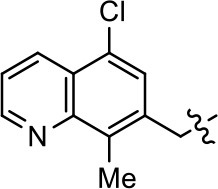	NA	NA	NA	NA
7	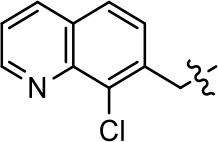	6.6 ± 5.5	56.8 ± 8.3	7.3 ± 1.8	30.1 ± 1.1
8	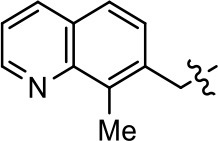	1.4 ± 0.7	48.9 ± 5.4	9.3 ± 2.9	22 ± 1.6
9	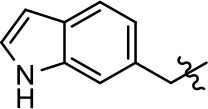	1.6 ± 1.3	51.9 ± 13.8	9.3 ± 6.1	60.8 ± 6.0
10	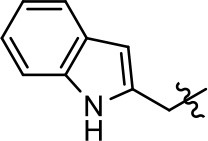	0.2 ± 0.1	75.4 ± 7.6	0.7 ± 0.1	60.9 ± 6.4
11	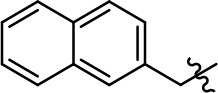	0.1 ± 0.07	93.1 ± 1.6	0.9 ± 0.6	63.3 ± 8.9
12	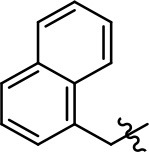	0.7 ± 0.7	89.5 ± 5.2	NA	NA
13	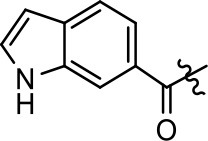	NA	NA	NA	NA
14	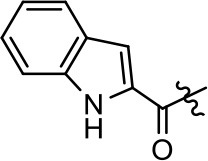	NA	NA	NA	NA

*The compounds were assayed in agonist mode using the DiscoverX cAMP and β-arrestin assays as described in the Materials and Methods. Data are expressed a percentage of the maximum response to dopamine, which was assessed in each experiment. Data represent the means ± SEM from at least three separate experiments. NA, not active*.

### Structure-activity relationships for MLS1547

**Figure 4:**
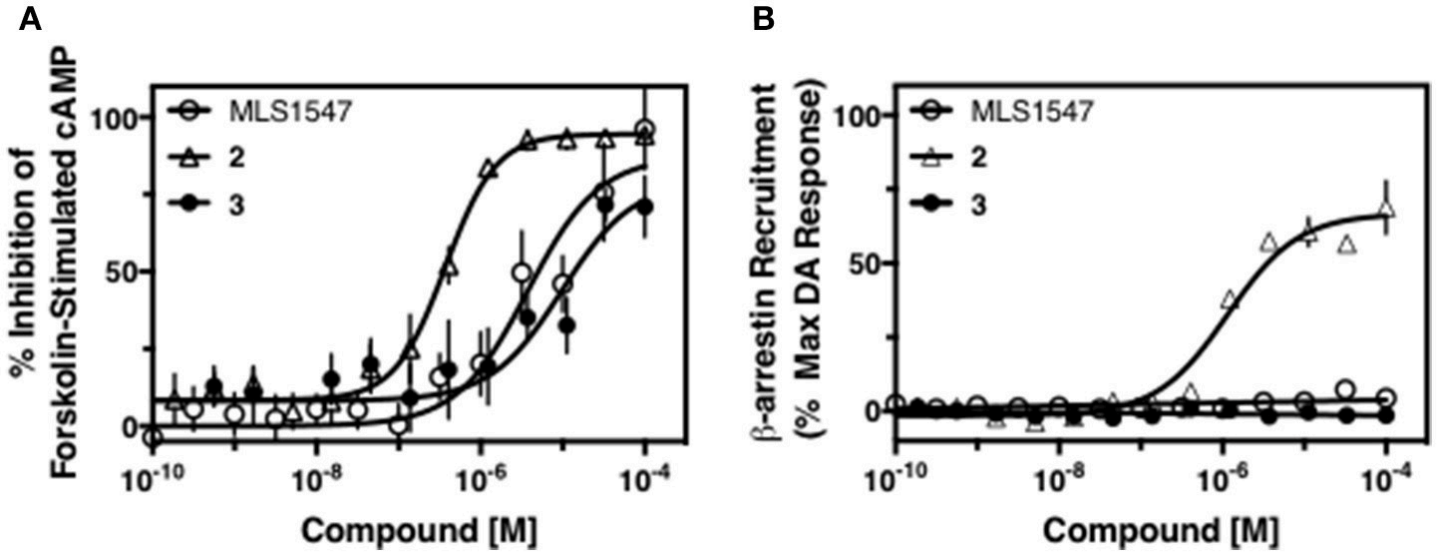
A halogen moiety in area 1 on ring B of MLS1547 enables G protein-bias. CHO cells stably expressing the D2R were assayed for agonist-mediated inhibition of forskolin-stimulated cAMP accumulation **(A)** or recruitment of β-arrestin to the D2R **(B)**, as described in the Materials and Methods. Representative experiments are shown with averaged curve parameters derived from at least three separate experiments displayed in Table 1. Data are expressed as a percentage of the maximum response to dopamine, which was assessed in each experiment.

We previously postulated that a hydrophobic binding pocket comprised of D2R residues I184, F189, and V190 is required for the biased signaling activity of MLS1547 (Free et al., 2014). The chlorine group (area 1) on ring B of MLS1547 (Figure 3B) was hypothesized to interact with this pocket and prevent the tilting of TM5 during receptor activation. With this in mind, our first approach was to further investigate the influence of this hydrophobic binding moiety on compound bias by constructing multiple analogs that vary in this region of the molecule. It can be seen that removing the chlorine group (Tables 1, 2) resulted in a gain in β-arrestin recruitment activity (loss of signaling bias), whereas replacing the chlorine group with a bromine (Tables 1, 3) maintained G protein bias, but resulted in a modest decrease in agonist potency (Figure 4). Similar replacement of Cl with H in MLS1547 analogs (Table 1, **7** and **8**, or Table 3, **39** and **42**) lead to less or non-biased analogs supporting the need for a hydrophobic moiety at this position to maintain G protein signaling bias.

**Table 2:** Modification and replacement of the 2-pyridyl moiety (R^2^).

**Entry/cmpd**	** 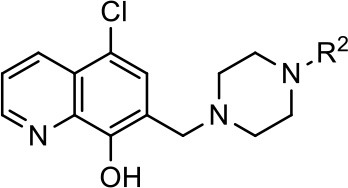 Structure (R^2^ =)**	**cAMP inhibition assay**		**β-arrestin recruitment assay**	
		**EC_50_ (μM ± S.E.M.)**	**E_max_ (% DA control ± S.E.M.)**	**EC_50_ (μM ± S.E.M.)**	**E_max_ (% DA control ± S.E.M.)**
1 (MLS1547)	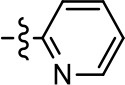	2.2 ± 0.6	95.4 ± 8.9	NA	NA
15	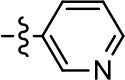	NA	NA	NA	NA
16	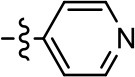	NA	NA	NA	NA
17	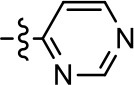	NA	NA	NA	NA
18	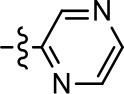	3.5 ± 3.4	83.8 ± 8.5	NA	NA
19	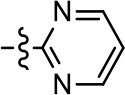	0.7 ± 0.2	92.8 ± 2.1	0.5 ± 0.3	21.6 ± 5.7
20	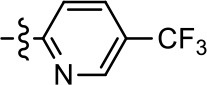	NA	NA	NA	NA
21	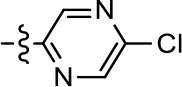	NA	NA	NA	NA
22	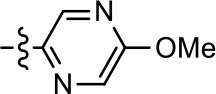	NA	NA	NA	NA
23	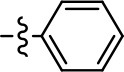	2.8 ± 1.2	66.8 ± 3.9	NA	NA
24	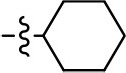	NA	NA	NA	NA

*The compounds were assayed in agonist mode using the DiscoverX cAMP and β-arrestin assays as described in the Materials and Methods. Data are expressed a percentage of the maximum response to dopamine, which was assessed in each experiment. Data represent the means ± SEM from at least three separate experiments. NA, not active*.

**Table 3:** Modification or replacement of both the hydroxyquinoline group (R^1^) and 2-pyridyl moiety (R^2^).

**Entry/cmpd**	** 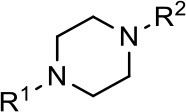 Structure (R^1^ =)**		**cAMP inhibition assay**		**β-arrestin recruitment assay**	
	**(R^1^ =)**	**(R^2^ =)**	**EC_50_ (μM ± S.E.M.)**	**E_max_ (% DA control ± S.E.M.)**	**EC_50_ (μM ± S.E.M.)**	**E_max_ (% DA control ± S.E.M.)**
1 (MLS1547)	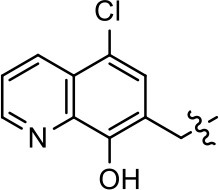	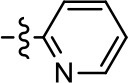	2.2 ± 0.6	95.4 ± 8.9	NA	NA
25	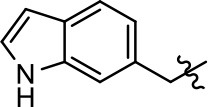		NA	NA	>39	NA
26	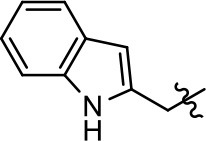		NA	NA	NA	NA
27	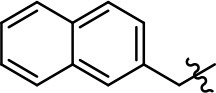	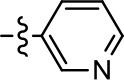	NA	NA	NA	NA
28	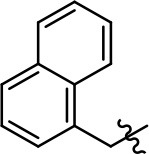		13.3 ± 8.6	70.0 ± 10.0	7.7 ± 0.3	34.9 ± 4.1
29	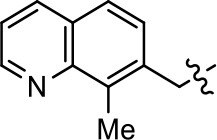	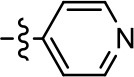	NA	NA	NA	NA
30	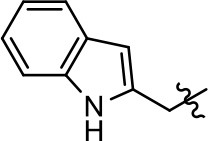		NA	NA	NA	NA
31	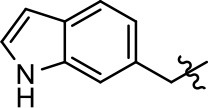	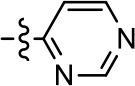	NA	NA	NA	NA
32	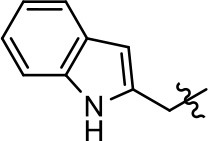		NA	NA	NA	NA
33	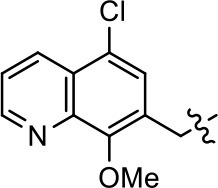		NA	NA	NA	NA
34	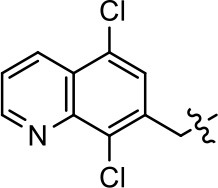		NA	NA	NA	NA
35	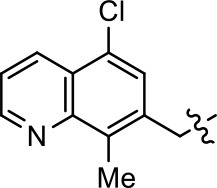	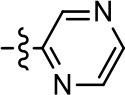	NA	NA	NA	NA
36	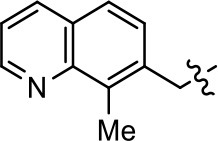		NA	NA	NA	NA
37	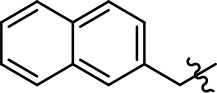	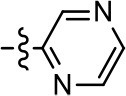	NA	NA	24.8 ± 15.5	54.7 ± 5.1
38	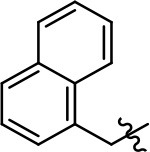		27.3 ± 18.2	66.5 ± 20.6	NA	NA
39	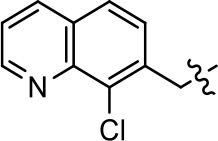	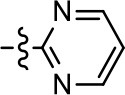	1.2 ± 0.4	59.3 ± 4.9	15.6 ± 8.8	53.4 ± 7.8
40	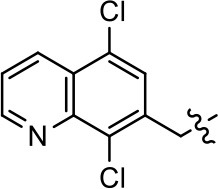		NA	NA	NA	NA
41	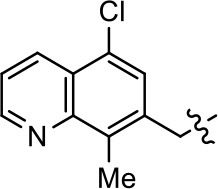		NA	NA	NA	NA
42	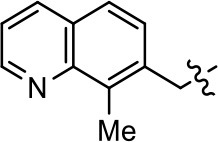		0.8 ± 0.4	58.7 ± 2.8	7.6 ± 1	46.5 ± 1.5
43	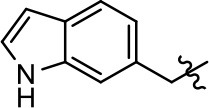		0.04 ± 0.01	58.2 ± 1.4	0.1 ± 0.01	75.1 ± 7.1
44	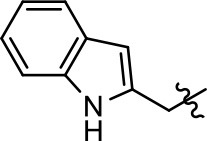		0.6 ± 0.5	44.4 ± 1.7	1.0 ± 0.2	61.4 ± 11.7
45	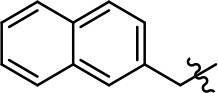		0.8 ± 0.1	51.6 ± 3.5	2.6 ± 1.1	70.6 ± 8.9
46	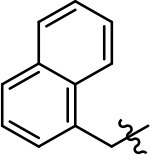		8.5 ± 4.0	90.1 ± 12.7	14.5 ± 1.5	85.6 ± 4.5
47	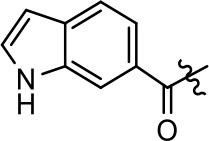		NA	NA	NA	NA
48	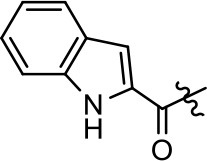		NA	NA	NA	NA
49	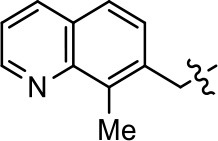		NA	NA	NA	NA
50	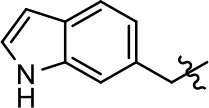	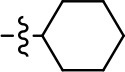	NA	NA	NA	NA
51	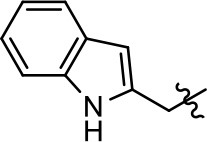		NA	NA	NA	NA

*The compounds were assayed in agonist mode using the DiscoverX cAMP and β-arrestin assays as described in the Materials and Methods. Data are expressed a percentage of the maximum response to dopamine, which was assessed in each experiment. Data represent the means ± SEM from at least three separate experiments. NA, not active*.

**Figure 5:**
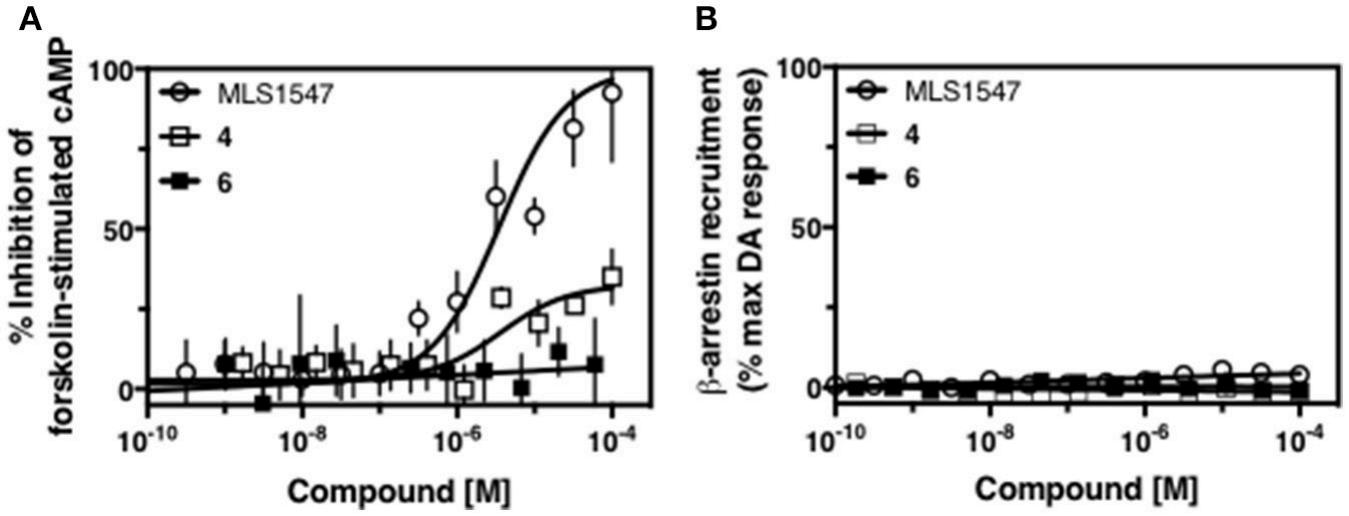
A hydroxyl group in area 2 on ring B of MLS1547 is required for both potency and efficacy of G protein signaling. CHO cells stably expressing the D2R were assayed for agonist-mediated inhibition of forskolin-stimulated cAMP accumulation **(A)** or recruitment of β-arrestin to the D2R **(B)**, as described in the Materials and Methods. Representative experiments are shown with averaged curve parameters derived from at least three separate experiments displayed in Table 1. Data are expressed as a percentage of the maximum response to dopamine, which was assessed in each experiment.

**Figure 6:**
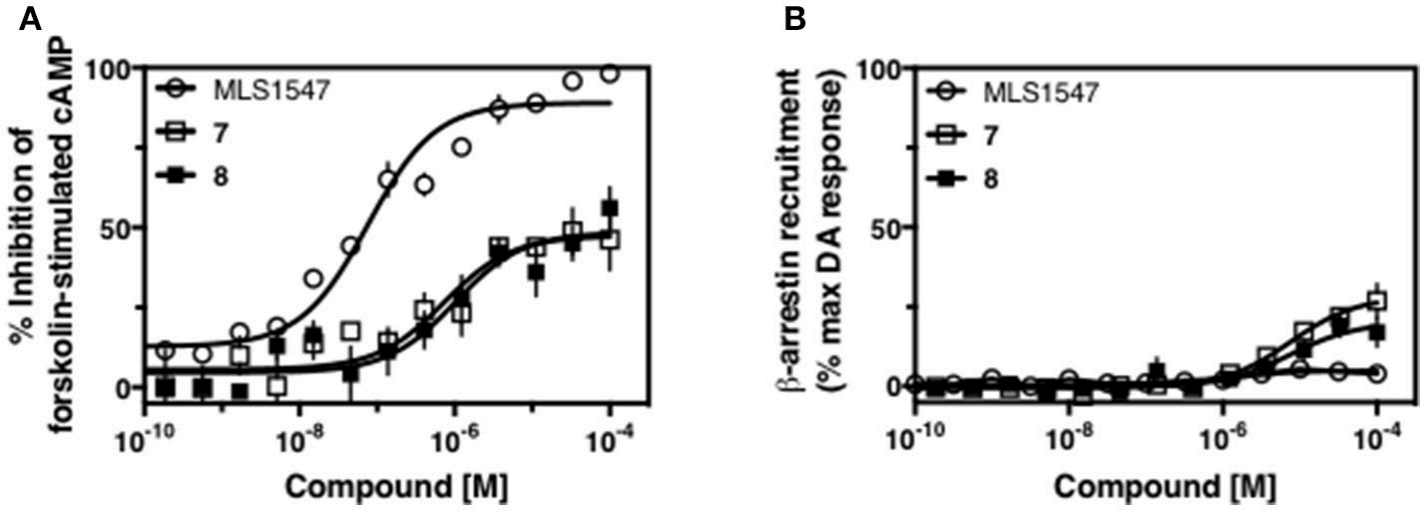
Effects of combined modifications of areas 1 and 2 of ring B of MLS1547 on signaling bias. CHO cells stably expressing the D2R were assayed for agonist-mediated inhibition of forskolin-stimulated cAMP accumulation **(A)** or recruitment of β-arrestin to the D2R **(B)**, as described in the Materials and Methods. Representative experiments are shown with averaged curve parameters derived from at least three separate experiments displayed in Table 1. Data are expressed as a percentage of the maximum response to dopamine, which was assessed in each experiment.

Modification or replacement of the phenol on the chlorine-substituted quinoline ring resulted in a decrease in potency or loss of efficacy of the compound (Table 1, **4–6**), suggesting an important interaction between this hydroxyl group and the D2R (Figure 5). Replacement of the phenol with chlorine or methyl combined with removal of the Area 1 (Figure 3B) chlorine group (Table 1, **7** and **8**) resulted in less biased analogs with agonist efficacy in both the G protein and β-arrestin assays (Figure 6). The reduced steric bulk of these analogs could allow for a change in binding pose that might compensate for the loss of the phenol interaction. These data suggest that the hydroxyl group on ring B (Figure 3B) is a strong driver of both potency and efficacy in this series of compounds and plays a vital role in driving G protein bias and activity.

**Figure 7:**
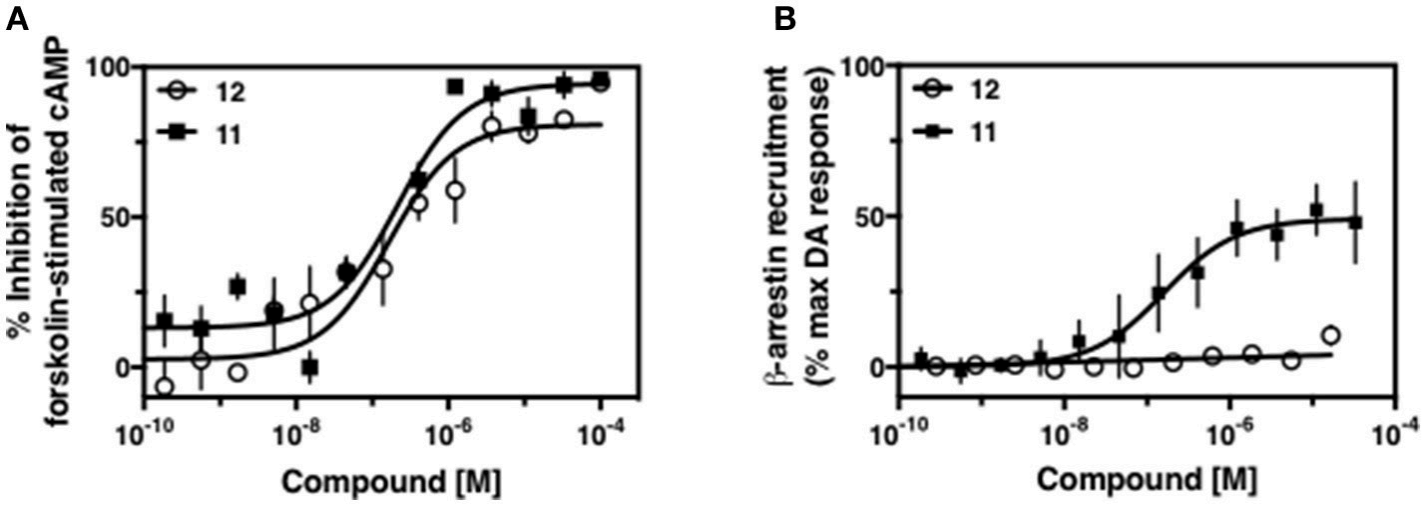
A hydrophobic moiety in the correct orientation is integral to G protein biased signaling activity of the MLS1547 scaffold. CHO cells stably expressing the D2R were assayed for agonist-mediated inhibition of forskolin-stimulated cAMP accumulation **(A)** or recruitment of β-arrestin to the D2R **(B)**, as described in the Materials and Methods. Representative experiments are shown with averaged curve parameters derived from at least three separate experiments displayed in Table 1. Data are expressed as a percentage of the maximum response to dopamine, which was assessed in each experiment.

Replacement of the quinoline ring with other bicyclic aromatic moieties primarily resulted in less biased analogs (Table 1, **9–11**). An interesting exception is the 1-naphthalene group (Table 1, **12**) that was both potent and G protein-biased. We previously found that a naphthalene group can provide the necessary hydrophobic feature for G protein bias, but only if orientated correctly such that it can engage the hydrophobic pocket in the D2R (Free et al., 2014). For instance, **12** is highly G protein-biased whereas **11** is much less biased (Table 1, Figure 7). Compounds with substituted indole moieties in a similar orientation as the naphthalene group of the non-biased compound **11**, (Tables 1, **9** and **10**), resulted in less or non-biased signaling activity (Table 1). Other MLS1547 derivatives (Table 3, **37** and **38**) with naphthalene groups in the two different orientations exhibited a similar phenomenon. For instance, **38** was G protein-biased whereas **37** actually appeared to exhibit β-arrestin bias, albeit with low potency. Similarly, **28** was G protein-biased, whereas **27** lost all activity (Table 3). In aggregate, these data confirm and strengthen our previously proposed pharmacophore model for biased G protein signaling of the MLS1547 scaffold. Specifically, that a hydrophobic moiety is integral to G protein-biased signaling activity at the D2R, and this interaction can be provided either through a halogen group, or a hydrophobic ring in the correct orientation, that interacts with a hydrophobic pocket on the D2R.

We next performed a preliminary investigation of the methylene linker (Area 3, Figure 3B) between the bicyclic aromatic moieties (rings A and B, Figure 3B) and the piperazine moiety (ring C, Figure 3B) by exchanging it for a carbonyl group to create carboxamide derivatives. This was performed within the context of four of the highest potency compounds, **9**, **10**, **43**, and **44** (Tables 1, 3). The corresponding carboxamide derivatives **13**, **14**, **47**, and **48** lost all activity in the functional assays (Tables 1, 3). This likely results from a decrease in the basicity of the adjacent nitrogen, affecting its ability to become protonated at physiological pH. As previously noted, a positively charged nitrogen at this position was described in the pharmacophore model (Figure 3A), which likely forms critical interactions with a conserved aspartate residue (Asp^3.32^) within TM3 of the receptor (Michino et al., 2015). However, it should be noted that the introduction of a carbonyl group in the linker also greatly increases the rigidity the scaffold, which might be deleterious for its interaction with the D2R.

Based on previously published SAR and the pharmacophore model, the pyridine moiety of MLS1547 (Figure 3B, area 4) is believed to confer increased agonist potency, likely through formation of a hydrogen bond to T412 of the D2R (Free et al., 2014). To further dissect the influence of the pyridine ring, we initially replaced it with a cyclohexane group within the context of both G protein-biased and non-biased scaffolds (Table 2, **24** and Table 3, **49-51**). Notably, these analogs lost all agonist activity at both G protein and β-arrestin-mediated signaling. The agonist potency and G protein selectivity of MLS1547 was restored in the analog where the 2-pyridyl was replaced with phenyl (Table 2, **23**). These data confirm the notion (Free et al., 2014) that the aromaticity of ring D is essential for agonist efficacy. Notably, all of the compounds with cyclohexane substituents retained binding affinity for the D2R as evidenced by their ability to antagonize both G protein and β-arrestin recruitment activities (data not shown).

To further investigate the role of the pyridine moiety in MLS1547 activity, we modified the location of the nitrogen atom within the ring. Moving the nitrogen to either the meta- or para- positions results in a complete loss of agonist activity (e.g., Table 2, **15** and **16**, and Table 3, **25–27** and **29–30**). One notable exception is **28** (Table 3), which is a low potency partial agonist with higher efficacy for G protein signaling. Substitution of the 2-pyridyl group at the para-position also appears to be detrimental to potency, as the trifluoromethyl analog (Table 2, **20**) lost all agonist activity at the D2R. Overall, these data illustrate the importance of a nitrogen at the ortho-position of ring D (Figure 3B) in order for the compound to exert agonist activity at the D2R and suggests that critical positioning occurs between the ring D and D2R residues.

**Figure 8:**
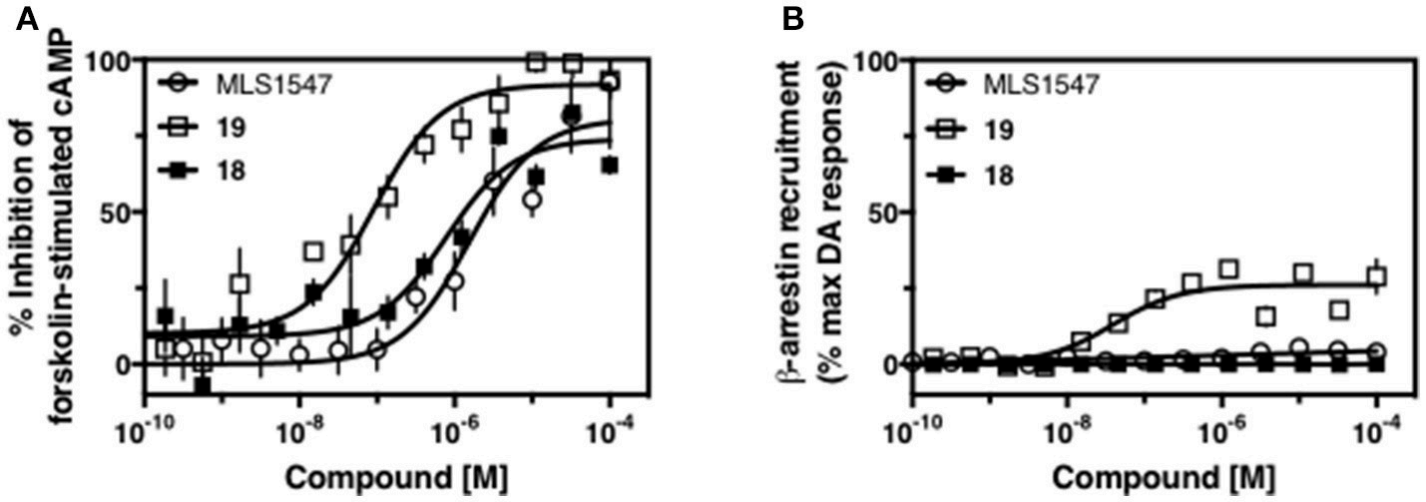
The addition of a second nitrogen at the 6-position of ring D increases agonist potency, but decreases G protein signaling bias. CHO cells stably expressing the D2R were assayed for agonist-mediated inhibition of forskolin-stimulated cAMP accumulation **(A)** or recruitment of β-arrestin to the D2R **(B)**, as described in the Materials and Methods. Representative experiments are shown with averaged curve parameters derived from at least three separate experiments displayed in Table 3. Data are expressed as a percentage of the maximum response to dopamine, which was assessed in each experiment.

We were next interested in examining the effects of adding a second nitrogen to ring D while maintaining the presence of a nitrogen at the ortho-position. Introduction of a second nitrogen at either the ortho- or meta-positions was well-tolerated with pyrazine **18** (ortho-, meta-disubstitution) affording a G protein-biased analog of comparable potency and efficacy to MLS1547 (Table 2, Figure 8). The addition of a second nitrogen at the ortho-position generally increased potency for G protein-mediated signaling, but also led to efficacy for β-arrestin recruitment (Table 2, **19**, Figure 8, or Table 3, **39** and **42–46**). However, the addition of a second nitrogen to the ortho- or meta-positions of ring D within analogs containing detrimental modifications to the phenol moiety did not re-establish lost agonist activity (Table 3, **33**–**36** and **40**, **41**, **47**, and **48**). Notably, ortho-, para-disubstitution uniformly provided inactive analogs (Table 2, **17** and Table 3, **31**, and **32**), again indicating the sensitivity of the para-position of the D ring. Moreover, para substitution of the pyrazine ring completed ablated agonist activity (Table 2, **21** and **22**), as previously observed for the 2-pyridyl analog **20** (Table 2). Generally, we observed that, while the addition of a second nitrogen at the meta-position increases agonist potency, it results in a less biased compound. Adding a second nitrogen meta to the linker results in somewhat scaffold-dependent effects, whereas a second nitrogen para to the linker causes a loss of agonist activity. These data support the need for a single nitrogen ortho to the linker without any substitution at the para-position to maintain the activity and bias of the parent compound.

## Discussion

We previously identified MLS1547 as a G protein-biased agonist at the D2R using a variety of signaling assays (Free et al., 2014). G protein-mediated assays included D2R-stimulated Ca^2+^ mobilization, via a chimeric Gqi5 protein, and D2R-mediated inhibition of cAMP accumulation via Gi/o proteins; β-arrestin recruitment assays included assessment of D2R-β-arrestin interactions, as measured via BRET, and also the DiscoverX β-galactosidase complementation assay (Free et al., 2014). MLS1547 was characterized as exhibiting high efficacy (relative to dopamine) in the G protein-mediated assays and little efficacy in the β-arrestin recruitment assays (Free et al., 2014). These assays, however, involved different cell types, HEK293 and CHO (both non-neuronal), undoubtedly expressing different types/levels of other proteins that could regulate receptor coupling/function. Recently, it has been noted that the determination of ligand signaling bias can depend on variables such as cell background, receptor reserve, signal amplification and even the kinetics of the assay (Kenakin and Christopoulos, 2013; Klein Herenbrink et al., 2016).

Notably, Chen et al. (2016) have replicated our results showing that MLS1547 is a G protein-biased agonist using cAMP inhibition assays and the DiscoverX β-arrestin recruitment assay. Although, using two other β-arrestin recruitment assays, these investigators found that MLS1547 exhibited some efficacy for β-arrestin recruitment. One of these, the Tango assay, uses a gene reporter amplification output and a 20 h incubation period of test compounds with the cells. This assay output and time format results in significant amplification of agonist responses thus making estimation of relative efficacies difficult. The second assay measured receptor-β-arrestin association via BRET, but notably required transfection and over-expression of GRK2 in order to detect a response. In contrast, using native levels of GRKs, we found that MLS1547 was devoid of this activity using a similar β-arrestin recruitment assay (Free et al., 2014). Given these somewhat disparate results in measuring proximal D2R-β-arrestin association, we were interested in investigating a downstream pathway resulting from β-arrestin recruitment, namely receptor internalization. We have previously shown, using native brain tissue from wild-type and β-arrestin-2 knock-out mice, that β-arrestin-2 is required for D2R internalization (Skinbjerg et al., 2009).

Initially, we were interested in investigating MLS1547-induced D2R internalization in HEK293 cells, a non-neuronal cell type used for many of the β-arrestin recruitment assays mentioned above. Two different approaches were utilized. Firstly, we used a BRET assay that takes advantage of constitutive bystander BRET between Rluc8 tagged D2R (donor) and a cell-surface restricted GFP10 (lyn-GFP10, acceptor) (Namkung et al., 2016). When the D2R is internalized in response to agonist, the BRET signal is decreased due to the separation of the BRET donor (D2R) and acceptor (lyn-GFP10) which remains at the cell surface. In these assays, dopamine was shown to promote robust internalization, however, MLS1547 was without effect. In a second approach, we used a biotinylation assay to detect D2Rs that had undergone internalization in response to agonist treatment. In these assays, MLS1547 was found to promote some detectable receptor internalization, but the efficacy of this response was quite low in comparison to that of a full, unbiased D2R agonist. Our conclusion from these assays is that MLS1547 is nearly devoid of efficacy with respect to promoting D2R internalization in HEK293 cells.

We were next interested in testing D2R internalization using a more physiologically relevant model system and turned to primary striatal neurons in culture. Two different approaches were utilized. Firstly, we used immunocytochemistry techniques and an experimental design that can detect either the total cellular receptor population (surface plus internalized) or just receptors at the cell surface. Using this approach, dopamine treatment was found to promote robust D2R internalization. MLS1547 treatment also promoted a low degree of statistically significant internalization, however, the efficacy for this response was much less than that for dopamine. We also used a completely different approach to assess D2R internalization in the striatal neurons that involved measuring reinsertion of internalized receptors at the cell surface. This approach has previously been validated by Li et al. (2012a,b) for assessing D2R internalization in neuronal cultures. Using this technique, we found that dopamine promoted extensive internalization of the D2R, whereas ML1547 treatment was without effect.

Our overall conclusion from these studies is that the pharmacological properties of MLS1547 delineated in heterologous expression systems were largely recapitulated in native neuronal (striatal) tissue. While G protein-coupling wasn't examined directly, Scarduzio et al. (2017) recently showed that MLS1547 decreases the firing rate of striatal neurons through a D2R-activated Gi/o-mediated pathway indicating that this compound does stimulate G protein-mediated signaling in native striatal neurons. In contrast, Scarduzio et al. (2017) found that the D2R β-arrestin-biased agonist, UNC9994 (Allen et al., 2011; Chen et al., 2012) had no effect on striatal neuron firing rate. (UNC9994 is actually an antagonist of D2R G protein-mediated signaling). The biased signaling properties of UNC9994 may be similarly affected by the cellular milieu in which the D2R is expressed. Urs et al. (2016, 2017) have suggested that this D2R ligand exhibits greater efficacy for recruiting β-arrestin in the cortex vs. the striatum, thus emphasizing the need to evaluate functionally selective ligands within multiple cellular contexts.

A second goal of the present study was to further investigate the structural requirements for the biased signaling properties of the MLS1547 scaffold. In general, we investigated modifications of the scaffold within the four main areas shown in Figure 3. Modifications of rings A and B, confirmed the need for a hydrophobic moiety in the correct orientation to maintain G protein-mediated signaling bias. This was exemplified through modification (removal/substitution) of the halogen group on the quinoline ring of MLS1547 or through substituting the quinoline ring with other bicyclic aromatic moieties. Several compounds were identified that exhibited a similar degree of G protein-mediated signaling bias as MLS1547, however, only one compound was found that exhibited greater (~three-fold) potency (**12**, Table 1) while still maintaining high bias. Interestingly, within the quinoline series of compounds, we identified a requirement for a hydroxyl group at the 8-position (Area 2, Figure 3B) in order to maintain potency as well as G protein-bias. These results suggest that the hydroxyl group forms critical interactions with yet to be identified segments of the D2R. This could be potentially exploited when formulating future derivatives of MLS1547.

Our previous data also suggested an important role for the pyridine moiety (Area 4, Figure 3B) of MLS1547 in terms of conferring potency, likely through the formation of a hydrogen bond to T412 of the D2R (Free et al., 2014). Cyclohexane substitutions of this pyridine moiety led to a complete loss of activity confirming the requirement for the aromaticity of this ring. Interestingly, moving the nitrogen from the ortho-position to either the meta- or para- positions lead to a decrease or loss of agonist activity, indicating that the position of the nitrogen is critical for D2R interactions. Notably, substitution at the para-position of this ring leads to a complete loss of activity further suggesting that critical positioning of this ring with D2R residues occurs. Interestingly, the addition of a second nitrogen was well-tolerated at the ortho- or meta- positions with meta substitution increasing potency, but decreasing bias. Our conclusion is that a nitrogen is required at the ortho-position of ring D for potent agonist activity and G protein bias, the addition of a second nitrogen at the meta-position can increase potency, but may decrease bias, and the addition of a nitrogen at the para position decreases all activity. It would be interesting to see if substitution of the meta position of ring D with other groups might lead to an enhancement of potency, yet maintain signaling bias.

Our results confirm and extend the pharmacophore model that we proposed to explain the biased signaling activity of MLS1547 (Free et al., 2014). In this model, docking of MLS1547 within the orthosteric binding site and a hydrophobic pocket near the extracellular portion of TM5 affects the tilting of this receptor region such that β-arrestin recruitment to the D2R is diminished. Such a model has also been suggested to explain biased signaling at the 5-HT2B serotonin receptor. Ergotamine is a β-arrestin-biased agonist at the 5-HT2B serotonin receptor, but non-biased at the 5-HT1B receptor. Wacker et al. (2013) crystalized both receptors in the presence of ergotamine and found that, in the 5HT_2B_ receptor structure, the extracellular portion of TM5 is tilted toward the orthosteric binding site compared to the 5HT_1B_ receptor structure. Thus, agonists that prevent such a tilting of TM5 might exhibit G protein signaling bias. As noted above, other G protein-biased agonists have been described for the D2R (Moller et al., 2014, 2017; Weichert et al., 2015; Chen et al., 2016; Bonifazi et al., 2017), although the specific structural bases for their biased activity were not proposed. It will thus be interesting to see if there are structural commonalities for promoting G protein-mediated biased signaling of the D2R or if multiple mechanisms exist.

## Author contributions

LC, RV, RF, YL, D-TL, PS, FL, YN, SL, AM, JA, KF, and DS participated in the research design. LC, RV, YL, D-TL, PS, YN, AM, and KF conducted experiments. RV, JA, and KF synthesized new chemical compounds. LC, RF, YL, D-TL, PS, FL, YN, AM, and KF performed data analysis. LC, RF, KF, and DS wrote or contributed to the writing of the manuscript. All authors contributed to and have approved the final manuscript.

### Conflict of interest statement

The authors declare that the research was conducted in the absence of any commercial or financial relationships that could be construed as a potential conflict of interest.

## Abbreviations

SAR: structure-activity relationship.
