# Supplementary material for: Structure-Activity Investigation of a G Protein-Biased Agonist Reveals Molecular Determinants for Biased Signaling of the D2 Dopamine Receptor
Source: Front Synaptic Neurosci. 2018 Feb 21;10:2. doi: 10.3389/fnsyn.2018.00002 (PMC5826336; doi:10.3389/fnsyn.2018.00002)
Supplement: Supplementary file 1 [file DataSheet1.pdf]

Structure-activity investigation of a G protein-biased agonist reveals molecular determinants for biased signaling of the D_2 dopamine receptor

SUPPLEMENTAL INFORMATION

Lani S. Chun[1], Rakesh H. Vekariya[2], R. Benjamin Free[1], Yun Li[3], Da-Ting Lin[3], Fang Liu[4], Yoon Namkung[5], Stephane A. Laporte[5], Amy E. Moritz[1], Jeffrey Aubé[2+], Kevin J. Frankowski[2+]*, and David R. Sibley[1]*

[1]Molecular Neuropharmacology Section, National Institute of Neurological Disorders and Stroke, National Institutes of Health, Bethesda, Maryland, USA

[2]Department of Medicinal Chemistry and Specialized Chemistry Center, University of Kansas, Lawrence, Kansas, USA

[3]Optical Imaging Core, National Institute of Drug Abuse, National Institutes of Health, Baltimore, MD, USA

[4]Molecular Neuroscience, Department of Neuroscience, Centre for Addiction and Mental Health, University of Toronto, Toronto, Ontario, Canada

[5]Department of Medicine, McGill University Health Center Research Institute, McGill University, Montreal, Quebec, Canada

[+]Current Address: Center for Integrative Chemical Biology and Drug Discovery, UNC Eshelman School of Pharmacy, University of North Carolina, Chapel Hill, NC, USA

General synthesis and analysis experimental details: All reagents were used as received from the following suppliers: Acros, Alfa Aesar, Aldrich, and Fisher Scientific. Acetonitrile and THF were purified using the Innovative Technology PureSolv solvent purification system. The ^{1}H and ^{13}C spectra were recorded on a Bruker Avance 400 MHz or 500 MHz spectrometer. Chemical shifts are reported in parts per million and were referenced to residual proton solvent signals. The infrared (IR) spectra were acquired as thin films using a universal ATR sampling accessory on a PerkinElmer Spectrum 100 FT-IR spectrometer and the absorbtion frequencies are reported in cm^{-1}. Melting points were determined on a Stanford Research Systems Optimelt automated melting point system interfaced through a PC and are uncorrected. Medium-pressure chromatography separations were performed using the Teledyne Isco CombiFlash R_F using RediSep R_F silica gel columns. TLC was performed on Analtech UNIPLATE silica gel GHLF plates (gypsum inorganic hard layer with fluorescence). TLC plates were developed using iodine vapor. Automated preparative RP HPLC purification was performed using an Agilent 1200 Mass-Directed Fractionation system (Prep Pump G1361 with gradient extension, make-up pump G1311A, pH modification pump G1311A, HTS PAL autosampler, UV-DAD detection G1315D, fraction collector G1364B, and Agilent 6120 quadrapole spectrometer G6120A). The preparative chromatography conditions included a Waters X-Bridge C_{18} column (19 × 150 mm, 5 μm, with 19 × 10-mm guard column), elution with a water and acetonitrile gradient, which increases 20% in acetonitrile content over 4 min at a flow rate of 20 mL/min (modified to pH 9.8 through addition of NH_4OH by auxiliary pump), and sample dilution in DMSO. The preparative gradient, triggering thresholds, and UV wavelength were selected according to the analytical RP HPLC analysis of each crude sample. The analytical method used an Agilent 1200 RRLC system with UV detection (Agilent 1200 DAD SL) and mass detection (Agilent 6224 TOF). The analytical method conditions included a Waters Aquity BEH C_{18} column (2.1 × 50 mm, 1.7 μm) and elution with a linear gradient of 5% acetonitrile in pH 9.8 buffered aqueous ammonium formate to 100% acetonitrile at 0.4 mL/min flow rate. Compound purity was measured on the basis of peak integration (area under the curve) from UV/Vis absorbance (at 214 nm), and compound identity was determined on the basis of mass analysis. All compounds used for biological studies have purity >90%. The analytical

HPLC system used is a dedicated instrument for assessing compound purity and routinely detects impurities as low as 0.1% that elute within the detection window. Any compounds with a measured purity of 100% were thus conservatively assigned a purity of > 99.8%. Any compounds purified by reverse-phase, preparative HPLC utilized the same solvent gradient and column material as the analytical conditions to minimize the possibility of impurities that were not detected in the analytical method.

General procedure A (Mannich route to quinolinol analogues): 4-Substituted piperazine (1 equiv) and formaldehyde (37% wt in H_2O, 3.75 equiv) were combined and the resulting mixture was stirred at 0 ˚C for 30 mins. To this mixture were added quinolinol (1 equiv) and pyridine (5 mL). The resulting reaction mixture was heated at 50 ˚C for 1 h. The mixture was cooled and solvent evaporated. The residue was dissolved in EtOAc (10 mL), washed with H_2O (2 × 5 mL), and the organic layer was concentrated. The crude product was purified by mass-directed, reverse-phase HPLC to afford the quinolinol analog.

General procedure B (reductive amination route for analog synthesis): To a solution of appropriate arylcarboxaldehyde (1 equiv) in THF (15 mL) at 0 ˚C, were added 4-substituted piperazine (1.1 equiv) and sodium triacetoxyborohydride (2 equiv). The resulting mixture was stirred at 0 ˚C for 5 mins and then brought to rt. After 15 mins 2 drops of acetic acid were added to the reaction mixture and further stirred at rt for 5 h. The mixture was brought to pH 9 using aqueous NaOH (1 M) and extracted with EtOAc (3 × 10 mL). The organic layers were combined and concentrated. The crude product was purified by mass-directed, reverse-phase HPLC to afford the desired analog.

General procedure C (acid chloride coupling route for analog synthesis): To a solution of aryl acid chloride (1.1 equiv) in THF (15 mL), were added 4-substituted piperazine (1 equiv), KI (1.1 equiv) and triethylamine (1 equiv). The resulting mixture was stirred at rt for 5 mins. The mixture was quenched by adding water (10 mL). The aqueous layer was extracted with EtOAc (3 × 10 mL). The organic layers were combined and concentrated. The crude product was purified by mass-directed, reverse-phase HPLC to afford the amide product.

General procedure D (benzylic alcohol route for analog synthesis): Triethylamine (1.5 equiv) was added to a solution of benzylic alcohol (1 equiv) and methanesulfonyl chloride (1.5 equiv) in CH_2Cl_2 (10 mL) under N_2 atmosphere at 0 °C. The reaction mixture was stirred at rt for 2 h and quenched with water. The aqueous layer was extracted with CH_2Cl_2 (3 × 10 mL). The combined organic layers were washed with H_2O (1 x 10 mL), dried (Na_2SO_4) and concentrate. The residue and 4-substituted piperazine (1.2 equiv) were dissolved in DMF (10 mL). To this solution was added triethylamine (1.2 equiv) and resulting mixture was stirred for 3 h at rt. The reaction was partitioned with water (20 mL) and the aqueous layer extracted with CH_2Cl_2 (3 × 10 mL). The combined organic layers were washed with H_2O (1 x 10 mL), dried (Na_2SO_4) and concentrated to give a residue. The crude product was purified by mass-directed, reverse-phase HPLC to afford the target compound.

General Procedure E (EDC coupling route for amide analog synthesis): To a solution of carboxylic acid (1 equiv) in CH_2Cl_2 (10 mL) was added 4-substituted piperazine (1.2 equiv) under N_2 atmosphere at 0 °C. The reaction mixture was stirred for 15 mins at 0 °C, then diisopropylethylamine (1.2 equiv) and EDC hydrochloride (1.2 equiv) were added at 0 °C. The reaction mixture was allowed to warm to rt and stirred for 5 h. Water (10 mL) was added, and the aqueous layer was extracted with CH_2Cl_2 (3 × 10 mL). The combined organic layers were washed with water (1 × 10 mL), dried (Na_2SO_4) and concentrated in vacuo. The crude product was purified by mass-directed, reverse-phase HPLC to afford the amide product.

General procedure F (amide reduction route for analog synthesis): To a solution of amide substrate (1 equiv) in THF (10 mL) was added $LiAlH_4$ (2M in THF, 3 equiv) at 0 ˚C under a N_2 atmosphere. The reaction mixture was heated at 50 ˚C for 3 h. The reaction was quenched by adding $Na_2SO_4 \cdot 10H_2O$, the mixture was filtered and concentrated in vacuo. The crude product was purified by mass-directed, reverse-phase HPLC to afford the amide reduction product.

General procedure G (HATU coupling for amide analog synthesis): To a solution of carboxylic acid (1 equiv) in DMF (10 mL) were added HATU (1 equiv) and DIEA (2 equiv) at rt. The resulting mixture was stirred for 5 mins, then, 4-substituted piperazine (1 equiv) was added and the reaction mixture stirred for 20 h. The reaction was quenched by

adding water (10 mL) and the aqueous layer was extracted with EtOAc (3 × 10 mL). The organic layers were combined, dried (Na_2SO_4) and concentrated in vacuo. The residue was purified on a medium-pressure chromatography purification system using a silica flash column (50:50 hexanes/EtOAc) to afford the amide product.

General procedure H (synthesis of noncommercial quinoline alcohols):[1] To a solution of the (3-amino-phenyl)methanol (1 equiv) in toluene (1.3 mL) were added 6M HCl (4.8 mL) and acrolein (2 equiv). The reaction mixture was heated at 100 ˚C for 2 h. The mixture was allowed to cool to room temperature. The aqueous layer was separated and neutralized with aqueous NaOH. The aqueous layer was extracted with EtOAc (3 × 10 mL). The organic layers were combined and concentrated. The residue was purified on a Combiflash purification system using a silica flash column (90:10 CH_2Cl_2/MeOH) to afford the quinoline substrate.

General procedure I (synthesis of noncommercial substituted piperazines):[2]

To a solution of *N*-Boc-piperazine (1 equiv) in toluene (1.5 mL) were added the appropriate bromo-substituted heterocyle (1 equiv), $Pd_2(dba)_3$ (2 mole %), (±)-BINAP (4 mole %) and NaO^{t}Bu (1.5 equiv). The reaction mixture was stirred at 70 ˚C for 90 mins. The mixture filtered through celite, washed with EtOAc and evaporated under reduced pressure. The residue was purified on a medium-pressure chromatography purification system using a silica flash column (80:20 Hexanes/EtOAc) to afford the Boc-protected product. To boc-protected compound in CH_2Cl_2 (1.5 mL) was added TFA (1.5 mL) and resulting mixture was stirred for 1 h. The solvent was evaporated under reduced pressure to afford the unprotected piperazine, which was used for next reaction without further purification.

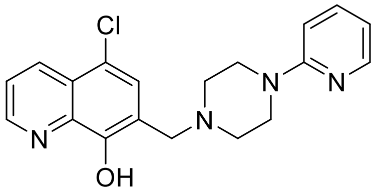

5-Chloro-7-((4-(pyridin-2-yl)piperazin-1-yl)methyl)quinolin-8-ol (**1**). 1-(2-Pyridyl)piperazine (0.182 g, 1.11 mmol, 1 equiv), formaldehyde (37% wt in H_2O, 0.125 g, 4.16 mmol, 3.75 equiv) and 5-chloro-8-quinolinol (0.200 g, 1.11 mmol, 1 equiv) were reacted according to general procedure A to afford **1** (0.348 g, 88%) as a white solid. Mp:

91–94 ˚C; TLC (5% MeOH/CH_2Cl_2): R_f = 0.4; ^{1}H NMR (500 MHz, $CDCl_3$) δ 8.92 (dd, *J* = 4.2, 1.6 Hz, 1H), 8.50 (dd, *J* = 8.6, 1.6 Hz, 1H), 8.19 (ddd, *J* = 4.7, 2.0, 0.8 Hz, 1H), 7.56 – 7.41 (m, 3H), 6.70 – 6.61 (m, 2H), 3.94 (s, 2H), 3.66 (t, *J* = 5.2 Hz, 4H), 2.85 – 2.72 (m, 4H); ^{13}C NMR (126 MHz, $CDCl_3$) δ 159.2, 152.0, 149.5, 148.1, 139.7, 137.8, 133.1, 127.8, 126.3, 122.3, 120.4, 113.9, 107.3, 52.7, 45.1, 41.2; IR (neat) 2837, 1593 cm^{-1}; HRMS (ESI) *m/z* calcd for $C_{19}H_{20}ClN_4O$ $[M + H]^+$: 355.1326, found: 356.1309; HPLC purity = 96.4%.

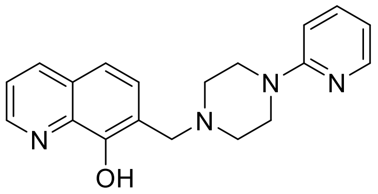

7-((4-(Pyridin-2-yl)piperazin-1-yl)methyl)quinolin-8-ol (**2**). This compound was synthesized during our previous work on this series.[3]

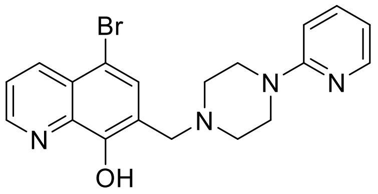

5-Bromo-7-((4-(pyridin-2-yl)piperazin-1-yl)methyl)quinolin-8-ol (**3**). 1-(2-Pyridyl)piperazine (0.146 g, 0.890 mmol, 1 equiv), formaldehyde (37% wt in H_2O, 0.100 g, 3.33 mmol, 3.75 equiv) and 5-bromo-8-quinolinol (0.200 g, 0.890 mmol, 1 equiv) were reacted according to general procedure A to afford **3** (0.154 g, 43%) as a brown solid. Mp: 89–91 ˚C; TLC (5% MeOH/CH_2Cl_2): R_f = 0.6; ^{1}H NMR (500 MHz, $CDCl_3$) δ 8.89 (dd, *J* = 4.2, 1.6 Hz, 1H), 8.45 (dd, *J* = 8.5, 1.6 Hz, 1H), 8.22 – 8.17 (m, 1H), 7.63 (s, 1H), 7.55 – 7.46 (m, 2H), 6.68 – 6.62 (m, 2H), 3.94 (s, 2H), 3.66 (t, *J* = 4.9 Hz, 4H), 2.84 – 2.71 (m, 4H); ^{13}C NMR (126 MHz, $CDCl_3$) δ 159.2, 152.7, 149.5, 148.1, 139.9, 137.8, 135.6, 131.4, 127.5, 122.7, 113.9, 109.9, 107.3, 52.7, 45.1, 41.2; IR (neat) 2837, 1593 cm^{-1}; HRMS (ESI) *m/z* calcd for $C_{19}H_{20}BrN_4O$ $[M + H]^+$: 401.0800, found: 401.0804; HPLC purity = 97.7%.

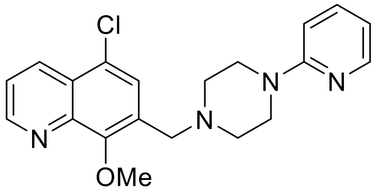

5-Chloro-8-methoxy-7-((4-(pyridin-2-yl)piperazin-1-yl)methyl)quinolone (**4**). To a solution of 5-chloro-7-((4-(pyridin-2-yl)piperazin-1-yl)methyl)quinolin-8-ol (0.0500 g,

0.140 mmol, 1 equiv) in DMF (5 mL), was added NaH (60% dispersion in mineral oil, 0.0085 g, 0.210 mmol, 1.5 equiv) at 0 ˚C and stirred for 30 mins. Then, methyl iodide (0.060 g, 0.420 mmol, 3 equiv) was added to reaction mixture at 0 ˚C and the mixture was further stirred at rt for 4 h. The mixture was quenched by adding H_2O (10 mL). The aqueous layer was extracted with EtOAc (3 × 10 mL). The organic layers were combined and concentrated. The crude product was purified by mass directed HPLC to afford **4** (0.0152 g, 26%) as an orange solid. Mp: 118–120 ˚C; TLC (5% MeOH/CH_2Cl_2): R_f = 0.6; ^{1}H NMR (500 MHz, $CDCl_3$) δ 8.98 (dd, *J* = 4.2, 1.7 Hz, 1H), 8.55 (dd, *J* = 8.5, 1.7 Hz, 1H), 8.19 (ddd, *J* = 5.0, 2.1, 0.9 Hz, 1H), 7.82 (s, 1H), 7.55 – 7.44 (m, 2H), 6.68 – 6.59 (m, 2H), 4.13 (s, 3H), 3.82 (s, 2H), 3.58 (s, 4H), 2.66 (s, 4H); ^{13}C NMR (126 MHz, $CDCl_3$) δ 153.7, 150.2, 148.1, 143.5, 137.6, 133.4, 128.5, 127.0, 126.1, 121.9, 113.4, 107.3, 63.0, 56.1, 53.1, 45.4; IR (neat) 2829, 1593 cm^{-1}; HRMS (ESI) *m/z* calcd for $C_{20}H_{22}ClN_4O$ [M + H]$^+$: 369.1482, found: 369.1477; HPLC purity = 98.6%.

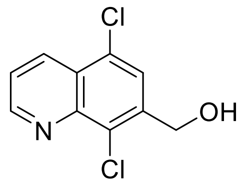

(5,8-Dichloroquinolin-7-yl)methanol (**S-5**): (3-Amino-2,5-dichlorophenyl)methanol (0.600 g, 3.12 mmol, 1 equiv), acrolein (0.875 g, 15.6 mmol, 5 equiv) and 6M HCl (15.7 mL) were reacted according to general procedure H to afford **S-5** (0.29 g, 41%) as a light yellow solid. Mp: 165–167 ˚C; TLC (10% MeOH/CH_2Cl_2): R_f = 0.7; ^{1}H NMR (400 MHz, $CDCl_3$) δ 9.09 (dd, *J* = 4.2, 1.7 Hz, 1H), 8.61 (dd, *J* = 8.5, 1.6 Hz, 1H), 7.92 (s, 1H), 7.60 (dd, *J* = 8.5, 4.2 Hz, 1H), 5.07 (s, 2H); ^{13}C NMR (101 MHz, $CDCl_3$) δ 151.7, 144.9, 140.1, 133.8, 133.2, 130.5, 126.7, 126.0, 122.6, 62.6; IR (neat) 3300, 1594 cm^{-1}; HRMS (ESI) *m/z* calcd for $C_{10}H_8Cl_2NO$ [M + H]$^+$: 227.9983, found: 227.9980.

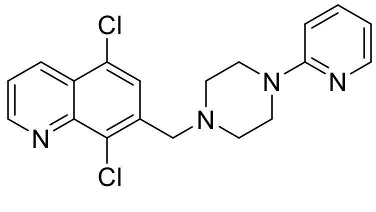

5,8-Dichloro-7-((4-(pyridin-2-yl)piperazin-1-yl)methyl)quinolone (**5**).

(5,8-Dichloroquinolin-7-yl)methanol (0.0200 g, 0.0877 mmol, 1 equiv), methanesulfonyl chloride (0.0190 g, 0.132 mmol, 1.5 equiv), triethylamine (0.0133, 0.132 mmol, 1.5 equiv) and 1-(2-pyridyl)piperazine (0.0171 g, 0.105 mmol, 1.2 equiv) were reacted according to general procedure D to afford **5** (0.0220 g, 67%) as a white solid. Mp: 180–182 ˚C; TLC (5% MeOH/CH_2Cl_2): R_f = 0.7; ^{1}H NMR (500 MHz, $CDCl_3$) δ 9.09 (dd, *J* = 4.2, 1.7 Hz, 1H), 8.60 (dd, *J* = 8.5, 1.7 Hz, 1H), 8.20 (ddd, *J* = 4.9, 2.0, 0.9 Hz, 1H), 7.97 (s, 1H), 7.59 (dd, *J* = 8.5, 4.2 Hz, 1H), 7.49 (ddd, *J* = 9.0, 7.2, 2.1 Hz, 1H), 6.69 – 6.60 (m, 2H), 3.95 (s, 2H), 3.67 – 3.57 (m, 4H), 2.77 – 2.65 (m, 4H); ^{13}C NMR (126 MHz, $CDCl_3$) δ 159.6, 151.5, 148.1, 145.3, 138.2, 137.7, 133.6, 131.7, 130.1, 128.0, 126.6, 122.5, 113.5, 107.3, 59.5, 53.2, 45.4; IR (neat) 1593, 1480 cm^{-1}; HRMS (ESI) *m/z* calcd for $C_{19}H_{19}Cl_2N_4$ $[M + H]^+$: 373.0987, found: 373.0977; HPLC purity = 93.6%.

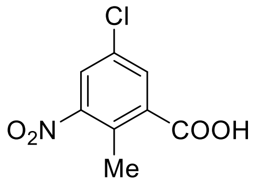

5-Chloro-2-methyl-3-nitrobenzoic acid (**S-6’’**): To 5-chloro-2-methylbenzoic acid (0.412 g, 2.21 mmol, 1 equiv) in H_2SO_4 was dropwise added mixture HNO_3 and H_2SO_4 at 0 ˚C. The resulting mixture was brought to room temperature and further stirred for 7 h. To the mixture ice cold water was added and the resulting precipitate was filtered. The precipitate was purified on a Combiflash purification system using a silica flash column (85:15 Hexanes/EtOAc; 0.5% acetic acid) to afford **S-6’’** (0.75 g, 60%). TLC (50% Hexanes/EtOAc): R_f = 0.2; ^{1}H NMR (400 MHz, DMSO-*d*$_6$) δ δ 7.24 (d, *J* = 2.3 Hz, 1H), 7.17 (d, *J* = 2.3 Hz, 1H), 1.75 (s, 3H); HRMS (ESI) *m/z* calcd for $C_8H_5ClNO_4$ $[M - H]^-$: 213.9907, found: 213.9909.

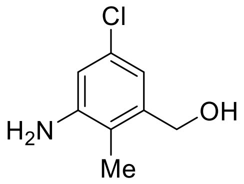

(3-Amino-5-chloro-2-methylphenyl)methanol (**S-6’**): To a solution of **S-6’’** (0.730 g, 3.40 mmol, 1 equiv) in H_2O:EtOH (1:1, 6 mL) was added NH_4Cl (1.81 g, 3.39 mmol, 10 equiv) at 80 ˚C. Fe powder (0.380 g, 6.80 mmol, 2 equiv) was added portionwise to the reaction mixture and further stirred for 2 h at 80 ˚C. The mixture filtered over celite, washed

with EtOH and evaporated under reduced pressure to give crude 3-amino-5-chloro-2-methylbenzoic acid (0.55 g, 87%). The crude carboxylic acid was subjected to next step without further purification. Thus, 3-Amino-5-chloro-2-methylbenzoic acid (0.540 g, 2.50 mmol, 1 equiv) and $LiAlH_4$ (2M in THF, 0.552 g, 14.5 mmol, 5 equiv) were reacted according to general procedure F to afford **S-6’** (0.230 g, 46%). TLC (50% Hexanes/EtOAc, 2 drops of NH_4OH): R_f = 0.6; ^{1}H NMR (400 MHz, DMSO-d_6) δ 5.91 (d, *J* = 2.2 Hz, 1H), 5.87 (d, *J* = 2.2 Hz, 1H), 3.73 (s, 2H), 1.25 (s, 3H); ^{13}C NMR (101 MHz, $CDCl_3$) δ 139.1, 132.9, 123.0, 110.4, 108.8, 105.7, 53.9, 2.3; HRMS (ESI) *m/z* calcd for $C_8H_{11}ClNO$ [M + H]$^+$: 172.0529, found: 172.0531.

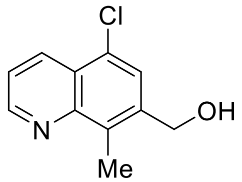

(5-Chloro-8-methylquinolin-7-yl)methanol (**S-6**): (3-Amino-5-chloro-2-methylphenyl)methanol (0.219 g, 1.28 mmol, 1 equiv), acrolein (0.360 g, 6.40 mmol, 5 equiv), and 6M HCl (6.5 mL) were reacted according to general procedure H to afford **S-6** (0.090 g, 34%). TLC (50% Hexanes/EtOAc): R_f = 0.5; ^{1}H NMR (400 MHz, $CDCl_3$) δ δ 8.98 (dd, *J* = 4.2, 1.8 Hz, 1H), 8.56 (dd, *J* = 8.5, 1.8 Hz, 1H), 7.79 (s, 1H), 7.50 (dd, *J* = 8.5, 4.1 Hz, 1H), 4.94 (d, *J* = 4.8 Hz, 2H), 2.77 (s, 3H); HRMS (ESI) *m/z* calcd for $C_{11}H_{11}ClNO$ [M + H]$^+$: 208.0529, found: 208.0522.

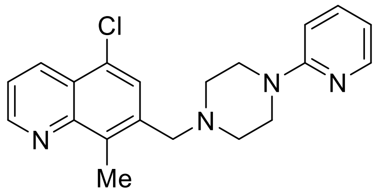

5-Chloro-8-methyl-7-((4-(pyridin-2-yl)piperazin-1-yl)methyl)quinolone (**6**). (5-Chloro-8-methylquinolin-7-yl)methanol (0.0100 g, 0.0481 mmol, 1 equiv), methanesulfonyl chloride (0.0110 g, 0.072 mmol, 1.5 equiv), triethylamine (0.00800, 0.072 mmol, 1.5 equiv) and 1-(2-pyridyl)piperazine (0.0100 g, 0.058 mmol, 1.2 equiv) were reacted according to general procedure D to afford **6** (0.0095 g, 56%) as a white solid. Mp: 166–168 ˚C; TLC (5% MeOH/CH_2Cl_2): R_f = 0.7; ^{1}H NMR (500 MHz, $CDCl_3$) δ 8.98 (dd, *J* = 4.2, 1.8 Hz, 1H), 8.56 (dd, *J* = 8.5, 1.8 Hz, 1H), 8.19 (ddd, *J* = 4.9, 2.0, 0.9 Hz, 1H), 7.77 (s, 1H), 7.54 – 7.43 (m, 2H), 6.69 – 6.59 (m, 2H), 3.74 (s, 2H), 3.57 (t, *J* = 5.0 Hz,

4H), 2.83 (s, 3H), 2.63 (d, *J* = 5.0 Hz, 4H); ^{13}C NMR (126 MHz, $CDCl_3$) δ 159.6, 149.8, 148.2, 148.1, 137.6, 135.3, 133.1, 128.5, 125.5, 121.4, 113.4, 107.2, 60.4, 53.2, 45.4, 13.2; IR (neat) 2828, 1593 cm^{-1}; HRMS (ESI) *m/z* calcd for $C_{20}H_{22}ClN_4$ $[M + H]^+$: 353.1533, found: 353.1528; HPLC purity >99.8%.

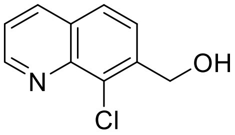

(8-Chloroquinolin-7-yl)methanol (**S-7**): (3-Amino-2-chlorophenyl)methanol (0.150 g, 0.951 mmol, 1 equiv) and acrolein (0.106 g, 1.90 mmol, 2 equiv) were reacted according to general procedure H to afford **S-7** (0.02 g, 11%) as a white solid. Mp: 111–113 ˚C; TLC (10% MeOH/CH_2Cl_2): R_f = 0.7; ^1H NMR (400 MHz, $CDCl_3$) δ 9.04 (dd, *J* = 4.2, 1.7 Hz, 1H), 8.18 (dd, *J* = 8.2, 1.7 Hz, 1H), 7.78 (d, *J* = 0.6 Hz, 2H), 7.47 (dd, *J* = 8.2, 4.2 Hz, 1H), 5.07 (s, 2H); ^{13}C NMR (101 MHz, $CDCl_3$) δ 151.2, 144.4, 139.9, 136.6, 130.8, 128.8, 126.7, 126.3, 121.9, 63.1; IR (neat) 3307, 1616 cm^{-1}; HRMS (ESI) *m/z* calcd for $C_{10}H_9ClNO$ $[M + H]^+$: 194.0373, found: 194.0369.

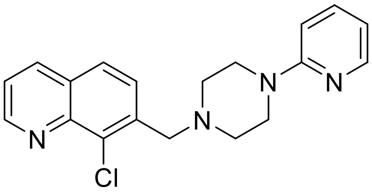

8-Chloro-7-((4-(pyridin-2-yl)piperazin-1-yl)methyl)quinoline (**7**). Triethylamine (0.048, 0.475 mmol, 1.5 equiv), (8-chloroquinolin-7-yl)methanol **S-7** (0.050 g, 0.317 mmol, 1 equiv), methanesulfonyl chloride (0.069 g, 0.475 mmol, 1.5 equiv), 1-(2-pyridyl)piperazine (0.062 g, 0.380 mmol, 1.2 equiv) and triethylamine (0.0385, 0.380 mmol, 1.2 equiv) were reacted according to general procedure D to afford **7** (0.043 g, 40%) as a white solid. Mp: 133–135 ˚C; TLC (5% MeOH/CH_2Cl_2): R_f = 0.6; ^1H NMR (500 MHz, $CDCl_3$) δ 9.06 (dd, *J* = 4.3, 1.7 Hz, 1H), 8.23 – 8.15 (m, 2H), 7.85 (s, 1H), 7.77 (d, *J* = 8.3 Hz, 1H), 7.52 – 7.41 (m, 2H), 6.69 – 6.57 (m, 2H), 4.00 (s, 2H), 3.60 (s, 4H), 2.72 (s, 4H); ^{13}C NMR (126 MHz, $CDCl_3$) δ 159.5, 151.1, 148.0, 144.8, 137.7, 136.5, 132.9, 128.5, 126.3, 121.8, 113.5, 111.9, 107.3, 59.7, 53.2, 45.3; IR (neat) 1593, 1437 cm^{-1}; HRMS (ESI) *m/z* calcd for $C_{19}H_{20}ClN_4$ $[M + H]^+$: 339.1376, found: 339.1368; HPLC purity > 99.8%.

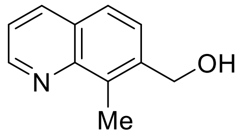

RHV-226

(8-Methylquinolin-7-yl)methanol (**S-8**): 3-Amino-2-methylbenzylalcohol (1.00 g, 7.28 mmol, 1 equiv), acrolein (2.04 g, 36.4 mmol, 5 equiv) and 6M HCl (25.5 mL) were reacted according to general procedure H to afford **S-8** (0.33 g, 26%) as a red oil. TLC (50% Hexanes/EtOAc): R_f = 0.4; ^{1}H NMR (400 MHz, $CDCl_3$) δ δ 8.95 (dd, *J* = 4.2, 1.8 Hz, 1H), 8.13 (dd, *J* = 8.2, 1.8 Hz, 1H), 7.73 – 7.63 (m, 2H), 7.39 (dd, *J* = 8.2, 4.2 Hz, 1H), 4.96 (s, 2H), 2.83 (s, 3H); ^{13}C NMR (101 MHz, $CDCl_3$) δ 149.6, 147.3, 139.2, 136.4, 134.5, 127.7, 126.5, 125.7, 120.9, 63.7, 12.7; IR (neat) 3312, 1599 cm^{-1}; HRMS (ESI) *m/z* calcd for $C_{11}H_{12}NO$ [M + H]$^+$: 174.0919, found: 174.0915.

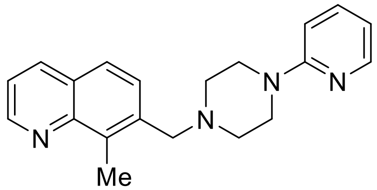

8-Methyl-7-((4-(pyridin-2-yl)piperazin-1-yl)methyl)quinolone (**8**). (8-Methylquinolin-7-yl)methanol (0.0250 g, 0.144 mmol, 1 equiv), methanesulfonyl chloride (0.0310 g, 0.216 mmol, 1.5 equiv), triethylamine (0.0220, 0.216 mmol, 1.5 equiv) and 1-(2-pyridyl)piperazine (0.0282 g, 0.173 mmol, 1.2 equiv) were reacted according to general procedure D to afford **8** (0.0380 g, 83%) as a white solid. Mp: 102–103 ˚C; TLC (5% MeOH/CH_2Cl_2): R_f = 0.7; ^{1}H NMR (500 MHz, $CDCl_3$) δ 8.95 (dd, *J* = 4.2, 1.8 Hz, 1H), 8.18 (ddd, *J* = 4.9, 2.0, 0.9 Hz, 1H), 8.13 (dd, *J* = 8.3, 1.8 Hz, 1H), 7.70 – 7.59 (m, 2H), 7.47 (ddd, *J* = 8.9, 7.1, 2.0 Hz, 1H), 7.39 (dd, *J* = 8.2, 4.2 Hz, 1H), 6.66 – 6.59 (m, 2H), 3.86 (s, 2H), 3.59 (s, 4H), 2.88 (s, 3H), 2.70 (s, 4H); ^{13}C NMR (126 MHz, $CDCl_3$) δ 159.4, 149.4, 148.0, 147.6, 137.7, 136.3, 129.0, 127.7, 125.3, 120.9, 118.0, 115.7, 113.5, 107.3, 60.6, 53.0, 45.1, 13.4; IR (neat) 2824, 1593 cm^{-1}; HRMS (ESI) *m/z* calcd for $C_{20}H_{23}N_4$ [M + H]$^+$: 319.1923, found: 319.1918; HPLC purity > 99.8%.

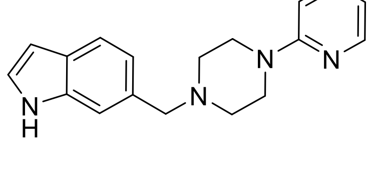

6-((4-(Pyridin-2-yl)piperazin-1-yl)methyl)-1H-indole (**9**). (1H-indol-6-yl)(4-(pyridin-2-yl)piperazin-1-yl)methanone **13** (0.0300 g, 0.098 mmol, 1 equiv) and $LiAlH_4$ (2M in THF, 0.0110 g, 0.294, 3 equiv) were reacted according to general procedure F to afford **9** (0.0082 g, 29%) as a brown oil. TLC (5% MeOH/CH_2Cl_2): R_f = 0.4; ^{1}H NMR (400 MHz, $CDCl_3$) δ 8.31 (s, 1H), 8.18 (ddd, *J* = 5.1, 2.0, 0.9 Hz, 1H), 7.60 (d, *J* = 8.1 Hz, 1H), 7.52 (s, 1H), 7.50 – 7.43 (m, 1H), 7.22 (dd, *J* = 3.2, 2.4 Hz, 1H), 7.10 (dd, *J* = 8.1, 1.5 Hz, 1H), 6.63 (s, 1H), 6.63 – 6.60 (m, 1H), 6.54 (ddd, *J* = 3.1, 2.1, 1.0 Hz, 1H), 3.76 (s, 2H), 3.62 (t, *J* = 5.1 Hz, 5H), 2.68 (t, *J* = 5.1 Hz, 4H); ^{13}C NMR (126 MHz, $CDCl_3$) δ 159.6, 148.1, 137.6, 136.1, 131.0, 127.4, 124.6, 121.8, 120.5, 113.4, 112.0, 107.2, 102.6, 63.7, 52.9, 45.1; IR (neat) 2825, 1593 cm^{-1}; HRMS (ESI) *m/z* calcd for $C_{18}H_{21}N_4$ $[M + H]^+$: 293.1766, found: 293.1756; HPLC purity = 96.6%.

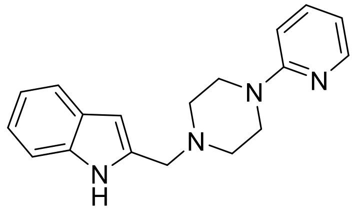

2-((4-(Pyridin-2-yl)piperazin-1-yl)methyl)-1H-indole (**10**): (1H-indol-2-yl)(4-(pyridin-2-yl)piperazin-1-yl)methanone **14** (0.0300 g, 0.098 mmol, 1 equiv) and $LiAlH_4$ (2M in THF, 0.0110 g, 0.294, 3 equiv) were reacted according to general procedure F to afford **10** (0.0119 g, 42%) as a light brown oil. TLC (5% MeOH/CH_2Cl_2): R_f = 0.5; ^{1}H NMR (500 MHz, $CDCl_3$) δ 8.69 (s, 1H), 8.19 (ddd, *J* = 4.5, 2.0, 1.0 Hz, 1H), 7.57 (dd, *J* = 7.9, 1.1 Hz, 1H), 7.48 (ddd, *J* = 8.6, 7.2, 2.0 Hz, 1H), 7.37 (dd, *J* = 8.1, 0.9 Hz, 1H), 7.17 (ddd, *J* = 8.2, 7.1, 1.3 Hz, 1H), 7.09 (ddd, *J* = 8.0, 7.1, 1.1 Hz, 1H), 6.67 – 6.60 (m, 2H), 6.39 (dd, *J* = 2.1, 1.0 Hz, 1H), 3.74 (d, *J* = 0.8 Hz, 2H), 3.63 – 3.53 (m, 4H), 2.66 – 2.58 (m, 4H); ^{13}C NMR (126 MHz, $CDCl_3$) δ 159.5, 148.1, 137.7, 136.3, 135.1, 128.4, 121.9, 120.3, 119.8, 113.6, 110.9, 107.3, 102.1, 56.0, 53.1, 45.2; IR (neat) 3238, 1594 cm^{-1}; HRMS (ESI) *m/z* calcd for $C_{18}H_{21}N_4$ $[M + H]^+$: 293.1766, found: 293.1754; HPLC purity >99.8%.

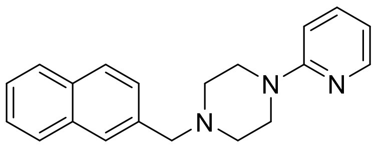

1-(Naphthalen-2-ylmethyl)-4-(pyridin-2-yl)piperazine (**11**): This compound was synthesized as previously described.[3] HPLC purity = 98.1%.

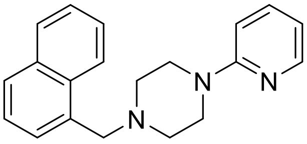

1-(Naphthalen-1-ylmethyl)-4-(pyridin-2-yl)piperazine (**12**): This compound was synthesized as previously described.[3] HPLC purity = 99.0%.

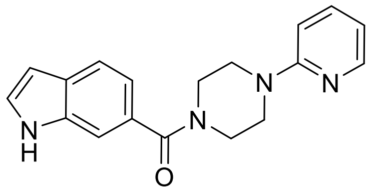

(1H-Indol-6-yl)(4-(pyridin-2-yl)piperazin-1-yl)methanone (**13**): Indole-6-carboxylic acid (0.0500 g, 0.31 mmol, 1 equiv), HATU (0.118 g, 0.31 mmol, 1 equiv), DIEA (0.080 g, 0.62 mmol, 2 equiv) and 1-(2-pyridyl)piperazine (0.0510 g, 0.31 mmol, 1 equiv) were reacted according to general procedure G to afford **13** (0.050 g, 53%) as a brown solid. Mp: 203–205 ˚C; TLC (5% MeOH/CH_2Cl_2): R_f = 0.4; ^{1}H NMR (400 MHz, $CDCl_3$) δ 8.66 (s, 1H), 8.22 – 8.16 (m, 1H), 7.65 (dt, *J* = 8.2, 0.8 Hz, 1H), 7.56 (dt, *J* = 1.6, 0.8 Hz, 1H), 7.52 (ddd, *J* = 8.5, 7.2, 2.0 Hz, 1H), 7.30 (dd, *J* = 3.3, 2.4 Hz, 1H), 7.18 (dd, *J* = 8.1, 1.4 Hz, 1H), 6.72 – 6.64 (m, 2H), 6.58 (ddd, *J* = 3.1, 2.0, 0.9 Hz, 1H), 3.78 (s, 4H), 3.60 (s, 4H); ^{13}C NMR (101 MHz, $CDCl_3$) δ 172.0, 138.2, 135.5, 129.4, 129.0, 126.4, 120.7, 119.0, 114.0, 111.1, 107.7, 102.9, 100.1, 45.8; IR (neat) 3169, 1592 cm^{-1}; HRMS (ESI) *m/z* calcd for $C_{18}H_{19}N_4O$ $[M + H]^+$: 307.1559, found: 307.1554; HPLC purity = 98.7%.

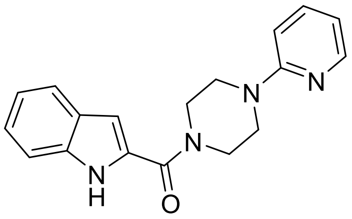

(1H-Indol-2-yl)(4-(pyridin-2-yl)piperazin-1-yl)methanone (**14**): Indole-2-carboxylic acid (0.200 g, 1.24 mmol, 1 equiv), HATU (0.471 g, 1.24 mmol, 1 equiv), DIEA (0.321 g, 2.48 mmol, 2 equiv) and 1-(2-pyridyl)piperazine (0.202 g, 1.24 mmol, 1 equiv) were reacted according to general procedure G to afford **14** (0.130 g, 34%) as a white solid. Mp: 206–207 ˚C; TLC (5% MeOH/CH_2Cl_2): R_f = 0.7; ^{1}H NMR (400 MHz, $CDCl_3$) δ 9.31

(s, 1H), 8.22 (ddd, J = 4.9, 2.0, 1.0 Hz, 1H), 7.67 (dd, J = 8.1, 1.0 Hz, 1H), 7.53 (ddd, J = 8.5, 7.2, 2.0 Hz, 1H), 7.44 (dd, J = 8.3, 1.0 Hz, 1H), 7.30 (ddd, J = 8.3, 7.0, 1.2 Hz, 1H), 7.15 (ddd, J = 8.0, 7.0, 1.0 Hz, 1H), 6.84 (dd, J = 2.1, 0.9 Hz, 1H), 6.73 – 6.64 (m, 2H), 4.08 (s, 4H), 3.79 – 3.65 (m, 4H); ^{13}C NMR (101 MHz, $CDCl_3$) δ 162.7, 159.1, 148.2, 137.9, 135.8, 129.3, 127.7, 124.7, 122.1, 120.8, 114.0, 111.9, 107.3, 105.6, 45.3; IR (neat) 3251, 1590 cm^{-1}; HRMS (ESI) *m/z* calcd for $C_{18}H_{19}N_4O$ [M + H]$^+$: 307.1559, found: 307.1543; HPLC purity >99.8%.

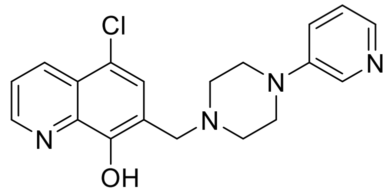

5-Chloro-7-((4-(pyridin-3-yl)piperazin-1-yl)methyl)quinolin-8-ol (**15**): 1-Pyridin-3-yl-piperazine (0.0180 g, 0.111 mmol, 1 equiv), formaldehyde (37% wt in H_2O, 0.0130 g, 0.416 mmol, 3.75 equiv) and 5-chloro-8-quinolinol (0.0200 g, 0.111 mmol, 1 equiv) were reacted according to general procedure A to afford **15** (0.0327 g, 83%) as a yellow solid. Mp: 103–105 ˚C; TLC (5% MeOH/CH_2Cl_2): R_f = 0.5; ^{1}H NMR (500 MHz, $CDCl_3$) δ 8.91 (dd, J = 4.2, 1.6 Hz, 1H), 8.50 (dd, J = 8.5, 1.6 Hz, 1H), 8.32 (dd, J = 2.3, 1.3 Hz, 1H), 8.13 (dd, J = 3.8, 2.2 Hz, 1H), 7.53 (dd, J = 8.5, 4.2 Hz, 1H), 7.46 (s, 1H), 7.20 – 7.17 (m, 2H), 3.93 (s, 2H), 3.36 – 3.28 (m, 4H), 2.86 – 2.78 (m, 4H); ^{13}C NMR (126 MHz, $CDCl_3$) δ 151.7, 149.4, 146.8, 141.2, 139.6, 138.9, 133.1, 127.9, 126.2, 123.7, 122.8, 122.4, 120.4, 118.1, 59.1, 52.6, 48.6; IR (neat) 2830, 1245 cm^{-1}; HRMS (ESI) *m/z* calcd for $C_{19}H_{20}ClN_4O$ [M + H]$^+$: 355.1326, found: 356.1306; HPLC purity >99.8%.

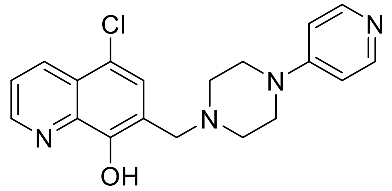

5-Chloro-7-((4-(pyridin-4-yl)piperazin-1-yl)methyl)quinolin-8-ol (**16**): 1-(4-Pyridyl)piperazine (0.0180 g, 0.111 mmol, 1 equiv), formaldehyde (37% wt in H_2O, 0.0130 g, 0.416 mmol, 3.75 equiv) and 5-chloro-8-quinolinol (0.0200 g, 0.111 mmol, 1 equiv) were reacted according to general procedure A to afford **16** (0.0129 g, 33%) as a brown solid. Mp: 97–99 ˚C; TLC (5% MeOH/CH_2Cl_2): R_f = 0.2; ^{1}H NMR (500 MHz, $CDCl_3$) δ

8.90 (dd, J = 4.2, 1.6 Hz, 1H), 8.50 (dd, J = 8.5, 1.6 Hz, 1H), 8.31 – 8.26 (m, 2H), 7.54 (dd, J = 8.5, 4.2 Hz, 1H), 7.48 (s, 1H), 6.68 (d, J = 6.7 Hz, 2H), 3.89 (s, 2H), 3.48 – 3.40 (m, 4H), 2.79 – 2.72 (m, 4H); ^{13}C NMR (126 MHz, $CDCl_3$) δ 155.0, 151.3, 149.7, 149.3, 139.4, 133.2, 128.1, 126.1, 122.4, 120.5, 118.3, 108.5, 58.5, 52.4, 46.1; IR (neat) 1597 cm^{-1}; HRMS (ESI) *m/z* calcd for $C_{19}H_{20}ClN_4O$ $[M + H]^+$: 355.1326, found: 356.1304; HPLC purity = 95.7%.

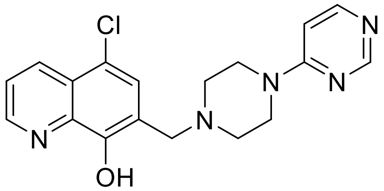

5-Chloro-7-((4-(pyrimidin-4-yl)piperazin-1-yl)methyl)quinolin-8-ol (**17**): 4-(Piperazin-1-yl)pyrimidine•2HCl (0.0260 g, 0.111 mmol, 1 equiv), formaldehyde (37% wt in H_2O, 0.0130 g, 0.416 mmol, 3.75 equiv) and 5-chloro-8-quinolinol (0.0200 g, 0.111 mmol, 1 equiv) were reacted according to general procedure A to afford **17** (0.00480 g, 12%) as a yellow oil. TLC (5% MeOH/CH_2Cl_2): R_f = 0.8; ^{1}H NMR (500 MHz, $CDCl_3$) δ 8.90 (dd, J = 4.2, 1.6 Hz, 1H), 8.60 (dd, J = 1.2, 0.6 Hz, 1H), 8.50 (dd, J = 8.6, 1.6 Hz, 1H), 8.21 (dd, J = 6.3, 0.6 Hz, 1H), 7.54 (dd, J = 8.5, 4.2 Hz, 1H), 7.47 (s, 1H), 6.50 (dd, J = 6.3, 1.3 Hz, 1H), 3.89 (s, 2H), 3.79 – 3.69 (m, 4H), 2.73 – 2.67 (m, 4H); ^{13}C NMR (126 MHz, $CDCl_3$) δ 161.3, 158.5, 155.9, 151.3, 149.3, 139.5, 133.1, 128.0, 126.1, 122.4, 120.5, 118.2, 103.1, 58.6, 52.5, 43.7; IR (neat) 1589 cm^{-1}; HRMS (ESI) *m/z* calcd for $C_{18}H_{19}ClN_5O$ $[M + H]^+$: 356.1278, found: 356.1245; HPLC purity = 98.2%.

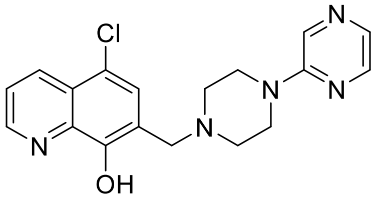

5-Chloro-7-((4-(pyrazin-2-yl)piperazin-1-yl)methyl)quinolin-8-ol (**18**): 1-(2-Pyrazinyl)piperazine (0.183 g, 1.11 mmol, 1 equiv), formaldehyde (37% wt in H_2O, 0.125 g, 4.16 mmol, 3.75 equiv) and 5-chloro-8-quinolinol (0.200 g, 1.11 mmol, 1 equiv) were reacted according to general procedure A to afford **18** (0.219 g, 55%) as a brown solid. Mp: 94–96 ˚C; TLC (5% MeOH/CH_2Cl_2): R_f = 0.3; ^{1}H NMR (500 MHz, $CDCl_3$) δ 8.90 (dd, J = 4.2, 1.6 Hz, 1H), 8.50 (dd, J = 8.6, 1.6 Hz, 1H), 8.14 (d, J = 1.5 Hz, 1H), 8.07 (dd,

J = 2.6, 1.5 Hz, 1H), 7.87 (d, *J* = 2.6 Hz, 1H), 7.53 (dd, *J* = 8.5, 4.2 Hz, 1H), 7.46 (s, 1H), 3.91 (s, 2H), 3.75 – 3.65 (m, 4H), 2.75 (t, *J* = 5.0 Hz, 4H); ^{13}C NMR (126 MHz, $CDCl_3$) δ 159.2, 152.0, 149.5, 148.1, 139.7, 137.8, 133.1, 127.8, 126.3, 122.3, 120.4, 113.9, 107.3, 52.7, 45.1, 41.2; IR (neat) 2837, 1576 cm^{-1}; HRMS (ESI) *m/z* calcd for $C_{18}H_{19}ClN_5O$ [M + H]$^+$: 356.1278, found: 356.1273; HPLC purity = 98.3%

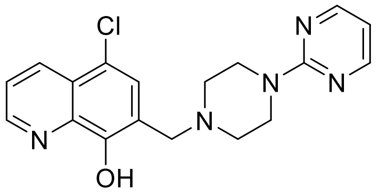

5-Chloro-7-((4-(pyrimidin-2-yl)piperazin-1-yl)methyl)quinolin-8-ol (**19**): 1-(2-Pyrimidyl)piperazine (0.183 g, 1.11 mmol, 1 equiv), formaldehyde (37% wt in H_2O, 0.125 g, 4.16 mmol, 3.75 equiv) and 5-chloro-8-quinolinol (0.200 g, 1.11 mmol, 1 equiv) were reacted according to general procedure A to afford **19** (0.112 g, 28%) as a brown solid. Mp: 148–150 ˚C; TLC (5% MeOH/CH_2Cl_2): R_f = 0.5; ^{1}H NMR (500 MHz, $CDCl_3$) δ 8.92 (dd, *J* = 4.3, 1.6 Hz, 1H), 8.49 (dd, *J* = 8.6, 1.6 Hz, 1H), 8.31 (d, *J* = 4.7 Hz, 2H), 7.52 (dd, *J* = 8.6, 4.1 Hz, 1H), 7.42 (s, 1H), 6.51 (t, *J* = 4.8 Hz, 1H), 3.92 (d, *J* = 11.4 Hz, 6H), 2.71 (s, 4H); ^{13}C NMR (126 MHz, $CDCl_3$) δ 161.6, 157.9, 152.0, 149.5, 139.7, 133.0, 127.8, 126.2, 122.3, 120.3, 110.4, 59.6, 52.8, 43.6; IR (neat) 3385, 1586 cm^{-1}; HRMS (ESI) *m/z* calcd for $C_{18}H_{19}ClN_5O$ [M + H]$^+$: 356.1278, found: 356.1269; HPLC purity >99.8%.

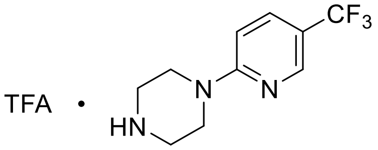

1-(5-(Trifluoromethyl)pyridin-2-yl)piperazine·TFA (**S-20**): *N*-Boc-piperazine (0.412 g, 2.21 mmol, 1 equiv), 2-bromo-5-trifluoromethylpyridine (0.500 g, 2.21 mmol, 1 equiv), $Pd_2(dba)_3$ (0.0400 g, 0.044 mmol, 2 mole %), (±)-BINAP (0.0550 g, 0.089 mmol, 4 mole %) and NaO^{t}Bu (0.320 g, 3.32 mmol, 1.5 equiv) were reacted according to general procedure I to afford Boc-protected **S-20** (0.53 g, 72%) as a yellow solid. The Boc-protected compound was reacted according to general procedure I to afford **S-20**, which was used for next reaction without further purification.

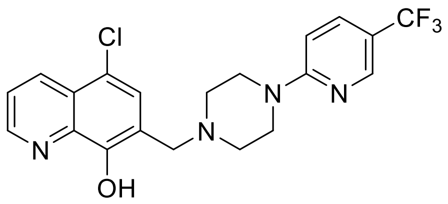

5-Chloro-7-((4-(5-(trifluoromethyl)pyridin-2-yl)piperazin-1-yl)methyl)quinolin-8-ol (**20**): 1-(5-(Trifluoromethyl)pyridin-2-yl)piperazine·TFA (0.0380 g, 0.111 mmol, 1 equiv), formaldehyde (37% wt in H_2O, 0.0130 g, 0.416 mmol, 3.75 equiv) and 5-chloro-8-quinolinol (0.0200 g, 0.111 mmol, 1 equiv) were reacted according to general procedure A to afford **20** (0.00960 g, 20%) as a white solid. Mp: 136–137 ˚C; TLC (5% MeOH/CH_2Cl_2): R_f = 0.5; ^{1}H NMR (500 MHz, $CDCl_3$) δ 8.91 (dd, *J* = 4.2, 1.6 Hz, 1H), 8.50 (dd, *J* = 8.5, 1.6 Hz, 1H), 8.43 – 8.38 (m, 1H), 7.64 (ddd, *J* = 9.1, 2.6, 0.7 Hz, 1H), 7.54 (dd, *J* = 8.5, 4.2 Hz, 1H), 7.46 (s, 1H), 6.64 (d, *J* = 9.1 Hz, 1H), 3.91 (s, 2H), 3.81 – 3.68 (m, 4H), 2.81 – 2.69 (m, 4H); ^{13}C NMR (126 MHz, $CDCl_3$) δ 160.3, 151.6, 149.4, 146.0, 145.9, 145.9, 145.8, 139.5, 134.8, 134.8, 134.8, 134.7, 133.1, 128.0, 126.2, 125.7, 123.6, 122.4, 120.4, 115.8, 115.5, 105.8, 58.9, 52.6, 44.7, 29.9; IR (neat) 3317, 1612 cm^{-1}; HRMS (ESI) *m/z* calcd for $C_{20}H_{19}ClF_3N_4O$ $[M + H]^+$: 423.1199, found: 423.1179; HPLC purity = 98.0%.

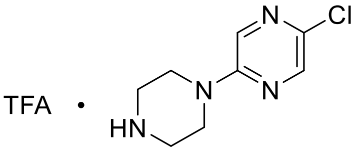

2-Chloro-5-(piperazin-1-yl)pyrazine·TFA (**S-21**): *N*-Boc-piperazine (0.482 g, 2.58 mmol, 1 equiv), 2-bromo-5-chloropyrazine (0.500 g, 2.58 mmol, 1 equiv), $Pd_2(dba)_3$ (0.0470 g, 0.052 mmol, 2 mole %), (±)-BINAP (0.064 g, 0.10 mmol, 4 mole %) and NaO^{t}Bu (0.373 g, 3.88 mmol, 1.5 equiv) were reacted according to general procedure I to afford Boc-protected **S-21** (0.26 g, 34%) as a yellow solid. TLC (20% Hexanes/EtOAc): R_f = 0.4; ^{1}H NMR (400 MHz, $CDCl_3$) δ δ 8.07 (d, *J* = 1.5 Hz, 1H), 7.86 (d, *J* = 1.4 Hz, 1H), 3.55 (s, 8H), 1.48 (s, 9H); ^{13}C NMR (101 MHz, $CDCl_3$) δ 154.8, 153.7, 141.2, 136.7, 129.4, 80.4, 44.7, 28.5. The boc-protected compound was reacted according to general procedure I to afford **S-21** (HRMS (ESI) *m/z* calcd for $C_{14}H_{23}N_4O_2$ $[M + H – CF_3COOH]^+$: 199.0750, found: 199.0750), which was used for next reaction without further purification.

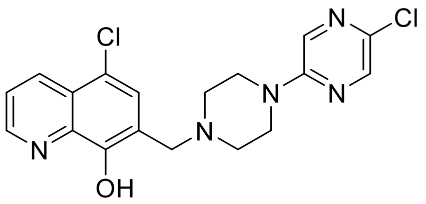

5-Chloro-7-((4-(5-chloropyrazin-2-yl)piperazin-1-yl)methyl)quinolin-8-ol (**21**): 2-Chloro-5-(piperazin-1-yl)pyrazine·TFA (0.0350 g, 0.111 mmol, 1 equiv), formaldehyde (37% wt in H_2O, 0.0130 g, 0.416 mmol, 3.75 equiv) and 5-chloro-8-quinolinol (0.0200 g, 0.111 mmol, 1 equiv) were reacted according to general procedure A to afford **21** (0.0148 g, 34%) as a white solid. Mp: 145–146 ˚C; TLC (5% MeOH/CH_2Cl_2): R_f = 0.6; ^{1}H NMR (500 MHz, $CDCl_3$) δ 8.90 (dd, *J* = 4.2, 1.6 Hz, 1H), 8.51 (dd, *J* = 8.6, 1.6 Hz, 1H), 8.07 (d, *J* = 1.4 Hz, 1H), 7.86 (d, *J* = 1.5 Hz, 1H), 7.54 (dd, *J* = 8.5, 4.2 Hz, 1H), 7.49 (s, 1H), 3.92 (s, 2H), 3.73 – 3.62 (m, 4H), 2.76 (s, 4H); ^{13}C NMR (126 MHz, $CDCl_3$) δ 153.6, 151.4, 149.3, 141.2, 139.4, 136.7, 133.2, 129.3, 128.1, 126.2, 122.5, 120.5, 58.5, 52.3, 44.7; IR (neat) 3298, 1567 cm^{-1}; HRMS (ESI) *m/z* calcd for $C_{18}H_{18}Cl_2N_5O$ [M + H]$^+$: 390.0888, found: 390.0876; HPLC purity = 96.8%.

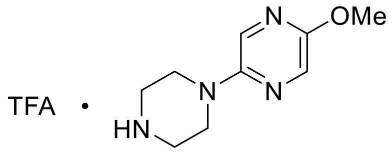

2-Methoxy-5-(piperazin-1-yl)pyrazine·TFA (**S-22**): *N*-Boc-piperazine (0.197 g, 1.06 mmol, 1 equiv), 2-bromo-5-methoxypyrazine (0.200 g, 1.06 mmol, 1 equiv), $Pd_2(dba)_3$ (0.0190 g, 0.021 mmol, 2 mole %), (±)-BINAP (0.026 g, 0.042 mmol, 4 mole %) and NaO^{t}Bu (0.152 g, 1.58 mmol, 1.5 equiv) were reacted according to general procedure I to afford Boc-protected **S-22** (0.080 g, 26%) as a white solid. TLC (20% Hexanes/EtOAc): R_f = 0.3; ^{1}H NMR (400 MHz, $CDCl_3$) δ 7.88 (d, *J* = 1.5 Hz, 1H), 7.63 (d, *J* = 1.5 Hz, 1H), 3.89 (s, 3H), 3.57 (dd, *J* = 6.4, 4.0 Hz, 4H), 3.39 – 3.35 (m, 4H), 1.48 (s, 9H); ^{13}C NMR (101 MHz, $CDCl_3$) δ 154.9, 154.8, 151.5, 131.3, 125.7, 80.2, 53.9, 46.5, 28.6. The boc-protected **S-22** was reacted according to general procedure I to afford **S-22** (HRMS (ESI) *m/z* calcd for $C_{11}H_{16}F_3N_4O_3$ [M + H – CF_3COOH]$^+$: 195.1246, found: 195.1247), which was used for next reaction without further purification.

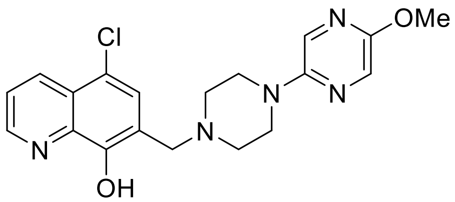

5-Chloro-7-((4-(5-methoxypyrazin-2-yl)piperazin-1-yl)methyl)quinolin-8-ol (**22**): 2-Methoxy-5-(piperazin-1-yl)pyrazine•TFA (0.0340 g, 0.111 mmol, 1 equiv), formaldehyde (37% wt in H_2O, 0.0130 g, 0.416 mmol, 3.75 equiv) and 5-chloro-8-quinolinol (0.0200 g, 0.111 mmol, 1 equiv) were reacted according to general procedure A to afford **22** (0.0103 g, 46%) as a light green solid. Mp: 139–141 ˚C; TLC (5% MeOH/CH_2Cl_2): R_f = 0.4; ^{1}H NMR (400 MHz, $CDCl_3$) δ 8.91 (dd, *J* = 4.2, 1.6 Hz, 1H), 8.50 (dd, *J* = 8.5, 1.6 Hz, 1H), 7.88 (d, *J* = 1.5 Hz, 1H), 7.63 (d, *J* = 1.5 Hz, 1H), 7.53 (dd, *J* = 8.5, 4.2 Hz, 1H), 7.46 (s, 1H), 3.96 (s, 2H), 3.89 (s, 3H), 3.59 – 3.47 (m, 4H), 2.83 (t, *J* = 4.8 Hz, 4H); ^{13}C NMR (101 MHz, $CDCl_3$) δ 154.8, 151.8, 151.3, 149.4, 139.6, 133.1, 131.3, 128.0, 126.3, 125.4, 122.4, 120.5, 53.8, 52.4, 46.2; IR (neat) 2838, 1539 cm^{-1}; HRMS (ESI) *m/z* calcd for $C_{19}H_{21}ClN_5O_2$ [M + H]$^+$: 386.1384, found: 386.1373; HPLC purity = 96.0%.

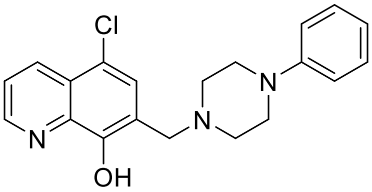

5-Chloro-7-((4-phenylpiperazin-1-yl)methyl)quinolin-8-ol (**23**). This compound was purchased (Enamine Ltd.) during our previous work on this series.[3]

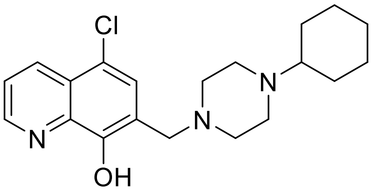

5-Chloro-7-((4-cyclohexylpiperazin-1-yl)methyl)quinolin-8-ol (**24**):

1-Cyclohexylpiperidine (0.0190 g, 0.111 mmol, 1 equiv), formaldehyde (37% wt in H_2O, 0.0130 g, 0.416 mmol, 3.75 equiv) and 5-chloro-8-quinolinol (0.0200 g, 0.111 mmol, 1 equiv) were reacted according to general procedure A to afford **24** (0.0337 g, 84%) as a white solid. Mp: 172–174 ˚C; TLC (5% MeOH/CH_2Cl_2): R_f = 0.3; ^{1}H NMR (500 MHz, $CDCl_3$) δ 8.92 (dd, *J* = 4.1, 1.7 Hz, 1H), 8.47 (dd, *J* = 8.5, 1.7 Hz, 1H), 7.49 (dd, *J* = 8.5,

4.1 Hz, 1H), 7.31 (s, 1H), 3.86 (s, 2H), 2.69 (s, 8H), 2.28 (s, 1H), 1.92 – 1.72 (m, 4H), 1.62 (dt, *J* = 13.3, 2.8 Hz, 1H), 1.33 – 1.01 (m, 6H); ^{13}C NMR (126 MHz, $CDCl_3$) δ 152.8, 149.5, 140.0, 132.9, 127.3, 126.2, 122.1, 119.9, 118.1, 63.6, 60.4, 53.2, 48.8, 29.0, 26.4, 26.0; IR (neat) 2930, 1496 cm^{-1}; HRMS (ESI) *m/z* calcd for $C_{20}H_{27}ClN_3O$ $[M + H]^+$: 360.1843, found: 360.1839; HPLC purity >99.8%.

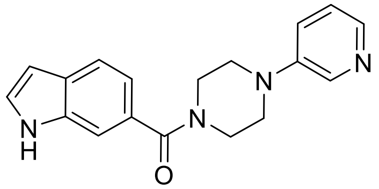

(1H-Indol-6-yl)(4-(pyridin-3-yl)piperazin-1-yl)methanone (**S-25**): Indole-6-carboxylic acid (0.0500 g, 0.31 mmol, 1 equiv), 1-pyridin-3-yl-piperazine (0.0510 g, 0.31 mmol, 1 equiv), HATU (0.118 g, 0.31 mmol, 1 equiv) and DIEA (0.080 g, 0.62 mmol, 2 equiv) were reacted according to general procedure G to afford **S-25** (0.091 g, 96%) as a brown solid. Mp: 185–187 ˚C; TLC (5% MeOH/CH_2Cl_2): R_f = 0.3; ^{1}H NMR (400 MHz, $CDCl_3$) δ 8.93 (s, 1H), 8.26 (t, *J* = 1.8 Hz, 1H), 8.13 (dd, *J* = 3.9, 2.3 Hz, 1H), 7.65 (d, *J* = 8.1 Hz, 1H), 7.58 (dt, *J* = 1.7, 0.9 Hz, 1H), 7.30 (dd, *J* = 3.2, 2.4 Hz, 1H), 7.25 (d, *J* = 2.5 Hz, 2H), 7.16 (dd, *J* = 8.2, 1.4 Hz, 1H), 6.57 (ddd, *J* = 3.0, 2.0, 0.9 Hz, 1H), 3.82 (s, 4H), 3.23 (s, 4H); ^{13}C NMR (101 MHz, $CDCl_3$) δ 172.0, 147.1, 140.1, 137.7, 135.5, 129.5, 128.7, 126.5, 124.1, 123.9, 120.7, 118.9, 111.2, 102.8, 48.9; IR (neat) 3201, 1509 cm^{-1}; HRMS (ESI) *m/z* calcd for $C_{18}H_{19}N_4O$ $[M + H]^+$: 307.1559, found: 307.1542.

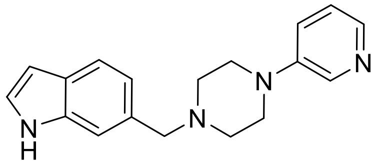

6-((4-(Pyridin-3-yl)piperazin-1-yl)methyl)-1H-indole (**25**): (1H-indol-6-yl)(4-(pyridin-3-yl)piperazin-1-yl)methanone (0.0250 g, 0.082 mmol, 1 equiv) and $LiAlH_4$ (2M in THF, 0.0093 g, 0.240, 3 equiv) were reacted according to general procedure F to afford **25** (0.0015 g, 6%) as a brown oil. TLC (5% MeOH/CH_2Cl_2): R_f = 0.4; ^{1}H NMR (500 MHz, $CDCl_3$) δ 8.42 (s, 1H), 8.29 (t, *J* = 1.8 Hz, 1H), 8.12 (dd, *J* = 3.3, 2.7 Hz, 1H), 7.71 – 7.59 (m, 2H), 7.17 (dd, *J* = 3.2, 1.7 Hz, 2H), 7.10 (dd, *J* = 8.1, 1.5 Hz, 1H), 6.56 (ddd, *J* = 3.1, 2.0, 0.9 Hz, 1H), 3.91 (s, 2H), 3.38 (t, *J* = 5.1 Hz, 4H), 2.86 (s, 4H); ^{13}C NMR (126 MHz, $CDCl_3$) δ 146.6, 141.4, 139.1, 136.1, 128.0, 125.3, 123.7, 122.9, 122.0, 120.8, 112.9,

103.6, 102.6, 63.1, 52.3, 47.8; HRMS (ESI) *m/z* calcd for $C_{18}H_{21}N_4$ $[M + H]^+$: 293.1766, found: 293.1757; HPLC purity = 94.2%.

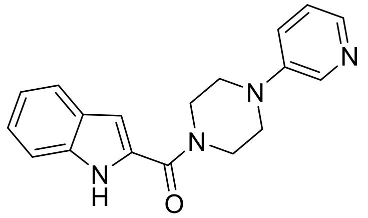

(1H-Indol-2-yl)(4-(pyridin-3-yl)piperazin-1-yl)methanone (**S-26**): Indole-2-carboxylic acid (0.0500 g, 0.31 mmol, 1 equiv), 1-pyridin-3-yl-piperazine (0.0510 g, 0.31 mmol, 1 equiv), HATU (0.118 g, 0.31 mmol, 1 equiv) and DIEA (0.0800 g, 0.62 mmol, 2 equiv) were reacted according to general procedure G to afford **S-26** (0.092 g, 97%) as a colorless oil. TLC (5% MeOH/CH_2Cl_2): R_f = 0.4; ^{1}H NMR (400 MHz, $CDCl_3$) δ 9.34 (s, 1H), 8.35 (t, *J* = 1.9 Hz, 1H), 8.17 (dd, *J* = 3.7, 2.3 Hz, 1H), 7.67 (dd, *J* = 8.0, 1.0 Hz, 1H), 7.44 (dd, *J* = 8.3, 1.0 Hz, 1H), 7.30 (ddd, *J* = 8.2, 7.0, 1.1 Hz, 1H), 7.25 – 7.20 (m, 2H), 7.16 (ddd, *J* = 8.0, 7.0, 1.0 Hz, 1H), 6.84 (dd, *J* = 2.1, 0.9 Hz, 1H), 4.12 (s, 4H), 3.40 – 3.28 (m, 4H); ^{13}C NMR (101 MHz, $CDCl_3$) δ 162.6, 146.7, 141.7, 139.2, 135.9, 129.1, 127.6, 124.8, 123.8, 123.0, 122.1, 120.9, 111.9, 105.6, 54.5, 49.0; IR (neat) 3258, 1586 cm^{-1}; HRMS (ESI) *m/z* calcd for $C_{18}H_{19}N_4O$ $[M + H]^+$: 307.1559, found: 307.1540.

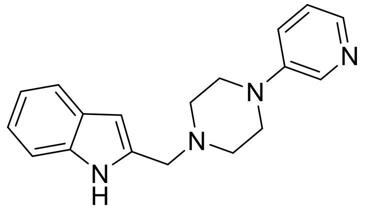

2-((4-(Pyridin-3-yl)piperazin-1-yl)methyl)-1H-indole (**26**): (1H-indol-2-yl)(4-(pyridin-3-yl)piperazin-1-yl)methanone (0.0250 g, 0.0816 mmol, 1 equiv) and $LiAlH_4$ (2M in THF, 0.0093 g, 0.244, 3 equiv) were reacted according to general procedure F to afford **26** (0.0053 g, 22%) as a brown oil. TLC (5% MeOH/CH_2Cl_2): R_f = 0.3; ^{1}H NMR (500 MHz, $CDCl_3$) δ 9.05 (s, 1H), 8.31 (t, *J* = 1.8 Hz, 1H), 8.13 (dd, *J* = 3.6, 2.4 Hz, 1H), 7.57 (dd, *J* = 7.9, 1.1 Hz, 1H), 7.39 (dd, *J* = 8.2, 1.0 Hz, 1H), 7.22 – 7.14 (m, 3H), 7.10 (ddd, *J* = 8.0, 7.1, 1.0 Hz, 1H), 6.44 (dd, *J* = 2.1, 1.0 Hz, 1H), 3.88 (s, 2H), 3.32 (t, *J* = 5.1 Hz, 4H), 2.79 (d, *J* = 5.0 Hz, 4H); ^{13}C NMR (126 MHz, $CDCl_3$) δ 146.6, 141.4, 139.0, 136.6, 128.1, 123.7, 122.9, 122.3, 120.5, 120.0, 111.2, 103.1, 55.6, 52.7, 48.2; IR (neat) 1454

cm^{-1}; HRMS (ESI) *m/z* calcd for $C_{18}H_{21}N_4$ $[M + H]^+$: 293.1766, found: 293.1743; HPLC purity > 99.8%.

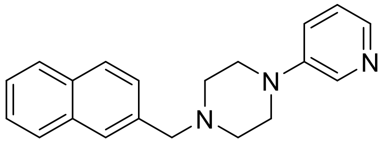

1-(Naphthalen-2-ylmethyl)-4-(pyridin-3-yl)piperazine (**27**): 2-(Bromomethyl)naphthalene (0.0200 g, 0.09 mmol, 1 equiv), 1-pyridyn-3-yl-piperazine (0.0160 g, 0.100 mmol, 1.1 equiv), KI (0.015 g, 0.09 mmol, 1 equiv) and triethylamine (0.0180 g, 0.18 mmol, 2 equiv) were reacted according to general procedure C to afford **27** (0.0022 g, 8%) as an oil. TLC (5% MeOH/CH_2Cl_2): $R_f = 0.4$; ^{1}H NMR (500 MHz, $CDCl_3$) δ 8.30 (d, *J* = 2.1 Hz, 1H), 8.10 (dd, *J* = 4.1, 2.0 Hz, 1H), 7.89 – 7.80 (m, 4H), 7.59 (dd, *J* = 8.5, 1.7 Hz, 1H), 7.53 – 7.46 (m, 2H), 7.19 (dt, *J* = 4.2, 1.5 Hz, 2H), 3.87 (s, 2H), 3.34 (t, *J* = 5.0 Hz, 4H), 2.80 (t, *J* = 5.0 Hz, 4H); ^{13}C NMR (126 MHz, $CDCl_3$) δ 146.9, 140.7, 138.5, 133.4, 133.2, 128.7, 128.5, 127.9, 127.8, 127.6, 126.4, 126.3, 123.8, 123.0, 62.9, 52.7, 48.1; IR (neat) 2821, 1582 cm^{-1}; HRMS (ESI) *m/z* calcd for $C_{20}H_{22}N_3$ $[M + H]^+$: 304.1814, found: 304.1817; HPLC purity > 99.8%.

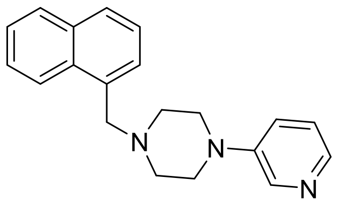

1-(Naphthalen-1-ylmethyl)-4-(pyridin-3-yl)piperazine (**28**): 2-(Chloromethyl)naphthalene (0.0200 g, 0.113 mmol, 1 equiv), 1-pyridyn-3-yl-piperazine (0.0200 g, 0.120 mmol, 1.1 equiv), KI (0.0190 g, 0.113 mmol, 1 equiv) and triethylamine (0.0230 g, 0.23 mmol, 2 equiv) were reacted according to general procedure C to afford **28** (0.0126 g, 37%) as an brown oil. TLC (5% MeOH/CH_2Cl_2): $R_f = 0.6$; ^{1}H NMR (500 MHz, $CDCl_3$) δ 8.35 – 8.24 (m, 2H), 8.08 (s, 1H), 7.91 – 7.85 (m, 1H), 7.82 (d, *J* = 8.4 Hz, 1H), 7.58 – 7.47 (m, 3H), 7.44 (dd, *J* = 8.2, 7.0 Hz, 1H), 7.23 – 7.15 (m, 2H), 4.06 (s, 2H), 3.35 – 3.18 (m, 4H), 2.75 (t, *J* = 5.0 Hz, 4H); ^{13}C NMR (126 MHz, $CDCl_3$) δ 147.1, 140.0, 137.9, 134.0, 132.6, 128.7, 128.6, 128.2, 126.2, 125.9, 125.3, 124.6, 123.9, 122.9, 60.8, 52.9, 48.3; IR (neat) 2823, 1583 cm^{-1}; HRMS (ESI) *m/z* calcd for $C_{20}H_{22}N_3$ $[M + H]^+$: 304.1814, found: 304.1869; HPLC purity = 94.4%.

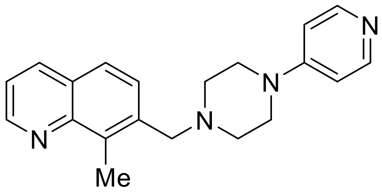

8-Methyl-7-((4-(pyridin-4-yl)piperazin-1-yl)methyl)quinolone (**29**): (8-Methylquinolin-7-yl)methanol (0.0200 g, 0.115 mmol, 1 equiv), methanesulfonyl chloride (0.0250 g, 0.173 mmol, 1.5 equiv) and triethylamine (0.0180, 0.173 mmol, 1.5 equiv) were reacted according to general procedure C to give the mesylate intermediate, which was reacted with 1-(4-pyridyl)piperazine (0.0230 g, 0.138 mmol, 1.2 equiv) according to general procedure C to afford **29** (0.0350 g, 67%) as a yellow oil. TLC (5% MeOH/CH_2Cl_2): R_f = 0.5; ^{1}H NMR (500 MHz, $CDCl_3$) δ 8.96 (dd, *J* = 4.2, 1.8 Hz, 1H), 8.29 – 8.23 (m, 2H), 8.13 (dd, *J* = 8.2, 1.9 Hz, 1H), 7.65 (d, *J* = 8.4 Hz, 1H), 7.59 (d, *J* = 8.4 Hz, 1H), 7.40 (dd, *J* = 8.2, 4.2 Hz, 1H), 6.69 – 6.63 (m, 2H), 3.78 (s, 2H), 3.39 – 3.32 (m, 4H), 2.87 (s, 3H), 2.66 – 2.60 (m, 4H); ^{13}C NMR (126 MHz, $CDCl_3$) δ 155.2, 149.7, 149.4, 147.6, 136.7, 136.3, 136.2, 128.9, 127.6, 125.1, 120.8, 108.4, 60.9, 52.8, 46.2, 13.2; IR (neat) 1595 cm^{-1}; HRMS (ESI) *m/z* calcd for $C_{20}H_{23}N_4$ [M + H]$^+$: 319.1923, found: 319.1909; HPLC purity = 100%.

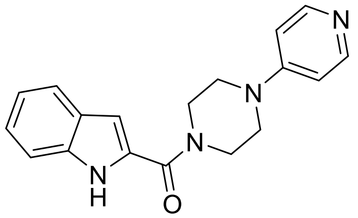

(1H-Indol-2-yl)(4-(pyridin-4-yl)piperazin-1-yl)methanone (**S-30**): Indole-2-carboxylic acid (0.0500 g, 0.31 mmol, 1 equiv), 1-(4-pyridyl)piperazine (0.0510 g, 0.31 mmol, 1 equiv), HATU (0.118 g, 0.31 mmol, 1 equiv) and DIEA (0.0800 g, 0.62 mmol, 2 equiv) were reacted according to general procedure G to afford **S-30** (0.094 g, 99%) as a white solid. Mp: 205–207 ˚C; TLC (5% MeOH/CH_2Cl_2): R_f = 0.2; ^{1}H NMR (400 MHz, $CDCl_3$) δ 9.35 (s, 1H), 8.39 – 8.29 (m, 2H), 7.67 (dd, *J* = 8.1, 1.0 Hz, 1H), 7.45 (dd, *J* = 8.3, 1.0 Hz, 1H), 7.31 (ddd, *J* = 8.2, 7.0, 1.1 Hz, 1H), 7.16 (ddd, *J* = 8.0, 7.0, 1.0 Hz, 1H), 6.84 (dd, *J* = 2.2, 0.9 Hz, 1H), 6.72 – 6.64 (m, 2H), 4.11 (dt, *J* = 7.2, 3.5 Hz, 4H), 3.56 – 3.45 (m, 4H); ^{13}C NMR (101 MHz, $CDCl_3$) δ 162.7, 154.6, 150.6, 135.9, 128.9, 127.7, 124.9,

122.1, 121.0, 111.9, 108.5, 105.7, 60.5, 45.9; IR (neat) 3235, 1592 cm^{-1}; HRMS (ESI) *m/z* calcd for $C_{18}H_{19}N_4O$ $[M + H]^+$: 307.1559, found: 307.1538.

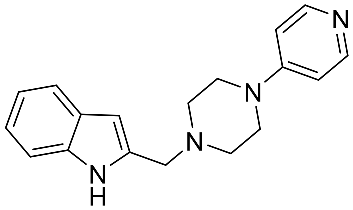

2-((4-(Pyridin-4-yl)piperazin-1-yl)methyl)-1H-indole (**30**): (1H-indol-2-yl)(4-(pyridin-4-yl)piperazin-1-yl)methanone **S-30** (0.0300 g, 0.098 mmol, 1 equiv) and $LiAlH_4$ (2M in THF, 0.0110 g, 0.294, 3 equiv) were reacted according to general procedure F to afford **30** (0.0058 g, 20%) as a yellow solid. Mp: 175–177 ˚C; TLC (5% MeOH/CH_2Cl_2): R_f = 0.2; ^{1}H NMR (500 MHz, $CDCl_3$) δ 8.49 (s, 1H), 8.21 (d, *J* = 7.3 Hz, 2H), 7.57 (dd, *J* = 7.9, 1.1 Hz, 1H), 7.36 (dd, *J* = 8.1, 0.9 Hz, 1H), 7.18 (ddd, *J* = 8.2, 7.1, 1.2 Hz, 1H), 7.10 (ddd, *J* = 8.0, 7.1, 1.0 Hz, 1H), 6.78 – 6.70 (m, 2H), 6.43 – 6.38 (m, 1H), 3.75 (s, 2H), 3.52 – 3.45 (m, 5H), 2.68 – 2.63 (m, 4H); ^{13}C NMR (126 MHz, $CDCl_3$) δ 156.0, 145.4, 136.4, 134.5, 128.3, 122.1, 120.5, 120.0, 110.9, 107.9, 102.5, 55.7, 52.4, 46.1; IR (neat) 2825, 1594 cm^{-1}; HRMS (ESI) *m/z* calcd for $C_{18}H_{21}N_4$ $[M + H]^+$: 293.1766, found: 293.1735; HPLC purity > 99.8%.

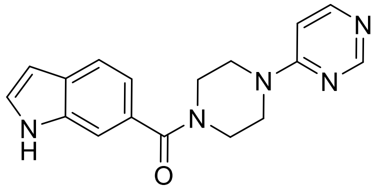

(1H-Indol-6-yl)(4-(pyrimidin-4-yl)piperazin-1-yl)methanone (**S-31**): Indole-6-carboxylic acid (0.0500 g, 0.31 mmol, 1 equiv), 4-(piperazin-1-yl)pyrimidine•2HCl (0.0740 g, 0.31 mmol, 1 equiv), HATU (0.118 g, 0.31 mmol, 1 equiv) and DIEA (0.120 g, 0.93 mmol, 3 equiv) were reacted according to general procedure G to afford **S-31** (0.091 g, 96%) as a brown solid. Mp: 227–229 ˚C; TLC (5% MeOH/CH_2Cl_2): R_f = 0.3; ^{1}H NMR (400 MHz, $CDCl_3$) δ 8.68 (s, 1H), 8.62 (s, 1H), 8.24 (d, *J* = 6.3 Hz, 1H), 7.66 (d, *J* = 8.2 Hz, 1H), 7.58 (s, 1H), 7.32 (t, *J* = 2.8 Hz, 1H), 7.17 (dd, *J* = 8.1, 1.4 Hz, 1H), 6.59 (d, *J* = 2.7 Hz, 1H), 6.54 (dd, *J* = 6.4, 1.2 Hz, 1H), 3.72 (s, 8H); ^{13}C NMR (101 MHz, $CDCl_3$) δ 172.1, 161.5, 157.9, 155.1, 135.5, 129.6, 128.6, 126.5, 120.8, 119.0,

111.2, 103.2, 103.0, 44.0; IR (neat) 3260, 1584 cm^{-1}; HRMS (ESI) *m/z* calcd for $C_{17}H_{18}N_5O$ $[M + H]^+$: 308.1511, found: 308.1506.

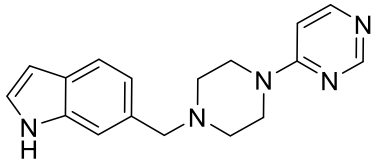

6-((4-(Pyrimidin-4-yl)piperazin-1-yl)methyl)-1H-indole (**31**): (1H-indol-6-yl)(4-(pyrimidin-4-yl)piperazin-1-yl)methanone **S-31** (0.0200 g, 0.0650 mmol, 1 equiv) and $LiAlH_4$ (2M in THF, 0.0740 g, 0.195, 3 equiv) were reacted according to general procedure F to afford **31** (0.0022 g, 12%) as a brown oil. TLC (5% MeOH/CH_2Cl_2): R_f = 0.4; ^{1}H NMR (500 MHz, $CDCl_3$) δ 8.58 (dd, *J* = 1.3, 0.6 Hz, 1H), 8.28 (s, 1H), 8.19 (dd, *J* = 6.3, 0.7 Hz, 1H), 7.61 (d, *J* = 8.0 Hz, 1H), 7.49 (s, 1H), 7.23 (dd, *J* = 3.2, 2.3 Hz, 1H), 7.09 (dd, *J* = 8.1, 1.5 Hz, 1H), 6.55 (td, *J* = 2.1, 1.0 Hz, 1H), 6.48 (dd, *J* = 6.4, 1.3 Hz, 1H), 3.76 (s, 2H), 3.73 (s, 4H), 2.64 (t, *J* = 5.2 Hz, 3H); ^{13}C NMR (126 MHz, $CDCl_3$) δ 161.3, 158.4, 155.7, 136.1, 127.6, 124.9, 121.8, 121.2, 120.7, 112.2, 103.1, 102.7, 63.5, 52.5, 43.4; HRMS (ESI) *m/z* calcd for $C_{17}H_{20}N_5$ $[M + H]^+$: 294.1719, found: 294.1715; HPLC purity > 99.8%.

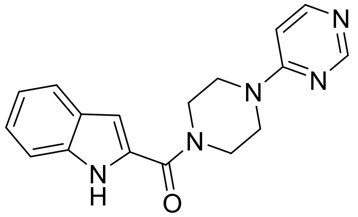

(1H-Indol-2-yl)(4-(pyrimidin-4-yl)piperazin-1-yl)methanone (**S-32**): Indole-2-carboxylic acid (0.0500 g, 0.31 mmol, 1 equiv), 4-(piperazin-1-yl)pyrimidine·2HCl (0.0740 g, 0.31 mmol, 1 equiv), HATU (0.118 g, 0.31 mmol, 1 equiv) and DIEA (0.120 g, 0.93 mmol, 3 equiv) were reacted according to general procedure G to afford **S-32** (0.070 g, 73%) as a white solid. Mp: 187–188 ˚C; TLC (5% MeOH/CH_2Cl_2): R_f = 0.4; ^{1}H NMR (400 MHz, $CDCl_3$) δ 9.60 (s, 1H), 8.66 (s, 1H), 8.28 (d, *J* = 6.2 Hz, 1H), 7.67 (dd, *J* = 8.0, 1.1 Hz, 1H), 7.45 (dd, *J* = 8.3, 1.0 Hz, 1H), 7.30 (ddd, *J* = 8.2, 7.0, 1.1 Hz, 1H), 7.15 (ddd, *J* = 8.0, 6.9, 1.0 Hz, 1H), 6.83 (dd, *J* = 2.2, 0.9 Hz, 1H), 6.54 (dd, *J* = 6.3, 1.2 Hz, 1H), 4.08 (s, 4H), 3.92 – 3.71 (m, 4H); ^{13}C NMR (101 MHz, $CDCl_3$) δ 162.9, 161.4, 158.5, 156.1, 135.9, 128.9, 127.6, 124.9, 122.1, 120.9, 111.9, 105.8, 103.1, 43.5; IR

(neat) 3283, 1588 cm^{-1}; HRMS (ESI) *m/z* calcd for $C_{17}H_{18}N_5O$ [M + H]$^+$: 308.1511, found: 308.1501.

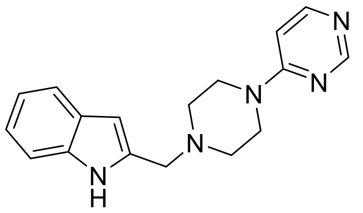

2-((4-(Pyrimidin-4-yl)piperazin-1-yl)methyl)-1H-indole (**32**): (1H-indol-2-yl)(4-(pyrimidin-4-yl)piperazin-1-yl)methanone **S-32** (0.0200 g, 0.065 mmol, 1 equiv) and $LiAlH_4$ (2M in THF, 0.0074 g, 0.195, 3 equiv) were reacted according to general procedure F to afford **32** (0.004 g, 21%) as a colorless oil. TLC (5% MeOH/CH_2Cl_2): R_f = 0.3; ^{1}H NMR (500 MHz, $CDCl_3$) δ 8.65 (s, 1H), 8.60 (dd, *J* = 1.2, 0.6 Hz, 1H), 8.20 (dd, *J* = 6.3, 0.6 Hz, 1H), 7.57 (dd, *J* = 7.9, 1.0 Hz, 1H), 7.37 (dd, *J* = 8.1, 0.9 Hz, 1H), 7.18 (ddd, *J* = 8.2, 7.1, 1.3 Hz, 1H), 7.10 (ddd, *J* = 8.0, 7.1, 1.1 Hz, 1H), 6.49 (dd, *J* = 6.3, 1.3 Hz, 1H), 6.39 (dd, *J* = 2.0, 0.9 Hz, 1H), 3.75 (d, *J* = 0.8 Hz, 2H), 3.69 (t, *J* = 5.1 Hz, 4H), 2.59 (t, *J* = 5.2 Hz, 4H); ^{13}C NMR (126 MHz, $CDCl_3$) δ 161.4, 158.5, 155.8, 136.4, 134.5, 128.3, 122.0, 120.4, 119.9, 111.0, 103.1, 102.4, 55.9, 52.7, 43.6; IR (neat) 3201, 1592 cm^{-1}; HRMS (ESI) *m/z* calcd for $C_{17}H_{20}N_5$ [M + H]$^+$: 294.1719, found: 294.1706; HPLC purity > 99.8%.

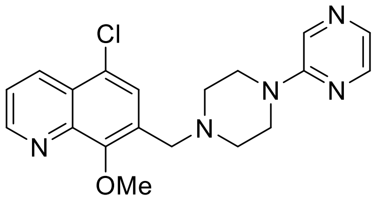

5-Chloro-8-methoxy-7-((4-(pyrazin-2-yl)piperazin-1-yl)methyl)quinolone (**33**): To a solution of 5-Chloro-7-((4-(pyrazin-2-yl)piperazin-1-yl)methyl)quinolin-8-ol **18** (0.0500 g, 0.140 mmol, 1 equiv) in DMF (5 mL), was added NaH (60% dispersion in mineral oil, 0.009 g, 0.210 mmol, 1.5 equiv) at 0 ˚C and stirred for 30 mins. Then, methyl iodide (0.019 g, 0.140 mmol, 1 equiv) was added to reaction mixture at 0 ˚C and the mixture stirred at rt for 4 additional h. The mixture was quenched by adding water (10 mL) and the aqueous layer was extracted with EtOAc (3 × 10 mL). The organic layers were combined, dried (Na_2SO_4) and concentrated in vacuo. The crude product was purified by mass directed

HPLC to afford **33** (0.0102 g, 19%) as a yellow oil. TLC (5% MeOH/CH_2Cl_2): R_f = 0.5; ^{1}H NMR (500 MHz, $CDCl_3$) δ 8.98 (dd, *J* = 4.2, 1.7 Hz, 1H), 8.55 (dd, *J* = 8.5, 1.7 Hz, 1H), 8.13 (d, *J* = 1.5 Hz, 1H), 8.06 (dd, *J* = 2.6, 1.5 Hz, 1H), 7.84 (d, *J* = 2.6 Hz, 1H), 7.80 (s, 1H), 7.52 (dd, *J* = 8.5, 4.2 Hz, 1H), 4.13 (s, 3H), 3.83 (s, 2H), 3.63 (t, *J* = 5.1 Hz, 4H), 2.67 (t, *J* = 5.0 Hz, 4H); ^{13}C NMR (126 MHz, $CDCl_3$) δ 155.1, 153.7, 150.2, 149.4, 143.5, 141.9, 133.4, 133.1, 131.2, 128.4, 127.0, 126.1, 122.0, 63.0, 56.1, 52.9, 44.6; IR (neat) 2837, 1575 cm^{-1}; HRMS (ESI) *m/z* calcd for $C_{19}H_{21}ClN_5O$ $[M + H]^+$: 370.1435, found: 370.1440; HPLC purity > 99.8%.

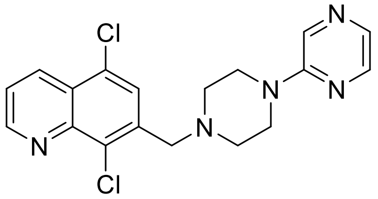

5,8-Dichloro-7-((4-(pyrazin-2-yl)piperazin-1-yl)methyl)quinolone (**34**): (5,8-Dichloroquinolin-7-yl)methanol (0.0200 g, 0.0877 mmol, 1 equiv), methanesulfonyl chloride (0.0190 g, 0.132 mmol, 1.5 equiv) and triethylamine (0.0133, 0.132 mmol, 1.5 equiv) were reacted according to general procedure D to afford the mesylated intermediate, which was reacted with 1-(2-pyrazinyl)piperazine (0.0173 g, 0.105 mmol, 1.2 equiv) according to general procedure D to afford **34** (0.0154 g, 47%) as a white solid. Mp: 178–179 ˚C; TLC (5% MeOH/CH_2Cl_2): R_f = 0.6; ^{1}H NMR (500 MHz, $CDCl_3$) δ 9.10 (dd, *J* = 4.2, 1.7 Hz, 1H), 8.61 (dd, *J* = 8.5, 1.7 Hz, 1H), 8.15 (d, *J* = 1.6 Hz, 1H), 8.07 (dd, *J* = 2.7, 1.5 Hz, 1H), 7.94 (s, 1H), 7.86 (d, *J* = 1.9 Hz, 1H), 7.60 (dd, *J* = 8.5, 4.1 Hz, 1H), 3.95 (s, 2H), 3.65 (d, *J* = 5.4 Hz, 4H), 2.70 (t, *J* = 4.8 Hz, 4H); ^{13}C NMR (126 MHz, $CDCl_3$) δ 155.1, 151.6, 145.3, 141.9, 137.9, 133.6, 133.2, 131.8, 131.2, 130.1, 128.0, 126.7, 122.5, 59.4, 53.0, 44.7; IR (neat) 2838, 1575 cm^{-1}; HRMS (ESI) *m/z* calcd for $C_{18}H_{18}Cl_2N_4$ $[M + H]^+$: 374.0939, found: 374.0947; HPLC purity > 99.8%.

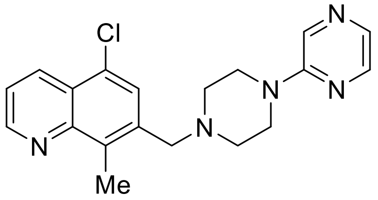

5-Chloro-8-methyl-7-((4-(pyrazin-2-yl)piperazin-1-yl)methyl)quinolone (**35**): (5-Chloro-8-methylquinolin-7-yl)methanol (0.0100 g, 0.0481 mmol, 1 equiv),

methanesulfonyl chloride (0.0110 g, 0.072 mmol, 1.5 equiv) and triethylamine (0.00800, 0.072 mmol, 1.5 equiv) were reacted according to general procedure D to afford the mesylated intermediate, which was reacted with 1-(2-pyrazinyl)piperazine (0.0100 g, 0.058 mmol, 1.2 equiv) according to general procedure D to afford **35** (0.0085 g, 50%) as a white solid. Mp: 163–165 ˚C; TLC (5% MeOH/CH_2Cl_2): $R_f = 0.6$; ^{1}H NMR (400 MHz, $CDCl_3$) δ 8.98 (dd, J = 4.2, 1.8 Hz, 1H), 8.56 (dd, J = 8.5, 1.8 Hz, 1H), 8.13 (d, J = 1.6 Hz, 1H), 8.06 (dd, J = 2.6, 1.5 Hz, 1H), 7.85 (d, J = 2.6 Hz, 1H), 7.76 (s, 1H), 7.50 (dd, J = 8.5, 4.2 Hz, 1H), 3.76 (s, 2H), 3.63 (s, 4H), 2.83 (s, 3H), 2.65 (s, 4H); IR (neat) 2839, 1582 cm^{-1}; HRMS (ESI) *m/z* calcd for $C_{20}H_{21}ClN_5$ [M + H]$^+$: 354.1485, found: 354.1456; HPLC purity > 99.8%.

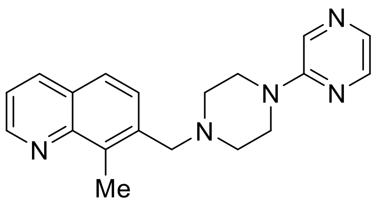

8-Methyl-7-((4-(pyrazin-2-yl)piperazin-1-yl)methyl)quinolone (**36**): (8-Methylquinolin-7-yl)methanol (0.0200 g, 0.115 mmol, 1 equiv), methanesulfonyl chloride (0.0250 g, 0.173 mmol, 1.5 equiv) and triethylamine (0.0180, 0.173 mmol, 1.5 equiv) were reacted according to general procedure D to afford the mesylated intermediate, which was reacted with 1-(2-pyrazinyl)piperazine (0.0230 g, 0.138 mmol, 1.2 equiv) according to general procedure D to afford **36** (0.0350 g, 67%) as a white solid. Mp: 126–128 ˚C; TLC (5% MeOH/CH_2Cl_2): $R_f = 0.6$; ^{1}H NMR (500 MHz, $CDCl_3$) δ 8.96 (dd, J = 4.2, 1.8 Hz, 1H), 8.15 – 8.11 (m, 2H), 8.05 (dd, J = 2.6, 1.5 Hz, 1H), 7.84 (d, J = 2.6 Hz, 1H), 7.66 (d, J = 8.3 Hz, 1H), 7.60 (d, J = 8.5 Hz, 1H), 7.39 (dd, J = 8.2, 4.2 Hz, 1H), 3.78 (s, 2H), 3.60 (t, J = 5.1 Hz, 4H), 2.88 (s, 3H), 2.68 – 2.59 (m, 4H); ^{13}C NMR (126 MHz, $CDCl_3$) δ 155.2, 149.4, 147.6, 141.9, 136.9, 136.3, 136.2, 133.0, 131.2, 128.9, 127.6, 125.1, 120.7, 61.0, 52.9, 44.7, 13.2; IR (neat) 2925, 1575 cm^{-1}; HRMS (ESI) *m/z* calcd for $C_{19}H_{22}N_5$ [M + H]$^+$: 320.1875, found: 320.1843; HPLC purity > 99.8%.

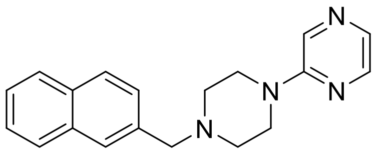

2-(4-(Naphthalen-2-ylmethyl)piperazin-1-yl)pyrazine (37): 2-(Bromomethyl)naphthalene (0.0200 g, 0.09 mmol, 1 equiv), 1-(2-pyrazinyl)piperazine (0.0160 g, 0.100 mmol, 1.1 equiv), KI (0.015 g, 0.09 mmol, 1 equiv) and triethylamine (0.0180 g, 0.18 mmol, 2 equiv) were reacted according to general procedure C to afford **37** (0.0122 g, 45%) as a white solid. Mp: 130–131 ˚C; TLC (5% MeOH/CH_2Cl_2): R_f = 0.5; ^{1}H NMR (400 MHz, $CDCl_3$) δ 8.12 (d, *J* = 1.6 Hz, 1H), 8.05 (dd, *J* = 2.6, 1.5 Hz, 1H), 7.87 – 7.80 (m, 4H), 7.78 – 7.74 (m, 1H), 7.53 (dd, *J* = 8.4, 1.7 Hz, 1H), 7.49 – 7.44 (m, 2H), 3.72 (s, 2H), 3.66 – 3.55 (m, 4H), 2.66 – 2.54 (m, 4H); ^{13}C NMR (101 MHz, $CDCl_3$) δ 155.2, 141.9, 135.6, 133.5, 133.0, 133.0, 131.2, 128.2, 127.8, 127.8, 127.5, 126.2, 125.9, 63.4, 52.9, 44.7; IR (neat) 2835, 1579 cm^{-1}; HRMS (ESI) *m/z* calcd for $C_{19}H_{21}N_4$ $[M + H]^+$: 305.1766, found: 305.1769; HPLC purity > 99.8%.

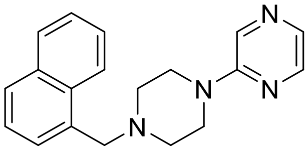

2-(4-(Naphthalen-1-ylmethyl)piperazin-1-yl)pyrazine (38): -(Chloromethyl)naphthalene (0.0200 g, 0.11 mmol, 1 equiv), 1-(2-pyrazinyl)piperazine (0.0200 g, 0.12 mmol, 1.1 equiv), KI (0.019 g, 0.11 mmol, 1 equiv) and triethylamine (0.0230 g, 0.23 mmol, 2 equiv) were reacted according to general procedure C to afford **38** (0.0113 g, 34%) as a tan solid. Mp: 152–154 ˚C; HRMS (ESI) *m/z* calcd for $C_{19}H_{21}N_4$ $[M + H]^+$: 305.1706, found: 305.1815; HPLC purity = 99.6%

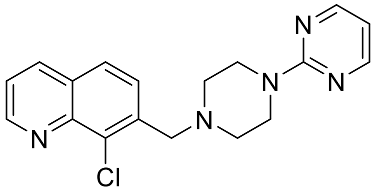

8-Chloro-7-((4-(pyrimidin-2-yl)piperazin-1-yl)methyl)quinolone (39): (8-Chloroquinolin-7-yl)methanol (0.0200 g, 0.126 mmol, 1 equiv), methanesulfonyl chloride (0.0270 g, 0.190 mmol, 1.5 equiv) and triethylamine (0.0192, 0.190 mmol, 1.5 equiv) were reacted according to general procedure D to afford the mesylated intermediate, which was reacted with 1-(2-pyrimidyl)piperazine (0.0250 g, 0.152 mmol, 1.2 equiv) according to general procedure D to afford **39** (0.0202 g, 47%) as a white solid. Mp: 179–181 ˚C; TLC (5% MeOH/CH_2Cl_2): R_f = 0.6; ^{1}H NMR (500 MHz, $CDCl_3$) δ 9.06 (dd, *J* = 4.3, 1.7 Hz,

1H), 8.23 – 8.15 (m, 2H), 7.85 (s, 1H), 7.77 (d, *J* = 8.3 Hz, 1H), 7.52 – 7.41 (m, 2H), 6.69 – 6.57 (m, 2H), 4.00 (s, 2H), 3.60 (s, 4H), 2.72 (s, 4H); ^{13}C NMR (126 MHz, $CDCl_3$) δ 159.5, 151.1, 148.0, 144.8, 137.7, 136.5, 132.9, 128.5, 126.3, 121.8, 113.5, 111.9, 107.3, 59.7, 53.2, 45.3; IR (neat) 1593, 1437 cm^{-1}; HRMS (ESI) *m/z* calcd for $C_{19}H_{20}ClN_4$ [M + H]$^+$: 339.1376, found: 339.1368; HPLC purity > 99.8%.

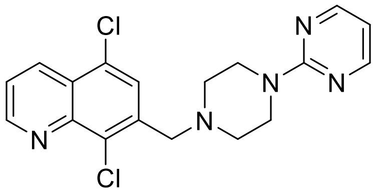

5,8-Dichloro-7-((4-(pyrimidin-2-yl)piperazin-1-yl)methyl)quinolone (**40**): (5,8-Dichloroquinolin-7-yl)methanol (0.0200 g, 0.0877 mmol, 1 equiv), methanesulfonyl chloride (0.0190 g, 0.132 mmol, 1.5 equiv) and triethylamine (0.0133, 0.132 mmol, 1.5 equiv) were reacted according to general procedure D to afford the mesylated intermediate, which was reacted with 1-(2-pyrimidyl)piperazine (0.0173 g, 0.105 mmol, 1.2 equiv) according to general procedure D to afford the **40** (0.0144 g, 44%) as a white solid. Mp: 158–159 ˚C; TLC (5% MeOH/CH_2Cl_2): R_f = 0.7; ^{1}H NMR (500 MHz, $CDCl_3$) δ 9.09 (dd, *J* = 4.2, 1.7 Hz, 1H), 8.60 (dd, *J* = 8.6, 1.7 Hz, 1H), 8.31 (d, *J* = 4.7 Hz, 2H), 7.96 (s, 1H), 7.59 (dd, *J* = 8.5, 4.2 Hz, 1H), 6.49 (t, *J* = 4.7 Hz, 1H), 3.93 (s, 2H), 3.89 (t, *J* = 5.2 Hz, 4H), 2.64 (d, *J* = 4.5 Hz, 4H); ^{13}C NMR (126 MHz, $CDCl_3$) δ 161.8, 157.9, 151.5, 145.3, 138.2, 133.6, 131.8, 130.1, 128.1, 126.6, 122.5, 110.0, 59.5, 53.3, 43.9; IR (neat) 1585, 1547 cm^{-1}; HRMS (ESI) *m/z* calcd for $C_{18}H_{18}Cl_2N_4$ [M + H]$^+$: 374.0939, found: 374.0940; HPLC purity > 99.8%.

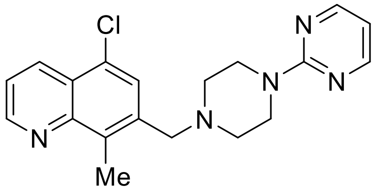

5-Chloro-8-methyl-7-((4-(pyrimidin-2-yl)piperazin-1-yl)methyl)quinolone (**41**): (5-Chloro-8-methylquinolin-7-yl)methanol (0.0100 g, 0.0481 mmol, 1 equiv), methanesulfonyl chloride (0.0110 g, 0.072 mmol, 1.5 equiv) and triethylamine (0.00800, 0.072 mmol, 1.5 equiv) were reacted according to general procedure D to afford the mesylated intermediate, which was reacted with 1-(2-pyrimidyl)piperazine (0.0100 g,

0.058 mmol, 1.2 equiv) according to general procedure D to afford **41** (0.0089 g, 52%) as a white solid. Mp: 128–130 ˚C; TLC (5% MeOH/CH_2Cl_2): R_f = 0.7; ^{1}H NMR (500 MHz, $CDCl_3$) δ 8.98 (dd, *J* = 4.1, 1.8 Hz, 1H), 8.56 (dd, *J* = 8.5, 1.7 Hz, 1H), 8.31 (d, *J* = 4.7 Hz, 2H), 7.76 (s, 1H), 7.56 – 7.43 (m, 1H), 6.49 (s, 1H), 3.85 (s, 4H), 3.73 (s, 2H), 2.83 (s, 3H), 2.58 (s, 4H); ^{13}C NMR (126 MHz, $CDCl_3$) δ 161.8, 157.9, 149.8, 148.2, 137.5, 135.3, 133.1, 128.5, 125.5, 121.4, 110.0, 105.2, 60.5, 53.3, 43.9, 13.2; IR (neat) 2945, 1589 cm^{-1}; HRMS (ESI) *m/z* calcd for $C_{20}H_{21}ClN_5$ $[M + H]^+$: 354.1485, found: 354.1470; HPLC purity > 99.8%.

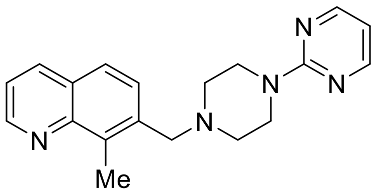

8-Methyl-7-((4-(pyrimidin-2-yl)piperazin-1-yl)methyl)quinolone (**42**): (8-Methylquinolin-7-yl)methanol (0.0280 g, 0.160 mmol, 1 equiv), methanesulfonyl chloride (0.0350 g, 0.242 mmol, 1.5 equiv) and triethylamine (0.0245, 0.242 mmol, 1.5 equiv) were reacted according to general procedure D to afford the mesylated intermediate, which was reacted with 1-(2-pyrimidyl)piperazine (0.0315 g, 0.192 mmol, 1.2 equiv) were reacted according to general procedure D to afford **42** (0.0350 g, 67%) as a brown solid. Mp: 118–120 ˚C; TLC (5% MeOH/CH_2Cl_2): R_f = 0.6; ^{1}H NMR (500 MHz, $CDCl_3$) δ 8.96 (dd, *J* = 4.2, 1.8 Hz, 1H), 8.30 (d, *J* = 4.7 Hz, 2H), 8.13 (dd, *J* = 8.2, 1.8 Hz, 1H), 7.66 (d, *J* = 11.9 Hz, 2H), 7.41 (dd, *J* = 8.2, 4.2 Hz, 1H), 6.50 (t, *J* = 4.8 Hz, 1H), 3.84 (s, 4H), 2.88 (s, 3H), 2.62 (s, 4H); ^{13}C NMR (126 MHz, $CDCl_3$) δ 161.6, 157.9, 149.5, 147.5, 139.2, 136.4, 129.0, 127.8, 125.4, 121.0, 110.2, 53.0, 41.1, 29.9, 13.4; IR (neat) 2930, 1585 cm^{-1}; HRMS (ESI) *m/z* calcd for $C_{19}H_{22}N_5$ $[M + H]^+$: 320.1875, found: 320.1874; HPLC purity > 99.8%.

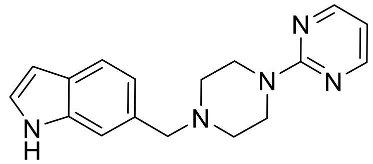

6-((4-(Pyrimidin-2-yl)piperazin-1-yl)methyl)-1H-indole (**43**): (1H-indol-6-yl)(4-(pyrimidin-2-yl)piperazin-1-yl)methanone **48** (0.0300 g, 0.0976 mmol, 1 equiv) and $LiAlH_4$ (2M in THF, 0.0110 g, 0.292, 3 equiv) were reacted according to general procedure F to afford **43** (0.0086 g, 30%) as a colorless oil; TLC (5% MeOH/CH_2Cl_2): R_f

= 0.4; ^{1}H NMR (500 MHz, $CDCl_3$) δ 8.29 (d, *J* = 4.8 Hz, 2H), 8.26 (s, 1H), 7.59 (d, *J* = 8.1 Hz, 1H), 7.42 – 7.37 (m, 1H), 7.20 (dd, *J* = 3.2, 2.4 Hz, 1H), 7.11 (dd, *J* = 8.2, 1.4 Hz, 1H), 6.54 (ddd, *J* = 3.1, 2.0, 1.0 Hz, 1H), 6.46 (t, *J* = 4.7 Hz, 1H), 3.83 (t, *J* = 5.2 Hz, 4H), 3.67 (s, 2H), 2.59 – 2.49 (m, 4H); ^{13}C NMR (126 MHz, $CDCl_3$) δ 161.8, 157.8, 136.1, 131.7, 127.2, 124.4, 121.8, 120.5, 111.8, 109.8, 102.6, 63.8, 53.1, 43.8; IR (neat) 2807, 1586 cm^{-1}; HRMS (ESI) *m/z* calcd for $C_{17}H_{20}N_5$ $[M + H]^+$: 294.1713, found: 294.1713; HPLC purity = 99.6%.

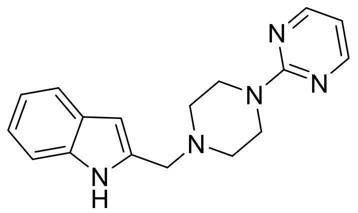

2-((4-(Pyrimidin-2-yl)piperazin-1-yl)methyl)-1H-indole (**44**): (1H-indol-2-yl)(4-(pyrimidin-2-yl)piperazin-1-yl)methanone **47** (0.0300 g, 0.0976 mmol, 1 equiv) and $LiAlH_4$ (2M in THF, 0.0110 g, 0.292, 3 equiv) were reacted according to general procedure F to afford **44** (0.0061 g, 21%) as a tan solid. Mp: 135–136 ˚C; ^{1}H NMR (500 MHz, $CDCl_3$) δ 8.35 – 8.30 (m, 3H), 7.57 (d, *J* = 7.9 Hz, 2H), 7.42 (d, *J* = 8.1 Hz, 1H), 7.21 – 7.18 (m, 1H), 7.12 – 7.09 (m, 1H), 6.59 – 6.54 (m, 1H), 6.45 – 6.42 (m, 1H), 3.98 – 3.89 (m, 4H), 3.81 – 3.71 (m, 2H), 2.80 – 2.66 (m, 4H); HRMS (ESI) *m/z* calcd for $C_{17}H_{20}N_5$ $[M + H]^+$: 294.1713, found: 294.1707; HPLC purity = 99.3%.

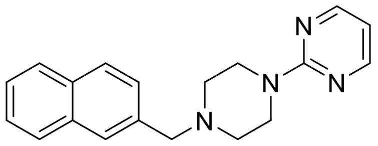

2-(4-(Naphthalen-2-ylmethyl)piperazin-1-yl)pyrimidine (**45**): 2-(Bromomethyl)naphthalene (0.0200 g, 0.09 mmol, 1 equiv), 1-(2-pyrimidyl)piperazine (0.0160 g, 0.100 mmol, 1.1 equiv), KI (0.015 g, 0.09 mmol, 1 equiv) and triethylamine (0.0180 g, 0.18 mmol, 2 equiv) were reacted according to general procedure C to afford **45** (0.0151 g, 55%) as an oil. TLC (5% MeOH/CH_2Cl_2): R_f= 0.5; ^{1}H NMR (500 MHz, $CDCl_3$) δ 8.31 (s, 2H), 7.91 – 7.82 (m, 4H), 7.59 (dd, *J* = 8.3, 1.8 Hz, 1H), 7.56 – 7.49 (m, 2H), 6.54 (t, *J* = 4.8 Hz, 1H), 4.12 (s, 2H), 4.08 (t, *J* = 5.2 Hz, 4H), 2.94 (t, *J* = 5.2 Hz, 4H); ^{13}C NMR (126 MHz, $CDCl_3$) δ 165.3, 161.2, 158.0, 133.5, 133.2, 130.3, 129.0, 128.1, 127.9,

127.8, 126.9, 126.8, 110.9, 61.8, 51.8, 41.9; IR (neat) 2807, 1581 cm^{-1}; HRMS (ESI) *m/z* calcd for $C_{19}H_{21}N_4$ $[M + H]^+$: 305.1766, found: 305.1788; HPLC purity >99.8 %.

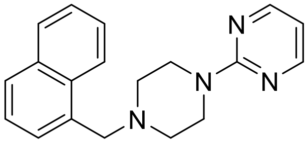

2-(4-(Naphthalen-1-ylmethyl)piperazin-1-yl)pyrimidine (**46**): 1-(Chloromethyl)naphthalene (0.0200 g, 0.11 mmol, 1 equiv), 1-(2-pyrimidyl)piperazine (0.0200 g, 0.12 mmol, 1.1 equiv), KI (0.019 g, 0.11 mmol, 1 equiv) and triethylamine (0.0230 g, 0.23 mmol, 2 equiv) were reacted according to general procedure C to afford **46** (0.0189 g, 55%) as an off-white solid. Mp: 105–107 ˚C; ^{1}H NMR (500 MHz, $CDCl_3$) δ 8.36 (d, *J* = 4.8 Hz, 1H), 8.32 (d, *J* = 4.8 Hz, 3H), 7.92 – 7.87 (m, 2H), 7.58 – 7.51 (m, 2H), 7.49 – 7.44 (m, 2H), 6.61 –6.48 (m, 2H), 3.89 – 3.77 (m, 4H), 2.69 – 2.55 (m, 4H); HRMS (ESI) *m/z* calcd for $C_{19}H_{21}N_4$ $[M + H]^+$: 305.1706, found: 305.1846; HPLC purity = 99.7%.

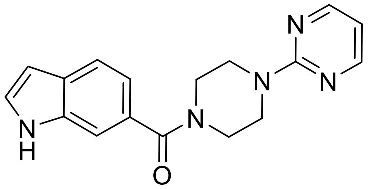

(1H-Indol-6-yl)(4-(pyrimidin-2-yl)piperazin-1-yl)methanone (**47**): Indole-6-carboxylic acid (0.0500 g, 0.31 mmol, 1 equiv), 1-(2-pyrimiyl)piperazine (0.0510 g, 0.31 mmol, 1 equiv), HATU (0.118 g, 0.31 mmol, 1 equiv) and DIEA (0.080 g, 0.62 mmol, 2 equiv) were reacted according to general procedure G to afford **47** (0.082 g, 86%) as a white solid. Mp: 218–220 ˚C; TLC (5% MeOH/CH_2Cl_2): R_f = 0.4; ^{1}H NMR (400 MHz, $CDCl_3$) δ 8.89 (s, 1H), 8.34 (d, *J* = 4.8 Hz, 2H), 7.65 (d, *J* = 8.2 Hz, 1H), 7.56 (d, *J* = 1.0 Hz, 1H), 7.29 (t, *J* = 2.8 Hz, 1H), 7.17 (dd, *J* = 8.1, 1.4 Hz, 1H), 6.56 (t, *J* = 4.8 Hz, 2H), 3.81 (d, *J* = 59.7 Hz, 8H); ^{13}C NMR (101 MHz, $CDCl_3$) δ 172.1, 157.8, 135.5, 129.4, 128.9, 126.5, 120.7, 118.9, 111.2, 110.5, 102.8, 44.2; IR (neat) 1586 cm^{-1}; HRMS (ESI) *m/z* calcd for $C_{17}H_{18}N_5O$ $[M + H]^+$: 308.1511, found: 308.1510; HPLC purity = 99.3%.

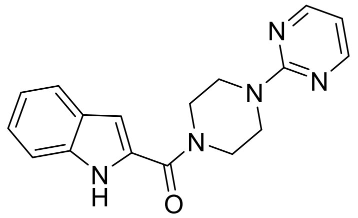

(1H-Indol-2-yl)(4-(pyrimidin-2-yl)piperazin-1-yl)methanone (**48**): Indole-2-carboxylic acid (0.200 g, 1.24 mmol, 1 equiv) was reacted with 1-(2-pyrimidyl)piperazine (0.204 g, 1.24 mmol, 1 equiv), HATU (0.471 g, 1.24 mmol, 1 equiv) and DIEA (0.321 g, 2.48 mmol, 2 equiv) were reacted according to general procedure G to afford **48** (0.350 g, 92%) as a white solid. Mp: 248–250 ˚C; TLC (5% MeOH/CH_2Cl_2): R_f = 0.7; ^{1}H NMR (400 MHz, $CDCl_3$) δ 9.28 (s, 1H), 8.36 (d, *J* = 4.8 Hz, 2H), 7.67 (dd, *J* = 8.1, 1.0 Hz, 1H), 7.44 (dd, *J* = 8.3, 1.0 Hz, 1H), 7.30 (ddd, *J* = 8.3, 7.0, 1.2 Hz, 1H), 7.15 (ddd, *J* = 8.0, 7.0, 1.0 Hz, 1H), 6.83 (dd, *J* = 2.2, 0.9 Hz, 1H), 6.58 (t, *J* = 4.8 Hz, 1H), 4.17 – 3.92 (m, 8H); ^{13}C NMR (101 MHz, $CDCl_3$) δ 162.8, 157.9, 135.8, 129.3, 127.7, 124.8, 122.1, 120.9, 111.9, 110.6, 105.6, 43.9, 36.6; IR (neat) 3238, 1582 cm^{-1}; HRMS (ESI) *m/z* calcd for $C_{17}H_{18}N_5O$ $[M + H]^+$: 308.1511, found: 308.1499; HPLC purity = 99.8%.

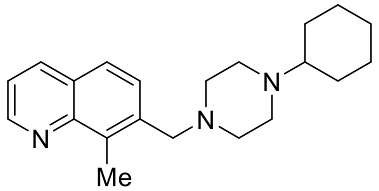

7-((4-Cyclohexylpiperazin-1-yl)methyl)-8-methylquinoline (**49**): (8-Methylquinolin-7-yl)methanol (0.0200 g, 0.115 mmol, 1 equiv), methanesulfonyl chloride (0.0250 g, 0.173 mmol, 1.5 equiv) and triethylamine (0.0180, 0.173 mmol, 1.5 equiv) were reacted according to general procedure D to afford the mesylated intermediate, which was reacted with 1-cyclohexylpiperidine (0.0230 g, 0.138 mmol, 1.2 equiv) according to general procedure D to afford **49** (0.00980 g, 26%) as a white solid. Mp: 77–79 ˚C; TLC (5% MeOH/CH_2Cl_2): R_f = 0.3; ^{1}H NMR (500 MHz, $CDCl_3$) δ 8.93 (dd, *J* = 4.2, 1.8 Hz, 1H), 8.11 (dd, *J* = 8.2, 1.8 Hz, 1H), 7.67 – 7.53 (m, 2H), 7.37 (dd, *J* = 8.2, 4.2 Hz, 1H), 3.72 (s, 2H), 2.84 (s, 3H), 2.59 (s, 7H), 2.24 (s, 1H), 1.90 (s, 2H), 1.78 (d, *J* = 10.3 Hz, 2H), 1.35 – 1.03 (m, 8H); ^{13}C NMR (126 MHz, $CDCl_3$) δ 149.3, 147.6, 137.5, 136.3, 135.9, 129.1, 127.4, 124.9, 120.6, 63.7, 60.9, 49.1, 29.9, 29.1, 26.4, 26.0, 13.2; IR (neat) 2922, 1453 cm$^-$

[1]; HRMS (ESI) *m/z* calcd for $C_{21}H_{30}N_3$ [M + H]$^+$: 324.2440, found: 324.2426; HPLC purity = 99.0%.

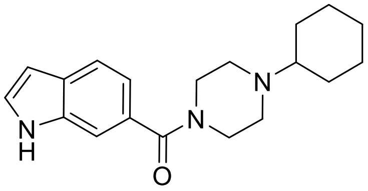

(4-Cyclohexylpiperazin-1-yl)(1H-indol-6-yl)methanone (**S-50**): Indole-6-carboxylic acid (0.0500 g, 0.31 mmol, 1 equiv), 1-cyclohexylpiperazine (0.0520 g, 0.31 mmol, 1 equiv), HATU (0.118 g, 0.31 mmol, 1 equiv) and DIEA (0.120 g, 0.93 mmol, 3 equiv) were reacted according to general procedure G to afford **S-50** (0.085 g, 88%) as a white solid. Mp: 235–237 ˚C; TLC (5% MeOH/CH_2Cl_2): R_f = 0.3; ^{1}H NMR (400 MHz, $CDCl_3$) δ 8.64 (s, 1H), 7.62 (dt, *J* = 8.1, 0.8 Hz, 1H), 7.51 (s, 1H), 7.34 – 7.27 (m, 1H), 7.14 (dd, *J* = 8.1, 1.4 Hz, 1H), 6.56 (ddd, *J* = 3.1, 2.0, 0.9 Hz, 1H), 3.77 (s, 4H), 2.62 (s, 4H), 2.36 (s, 1H), 1.85 (d, *J* = 36.3 Hz, 4H), 1.66 (s, 1H), 1.30 – 1.07 (m, 5H) ; ^{13}C NMR (101 MHz, $CDCl_3$) δ 171.4, 135.2, 129.1, 126.1, 120.5, 118.9, 110.9, 102.7, 100.0, 49.1, 28.6, 26.1, 25.7; IR (neat) 3155, 1600 cm^{-1}; HRMS (ESI) *m/z* calcd for $C_{19}H_{26}N_3O$ [M + H]$^+$: 312.2076, found: 312.2072.

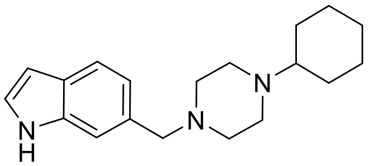

6-((4-Cyclohexylpiperazin-1-yl)methyl)-1H-indole (**50**): (4-Cyclohexylpiperazin-1-yl)(1H-indol-6-yl)methanone (**S-50**) (0.0200 g, 0.064 mmol, 1 equiv) and $LiAlH_4$ (2M in THF, 0.0070 g, 0.132, 3 equiv) were reacted according to general procedure F to afford **50** (0.0039 g, 20%) as a brown solid. Mp: 205–207 ˚C; ^{1}H NMR (500 MHz, $CDCl_3$) δ 8.27 (br s, 1H), 7.61 (d, *J* = 8.1 Hz, 1H), 7.28 – 7.26 (m, 1H), 7.24 (t, *J* = 3.0 Hz, 1H), 7.10 – 7.07 (m, 1H), 6.57 – 6.55 (m, 1H), 3.76 –3.69 (m, 2H), 3.65 (t, *J* = 5.1 Hz, 1H), 3.01 –2.80 (m, 4H), 2.98 – 2.76 (m, 2H), 2.62 – 2.60 (m, 2H), 1.91 – 1.80 (m, 4H), 1.32 – 1.10 (complex, 6H); HRMS (ESI) *m/z* calcd for $C_{19}H_{28}N_3$ [M + H]$^+$: 298.2278, found: 298.2282; HPLC purity > 99.8%.

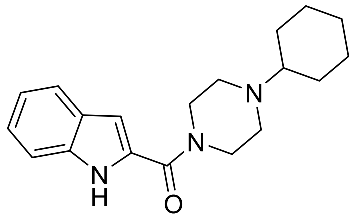

(4-Cyclohexylpiperazin-1-yl)(1H-indol-2-yl)methanone (**S-51**): Indole-2-carboxylic acid (0.0500 g, 0.31 mmol, 1 equiv), 1-cyclohexylpiperazine (0.0520 g, 0.31 mmol, 1 equiv), HATU (0.118 g, 0.31 mmol, 1 equiv) and DIEA (0.080 g, 0.62 mmol, 2 equiv) were reacted according to general procedure G to afford **S-51** (0.094 g, 97%) as a white solid. Mp: 213–215 ˚C; TLC (5% MeOH/CH_2Cl_2): R_f = 0.4; ^{1}H NMR (400 MHz, $CDCl_3$) δ 9.27 (s, 1H), 7.64 (dd, *J* = 8.0, 1.0 Hz, 1H), 7.42 (dd, *J* = 8.2, 1.0 Hz, 1H), 7.34 – 7.26 (m, 1H), 7.13 (ddd, *J* = 7.9, 7.0, 1.0 Hz, 1H), 6.78 (dd, *J* = 2.1, 0.9 Hz, 1H), 3.97 (s, 4H), 2.69 (t, *J* = 5.1 Hz, 4H), 2.37 (s, 1H), 1.95 – 1.78 (m, 4H), 1.65 (d, *J* = 12.8 Hz, 1H), 1.25 (td, *J* = 8.9, 8.3, 4.0 Hz, 4H), 1.17 – 1.02 (m, 1H); ^{13}C NMR (101 MHz, $CDCl_3$) δ 162.3, 135.7, 129.5, 127.7, 124.5, 122.0, 120.7, 111.8, 105.4, 64.0, 49.2, 28.9, 26.3, 25.9; IR (neat) 3255, 1595 cm^{-1}; HRMS (ESI) *m/z* calcd for $C_{19}H_{26}N_3O$ $[M + H]^+$: 312.2076, found: 312.2070.

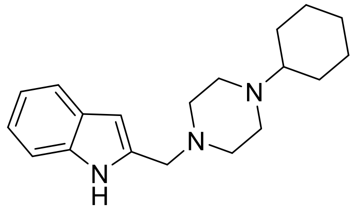

2-((4-Cyclohexylpiperazin-1-yl)methyl)-1H-indole (**51**): (4-cyclohexylpiperazin-1-yl)(1H-indol-2-yl)methanone **S-51** (0.0300 g, 0.096 mmol, 1 equiv) and $LiAlH_4$ (2M in THF, 0.0110 g, 0.289, 3 equiv) were reacted according to general procedure D to afford **50** (0.0011 g, 39%) as a yellow oil. TLC (5% MeOH/CH_2Cl_2): R_f = 0.4; ^{1}H NMR (500 MHz, $CDCl_3$) δ 8.65 (s, 1H), 7.55 (dq, *J* = 7.9, 0.9 Hz, 1H), 7.34 (dd, *J* = 8.1, 0.9 Hz, 1H), 7.14 (ddd, *J* = 8.2, 7.0, 1.2 Hz, 1H), 7.07 (ddd, *J* = 8.0, 7.1, 1.1 Hz, 1H), 6.36 (dd, *J* = 2.1, 0.9 Hz, 1H), 3.67 (d, *J* = 0.8 Hz, 2H), 2.63 (s, 3H), 2.56 (s, 4H), 2.28 (ddd, *J* = 10.3, 7.2, 3.6 Hz, 1H), 1.90 (dd, *J* = 7.3, 3.7 Hz, 2H), 1.79 (dd, *J* = 9.1, 4.0 Hz, 2H), 1.71 – 1.54 (m, 1H), 1.34 – 0.99 (m, 6H); ^{13}C NMR (126 MHz, $CDCl_3$) δ 136.3, 135.5, 128.4, 121.7, 120.3, 119.7, 110.9, 101.9, 63.7, 55.9, 53.6, 48.9, 29.0, 26.3, 26.0; IR (neat) 2928,

1455 cm^{-1}; HRMS (ESI) *m/z* calcd for $C_{19}H_{28}N_3$ $[M + H]^+$: 298.2283, found: 298.2275; HPLC purity > 99.8%.

Original Uncropped Blot as seen in Figure 1C

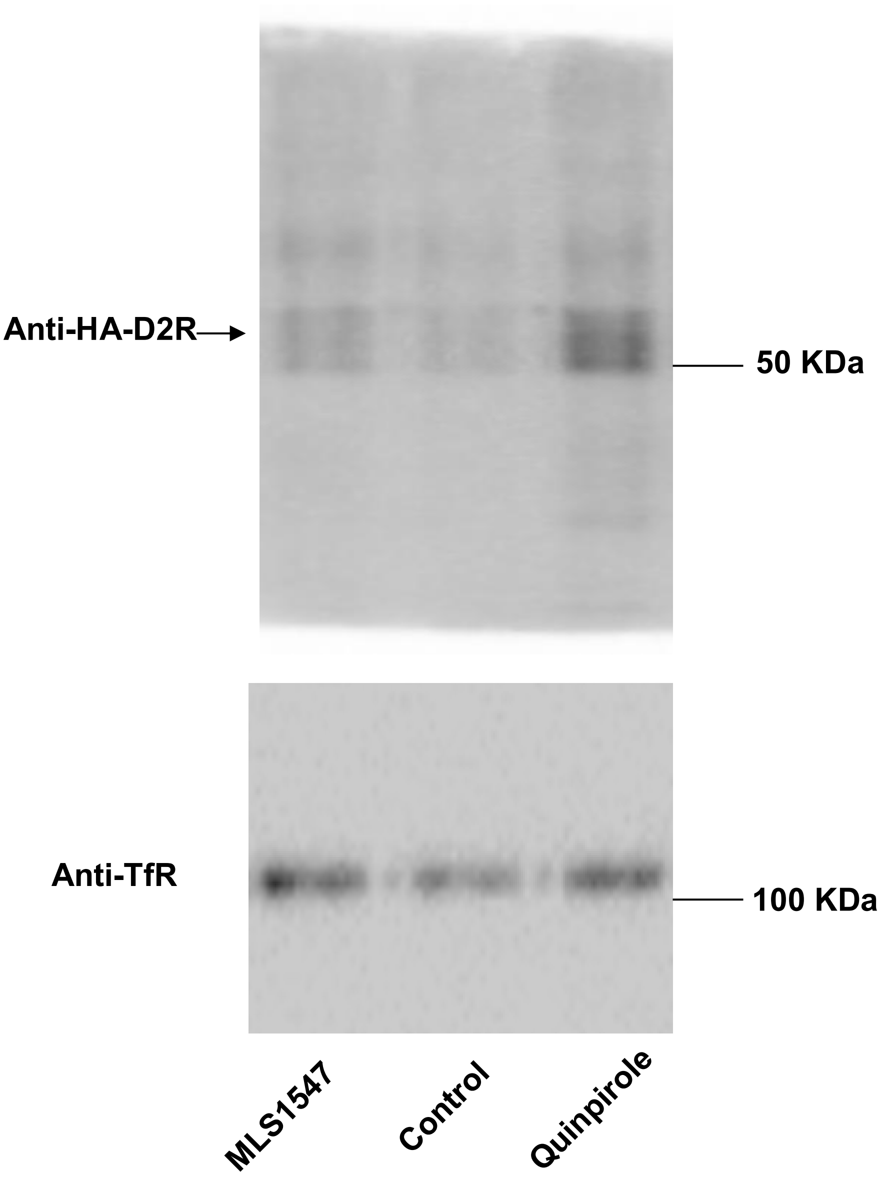

Blot of Transfected and Untransfected Cells Showing Specificity of Anti-HA Antibody

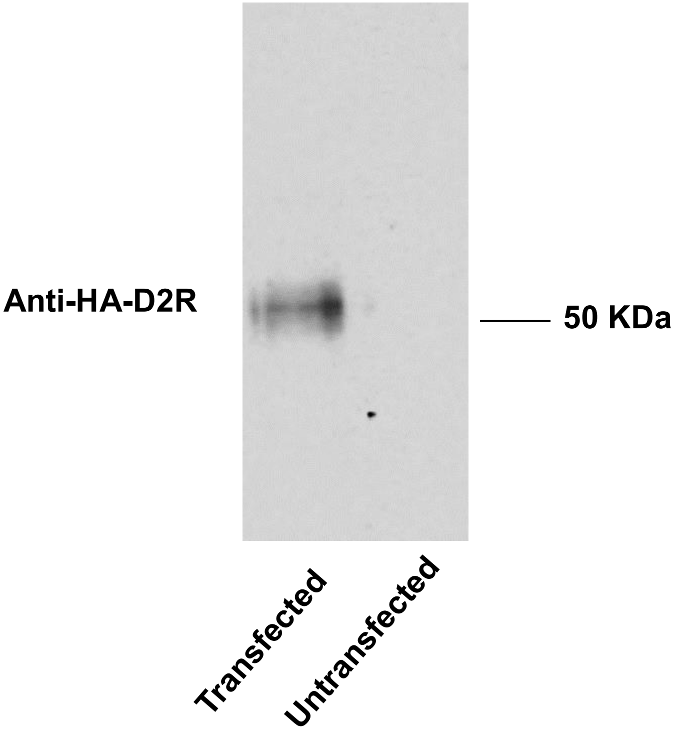

[1] Matsugi, M.; Tabusa, F.; Minamikawa, J.-i. (2000). Doebner–Miller synthesis in a two-phase system: practical preparation of quinolines. *Tetrahedron Lett. 41*, 8523–8525.

[2] Butini, S.; Campiani, G.; Franceschini, S.; Trotta, F.; Kumar, V.; Guarino, E.; Borrelli, G.; Fiorini, I.; Novellino, E.; Fattorusso, C. (2010) Discovery of bishomo (hetero) arylpiperazines as novel multifunctional ligands targeting dopamine D3 and serotonin 5-HT1A and 5-HT2A receptors. *J. Med. Chem. 53*, 4803–4807.

[3] Free, R.B., Chun, L.S., Moritz, A.E., Miller, B.N., Doyle, T.B., Conroy, J.L., Padron, A., Meade, J.A., Xiao, J., Hu, X., Dulcey, A.E., Han, Y., Duan, L., Titus, S., Bryant-Genevier, M., Barnaeva, E., Ferrer, M., Javitch, J.A., Beuming, T., Shi, L., Southall, N.T., Marugan, J.J., and Sibley, D.R. (2014). Discovery and characterization of a G protein-biased agonist that inhibits beta-arrestin recruitment to the D2 dopamine receptor. *Mol Pharmacol* 86**,** 96–105.
